# Supplementary material for: Targeting ATP2B1 impairs PI3K/Akt/FOXO signaling and reduces SARS-COV-2 infection and replication
Source: EMBO Rep. 2024 May 30;25(7):12. doi: 10.1038/s44319-024-00164-z (PMC11239940; doi:10.1038/s44319-024-00164-z)
Supplement: Supplementary file 1 — Appendix [file 44319_2024_164_MOESM1_ESM.pdf]

Appendix table section

**Appendix table S1**	**Differentially expressed genes upon SARS-CoV-2 early infection in HEK-293T-ACE2 cells that take part in "Calcium Signaling".**	**Pag. 2-4**
**Appendix table S2**	**Rare coding pathogenic variants in the *ATP2B1* locus.**	**Pag. 5**
**Appendix table S3**	**Noncoding variants in the genomic region of the *ATP2B1* locus that act as "expression quantitative traits loci" (eQTLs).**	**Pag. 6-13**
**Appendix table S4**	**eQTLs SNPs for the *ATP2B1* gene from the "Genotype-Tissue Expression" (GTex) database (P <1 ×10^{-6}).**	**Pag. 14-15**
**Appendix table S5**	**Differentially expressed genes in HEK-293T edited cells**	**Pag. 16-25**
**Appendix table S6**	**Differentially expressed proteins in HEK293T-ACE2 cells upon treatment with 1 µM compound #7 for 24 h.**	**Pag. 26-27**
**Appendix table S7**	**Proteins that take part in the interaction network, according to those downregulated by compound #7 in HEK293T-ACE2 cells.**	**Pag. 28**
**Appendix table S8**	**Proteins that take part in the interaction-network, according to those upregulated by compound #7 in HEK293T-ACE2 cells.**	**Pag. 29-31**
**Appendix table S9**	**Primers sequences for qPCR analyses**	**Pag. 32**

Appendix table S1. Differentially expressed genes upon SARS-CoV-2 early infection in HEK-293T-ACE2 cells that take part in "Calcium Signaling".

The "Ca^{2+}signaling by reactome pathway" through RNAseq analyses from the differentially expressed genes in HEK293T-ACE2 cells infected with SARS-CoV-2 (VOC Delta, 24 h) *versus* Mock infected cells. Here, 157 genes involved in Calcium Signaling were significantly deregulated by SARS-CoV-2 after 24 h. Data given include gene name, BaseMean (average of the normalized count values), log2FoldChange, lfcSE (standard error), pValue and adjusted p value (padj) are listed. Red, membrane and endoplasmic reticulum Ca^{2+} pumps (i.e., ATP2Bs and ATP2As, respectively).

Gene name	BaseMean	Log2FoldChange	lfcSE	pValue	pAdj
EGF	470.066.492.265.217	-40.619.336.960.114	0.664028018529976	7,08E+03	1,05E+05
CAMK1G	364.858.544.902.485	-340.754.771.922.581	0.913198249232395	8,34E+08	5,08E+09
TNNC2	201.384.809.825.337	-316.776.580.773.799	125.621.979.502.456	0.000426075476689886	0.00177089604329882
KDR	447.921.773.022.525	-291.789.785.352.181	0.690679824706131	1,20E+08	8,54E+08
PTGER3	2.631.393.772.017	-259.177.710.593.284	0.807666047432124	6,24E+09	0.000316375821514552
CACNA1I	869.350.624.213.802	-245.646.245.246.643	0.413068357989845	1,64E+04	2,27E+05
FGF19	595.183.107.293.085	-24.348.301.365.875	0.498409657835589	6,06E+06	5,46E+07
ATP2B2	270.270.787.313.473	-22.332.018.924.914	0.766386590862842	0.000168310855505058	0.000771084907017115
MYLK4	359.196.817.041.812	-218.249.512.123.712	0.653358723896032	4,44E+09	0.000231472468385037
CACNA1G	945.542.009.013.953	-193.464.303.503.756	0.183028207937104	2,77E-13	3,38E-12
NTRK3	280.665.734.128.966	-191.862.731.213.666	0.798849668970072	0.000742655141446291	0.00289573206235725
AVPR1A	486.965.123.732.544	-18.995.518.921.901	0.577352080972861	5,66E+09	0.00028951664775388
RYR2	163.422.835.354.659	-189.620.200.301.431	0.315137422842955	1,23E+04	1,74E+05
PRKCG	549.075.446.715.279	-177.755.730.324.405	0.188372347832613	3,09E-09	2,15E-06
RYR1	809.194.404.057.114	-169.311.919.230.691	0.486468803564657	3,24E+08	0.000174489336118528
PDGFRB	324.832.730.647.174	-166.275.196.192.471	0.771626078491638	0.00147511758120898	0.00532287697789966
NGF	301.570.589.660.459	-15.325.074.691.358	0.745790834556926	0.0020175651560619	0.00702354442142255
FGF18	298.564.044.685.259	-148.439.847.088.366	0.828292832261265	0.00337210418608626	0.0110347633464023
CALML6	490.099.528.433.961	-131.370.510.943.569	0.825860210839343	0.00533449786994513	0.0164946648727855
GRIN1	157.108.717.534.577	-128.763.563.304.787	0.323378819942514	6,46E+08	4,02E+09
NTRK2	262.623.726.884.291	-128.054.971.330.727	0.243831076403219	1,58E+06	1,58E+07
NTRK1	578.235.945.050.576	-12.596.871.921.419	0.551982314520049	0.00162927852165569	0.00581799505388285
GDNF	558.396.367.605.257	-125.541.837.244.765	0.531587287153423	0.00137309343890679	0.00500164697395093
PHKG1	399.138.091.587.535	-122.304.443.526.309	0.632694503323343	0.00358227204856374	0.0116168708830677
PTGFR	276.774.110.594.092	-116.245.612.041.348	0.826887731548613	0.00858417601832968	0.0248084910809525
ADRB2	103.466.364.639.335	-115.829.451.291.408	0.424783537150509	0.00059891519425065	0.0023882746914971
ATP2A1	203.108.013.385.034	-107.280.686.165.945	0.262329863906071	5,49E+08	3,46E+09
ERBB4	170.776.039.723.201	-104.381.160.003.265	0.300596363974115	6,48E+09	0.00032742177194106
CACNA1C	393.319.540.235.024	-102.106.063.979.435	0.192751646909679	1,72E+06	1,71E+07
ADRA1B	591.447.672.206.431	-101.281.999.512.882	0.511038582291448	0.00457889299727663	0.0144293335681128
SLC8A3	95.692.745.140.642	-0.998473384007727	0.410221783240345	0.00169667248287767	0.00602970050421849
PLCB4	853.137.293.137.909	-0.980318301180731	0.140257942056366	4,46E+01	9,22E+02
HRH1	274.954.013.122.704	-0.960466468918194	0.835252217871031	0.0151214999321587	0.0400493571921168
P2RX2	210.012.518.949.649	-0.906096933837229	0.255316025097521	6,31E+09	0.000319747150885531
CACNA1B	420.417.802.824.201	-0.887883543343921	0.188611910026867	4,54E+07	3,47E+07
GRIN2C	134.980.629.604.766	-0.861218744558696	0.31162870962008	0.000932072365025747	0.00354992600479506
SLC8A2	273.654.190.588.128	-0.858567119679333	0.237358375185696	5,37E+08	0.000275901884465897
MST1	588.954.201.846.589	-0.829615004504173	0.243875246591338	0.000125608272597485	0.000592232918411347
MCOLN3	165.355.435.122.002	-0.827337601316473	0.128096033644867	2,26E+03	3,64E+04
PDGFB	116.038.571.818.564	-0.802905099711672	0.413428970321733	0.00785763338500944	0.0230062180714721
PLCD1	606.706.213.146.116	-0.73419217381745	0.211338432292575	0.000128480351249161	0.000604624576401611
PTAFR	148.429.292.120.027	-0.718467868052912	0.305326705403937	0.00398084691805461	0.0127481951469379

SPHK1	425.399.369.213.307	-0.707202397121825	0.201780650419755	0.000116061152317301	0.000552590774509452
ADRB1	655.653.614.469.463	-0.679205605810754	0.472803117372689	0.025791562973466	0.0629923986317459
FLT1	700.355.766.267.359	-0.669898202668207	0.185104262705454	8,28E+09	0.000409305590749802
AGTR1	114.530.552.323.422	-0.656048536313823	0.390155781513432	0.0194109134979577	0.049664781084052
TPCN1	252.604.797.979.172	-0.600289817556319	0.132545750778705	2,04E+07	1,39E+09
PHKA2	175.224.737.675.515	-0.561183955695007	0.120614441069424	1,27E+08	8,99E+08
FGF2	107.171.213.660.927	-0.52864644155335	0.131546499596881	2,52E+09	0.000138420306528569
ERBB3	120.866.178.420.076	-0.526562320203576	0.13331201305227	3,35E+09	0.000179921804431245
CACNA1D	336.171.601.170.013	-0.513843498616443	0.231116856688361	0.0101629928909175	0.0285960922734542
FGF9	481.838.217.511.183	-0.440316618339933	0.201003686986393	0.0138044691557052	0.0370226603357302
ITPR3	544.035.684.154.367	-0.436844916967323	0.0858936274580685	2,85E+07	2,25E+07
BDKRB2	432.395.097.685.611	-0.433653663001909	0.497631594097063	0.0986511036863904	0.187638940694549
SLC8A1	522.452.995.997.531	-0.425640845179831	0.209128356637808	0.0207771460712456	0.0526173559586334
PDE1B	289.070.207.499.701	-0.410559888941243	0.206926593742145	0.0245441014890181	0.0604941914017028
VEGFA	297.506.769.867.281	-0.405780412554488	0.142594980006681	0.00247420124223248	0.00837757964418323
FGF5	79.709.394.383.223	-0.399984899482115	0.356896301574514	0.108219512397703	0.201880061965315
ATP2B4	283.800.562.649.639	-0.394169016228452	0.126855884293042	0.00112236419580121	0.00418746949373256
RET	577.232.271.862.126	-0.353170332880125	0.170689077431222	0.0239340673097788	0.0592338298112563
PDGFA	579.364.106.716.877	-0.322941855478592	0.173196187829469	0.0411391142990746	0.0926751594433211
CCKBR	126.784.008.566.849	-0.320952603632085	0.304055604871576	0.161838087141715	0.276984470866739
LTB4R2	189.584.854.807.253	-0.313758791468503	0.26937952269752	0.146164169708267	0.255529602755143
MCOLN2	113.978.857.145.436	-0.301788122475556	0.137806208277608	0.0203855557780396	0.0517490197933601
PRKCB	25.235.847.386.427	-0.29501851474154	0.109946339948008	0.00539312477447984	0.0166518138449238
CAMK2B	772.906.926.209.551	-0.283029002203993	0.145730810888778	0.0383029245794291	0.0874986769446822
PDGFRA	109.866.805.847.052	-0.282309320967817	0.2972729826783	0.211044616859362	0.340490350351324
CHRM3	560.839.356.409.002	-0.251888870437173	0.160119800958921	0.088376618755959	0.1719817480213
FGF17	16.496.815.745.705	-0.248096374729555	0.249482103006665	0.227384140460309	0.359420066595756
ADCY7	599.531.283.129.749	-0.242677303099414	0.155103300744442	0.0923579071335468	0.178067513485704
HTR6	354.992.109.224.363	-0.236382187781594	0.198583734709514	0.179234191122456	0.300143455269253
NOS2	382.743.199.985.029	-0.216942472653685	0.216414799478264	0.246028914267307	0.382173021389535
CAMK1D	115.738.679.551.248	-0.20243741585522	0.144915532772324	0.1364996988295	0.242143975557365
PTK2B	3.347.654.773.982	-0.198541912471893	0.198825455813247	0.259322322212717	0.397481473680127
PLCG2	128.718.181.153.958	-0.146004446908614	0.13411235660135	0.248733561997342	0.385274541913544
CALML4	720.412.591.192.242	-0.142650941830901	0.163982197735486	0.344385374689041	0.489805705022451
OXTR	148.277.035.788.061	-0.141149910723466	0.274436307642278	0.508031589930453	0.648622564287605
CHRNA7	489.354.993.796.243	-0.137351331277486	0.16799105529875	0.373336159701828	0.519360460101477
ITPR1	194.448.959.208.382	-0.130765692109194	0.135837801130851	0.309257090522638	0.45335357342098
PHKA1	179.591.123.826.724	-0.125547564416148	0.103400902226699	0.210340488576319	0.339696463309899
ADORA2A	223.762.626.919.609	-0.106559430507992	0.419538375673638	0.476481309897874	0.620376663464722
PLCG1	699.101.491.751.449	-0.104642450208333	0.0865004261930556	0.215520564598944	0.345427330349358
FGFR4	208.232.993.177.581	-0.101715568146048	0.102865746174184	0.308028355572787	0.452073453570876
FGFR1	499.106.216.692.943	-0.0923093100011115	0.0949481980581863	0.304396825481386	0.448217896866103
PHKG2	314.206.921.479.836	-0.0911508288462413	0.124077121364634	0.44208383855371	0.587814337568264
ADCY9	235.254.942.195.309	-0.0837295765189726	0.0974106513943221	0.376355915316127	0.522388753677872
FGF8	464.562.300.432.542	-0.0761848939011496	0.353716413037013	0.713462371093803	0.813931531496335
ATP2B3	587.586.125.763.348	-0.0626210328757565	0.439048123471953	0.0738189363850452	0.14885884758557
CXCR4	122.446.292.826.757	-0.0603388356624955	0.143329518789903	0.655120311029227	0.76759630744944
PLCB1	994.674.848.651.785	-0.0589275381309039	0.14056687275296	0.656669719144886	0.768689888118064
MST1R	165.032.670.287.062	-0.0494471966447024	0.245291459692777	0.802995262899111	0.878339556478466
CAMK4	891.013.810.454.115	-0.0452814478563072	0.177434779926719	0.778334345320621	0.861379840200812
STIM2	95.701.248.136.539	-0.0390808211180951	0.140900346772734	0.76820715039287	0.854282496755984
PLCE1	107.195.402.497.356	-0.0341801457720718	0.145079723037892	0.801377847150615	0.87713678071911
ITPKC	70.396.087.492.243	-0.0284050996316445	0.143515642077651	0.835382348472484	0.901049822420575
ADCY1	190.640.441.243.435	-0.0234347315901111	0.129135611085778	0.848319232392113	0.909983793161546
PPP3CC	594.164.875.490.275	-0.0217191364607527	0.151752406462586	0.877965754946522	0.929456415592496
ATP2A3	57.861.264.377.674	-0.0129319930116038	0.154240920271403	0.926149963731589	0.958988807760499
CACNA1H	345.963.329.850.669	-0.0119216426741543	0.105863576682641	0.924981441177746	0.958205598411089
GRIN2D	787.806.074.900.388	-0.00313600612645953	0.165848797099175	0.982047635092185	0.988873258407805
ORAI3	669.964.822.841.849	-0.0029585841913774	0.154784193674808	0.980434132269063	0.987886621158221
PRKACA	511.052.546.711.711	8,08E+09	0.0944525021842156	0.992207464272	0.995423498362367
MYLK	499.839.296.301.556	0.00232286697512634	0.18351714058263	0.990738415795618	0.99448260200729
FLT4	409.216.446.721.523	0.0279541004301016	0.0953640134258976	0.785110815522244	0.865849292306412
ITPR2	185.378.863.744.044	0.02851082365659	0.141313453931377	0.830830585455922	0.898094050005188

MYLK3	403.912.378.245.326	0.037299870072606	0.345347448460966	0.861313219529031	0.918180856193811
PRKCA	370.983.476.105.691	0.0516584120737146	0.124335893152742	0.665347986001177	0.776105971436831
ASPH	570.280.921.671.974	0.0628042195081283	0.0836495719479821	0.418065723667828	0.564821627755414
MCOLN1	854.203.446.687.649	0.0870758272799398	0.128577790062911	0.478726811743744	0.622464822939255
STIM1	279.598.609.555.574	0.0889388752105342	0.0880916879107226	0.302797288672749	0.446274957562273
F2R	166.748.356.309.198	0.110639447436645	0.110393117808017	0.300908480333341	0.444224780302863
EDNRA	206.182.690.976.498	0.11977237588773	0.239855306412242	0.583076385014544	0.710293050835899
ATP2B1	461.626.628.987.639	0.138005263348501	0.104312447327456	0.173826346731307	0.29304324553352
PLCB2	489.493.216.881.739	0.148460461120645	0.357200444519728	0.498085877010279	0.64013790333965
EGFR	183.157.545.910.826	0.15414471676835	0.147426537239317	0.26253090096408	0.401076892043935
CAMK2G	308.856.140.894.947	0.161321439499772	0.0861961767053738	0.0560495627231519	0.119042800166328
TPCN2	962.925.542.934.949	0.163688229634313	0.127735585791043	0.180017322232568	0.301137783686921
MCU	232.326.143.842.897	0.221045523640019	0.0948496793645021	0.0166832275403525	0.0435548197581096
TBXA2R	2.791.255.953.813	0.221920495895446	0.210650643728027	0.22896567920061	0.361286110716458
GNAQ	335.459.683.975.552	0.224240766827919	0.119403166128341	0.0505041469254608	0.109429738958333
VEGFC	262.865.606.384.951	0.252700671951347	0.227767993537088	0.195701724209886	0.321311440786427
MET	320.453.265.120.091	0.266669221254998	0.140882757374689	0.0439163404621055	0.0976594709508658
GNAL	167.298.061.304.345	0.276532654299546	0.114951656898321	0.012100979045169	0.0331554640153218
PDGFC	851.803.632.271.765	0.277270899831492	0.134986267423683	0.0305866358113616	0.0727059910065298
PLCD3	169.699.382.882.478	0.281311858492587	0.120781708407388	0.0150483628998168	0.0398840609001914
ITPKA	31.922.290.961.536	0.299758123828119	0.236373895471205	0.133703915861153	0.238283254794966
ORAI2	198.370.882.017.582	0.307664239516031	0.120832479310531	0.00778793820930639	0.0228296793309466
ATP2A2	153.064.511.889.483	0.315242341768931	0.0910620865403779	0.000357803271130382	0.00151709402930254
P2RX4	670.723.664.696.776	0.319126310522452	0.166028279387448	0.03684946883754	0.0846902290966677
CAMK2D	277.091.778.625.274	0.349630625956543	0.110798494640924	0.00106991166438658	0.00401189901582001
PLCB3	30.578.336.330.819	0.369227730401235	0.0885761252993303	1,91E+09	0.000108029201407511
PPP3CA	256.585.595.024.188	0.373235392209162	0.12771848546183	0.00217807704361912	0.00749461396054691
PRKACB	155.479.632.445.332	0.382557095457931	0.152796328493714	0.0077530204829596	0.0227452518074045
ADCY3	35.157.160.555.465	0.384570165506337	0.10318861828844	0.00011816572969351	0.000561384634799467
SLC25A4	222.886.070.310.512	0.426581089118532	0.129454666583038	0.000538905641752702	0.00217817459658416
ORAI1	109.532.355.302.949	0.430951303438528	0.149874442423144	0.00215782305621755	0.00743753904020853
ERBB2	466.302.801.935.591	0.453000747759762	0.0859070335582125	7,17E+06	6,35E+07
PHKB	335.349.384.106.186	0.459366904885841	0.110460336516651	1,67E+07	9,52E+09
CALM1	120.000.433.254.103	0.473988326599753	0.0822585983329827	4,03E+05	4,40E+06
FGFR3	412.173.682.384.824	0.478844958517372	0.0926416266480472	1,18E+07	1,01E+08
GNAS	432.993.639.253.567	0.505876338060198	0.0933872474831993	2,81E+06	2,69E+07
ADORA2B	662.365.757.868.432	0.555313439236578	0.153468486520173	0.000115645342097655	0.000550846120311868
PPP3R1	311.618.175.694.851	0.55631882811167	0.11382605476247	4,12E+07	3,18E+08
GNA11	419.075.116.639.903	0.557660312687605	0.114087597573777	4,22E+07	3,24E+08
SLC25A5	31.554.050.213.949	0.591996841533849	0.0944225412316923	1,38E+03	1,94E+05
VDAC2	128.821.388.697.325	0.59427964544448	0.105051143244051	5,78E+04	6,16E+06
FGFR2	282.071.577.743.889	0.614594852851092	0.10179451505322	5,45E+04	6,90E+05
SPHK2	990.013.366.279.113	0.617877886815798	0.16358013542891	5,28E+08	0.00027165538890307
CALM2	139.947.481.397.403	0.658786203542567	0.0878843057258299	1,66E+00	4,31E+01
VDAC3	615.721.313.965.366	0.659604486969271	0.0792079673505408	2,75E-03	1,01E-01
VEGFB	182.794.381.075.822	0.66583541826766	0.128523584142247	6,82E+06	6,07E+07
SLC25A6	110.045.490.085.772	0.697750300545649	0.132019257663235	2,62E+06	2,52E+07
PPP3CB	282.723.784.431.472	0.737736854234094	0.1037294597725	3,04E+01	6,49E+02
CALM3	237.389.641.209.479	0.740184385222163	0.070241951160023	3,98E-12	4,23E-10
VDAC1	127.948.228.767.543	0.798029346731346	0.0882435705879339	2,97E-06	1,58E-04
CAMK1	302.929.731.805.388	0.832051455658237	0.114559291649818	8,35E+00	1,95E+02

Appendix table S2. Rare coding pathogenic variants in the *ATP2B1* locus.

Among the 351 coding variants of *ATP2B1* from "The Genome Aggregation Database" (GnomAD v2.1), 13 are reported as "pathogenic" according to "Functional Analysis through Hidden Markov Models" (FATHMM) prediction scores. These variants are rare and were thus excluded from further investigations. Data given include chromosomal locus, position, rsID, dbSNP ID, protein consequence, fathmm prediction, reference and alternative alleles, transcript consequence, variant annotation, and allele frequency.

Chr	Position	rsID	dbSNP	ID	Protein	FATHMM prediction	Ref	Alt	Transcript consequence	Annotation	Allele frequency
12	89984832	rs115449702	rs115449702	NP_001673.2	I1198V	DAMAGING	T	C	c.3592A>G	missense_variant	7,96096E-06
12	89984882	rs142404163	rs142404163	NP_001673.2	P1181H	DAMAGING	G	T	c.3542C>A	missense_variant	0,000453695
12	89995138	rs79015625	rs79015625	NP_001001323.1	I1045V	DAMAGING	T	C	c.3133A>G	missense_variant	0,000900653
12	89997018	rs189003061	rs189003061	NP_001673.2	F954L	DAMAGING	A	T	c.2862T>A	missense_variant	4,24257E-06
12	90005121	rs182212584	rs182212584	NP_001001323.1	R699T	DAMAGING	C	G	c.2096G>C	missense_variant	3,00153E-05
12	90010584	rs145288143	rs145288143	NP_001001323.1	P688T	DAMAGING	G	T	c.2062C>A	missense_variant	1,99351E-05
12	90013932	rs199582675	rs199582675	NP_001673.2	R558Q	DAMAGING	C	T	c.1673G>A	missense_variant	3,98137E-06
12	90018046	rs184588664	rs184588664	NP_001673.2	F420L	DAMAGING	A	G	c.1258T>C	missense_variant	3,98096E-06
12	90018088	rs116585190	rs116585190	NP_001001323.1	A406S	DAMAGING	C	A	c.1216G>T	missense_variant	0,000159293
12	90028654	rs149203241	rs149203241	NP_001001323.1	L230F	DAMAGING	G	C	c.688C>G	missense_variant	4,00208E-06
12	90036125	rs200832650	rs200832650	NP_001001323.1	S72R	DAMAGING	A	T	c.216T>A	missense_variant	8,22788E-06
12	90049575	rs116694524	rs116694524	NP_001001323.1	T30M	DAMAGING	G	A	c.89C>T	missense_variant	0,000767591
12	90049584	rs201371800	rs201371800	NP_001001323.1	F27Y	DAMAGING	A	T	c.80T>A	missense_variant	3,97712E-06

Appendix table S3. Noncoding variants in the genomic region of the *ATP2B1* locus that act as "expression quantitative traits loci" (eQTLs).

In-silico analysis pipeline identifying the presence of noncoding variants in the ATP2B1 locus acting as expression quantitative traits. Data given include genecode ID, gene symbol, variant ID (chromosomal position, alternative and reference alleles), SNP ID, P-value, normalized enrichment score (NES) and tissue (using the "Genotype-Tissue Expression" GTex database).

Gencode Id	Gene symbol	Variant Id	SNP Id	P-value	NES	Tissue
ENSG00000070961.15	ATP2B1	chr12_89615182_A_G_b38	rs2681472	1,60E-18	0,35	Artery - Tibial
ENSG00000070961.15	ATP2B1	chr12_89619312_T_C_b38	rs2681492	3,40E-18	0,35	Artery - Tibial
ENSG00000070961.15	ATP2B1	chr12_89632685_G_A_b38	rs11105352	3,60E-18	0,35	Artery - Tibial
ENSG00000070961.15	ATP2B1	chr12_89632686_C_A_b38	rs11105353	3,60E-18	0,35	Artery - Tibial
ENSG00000070961.15	ATP2B1	chr12_89632746_A_G_b38	rs11105354	3,60E-18	0,35	Artery - Tibial
ENSG00000070961.15	ATP2B1	chr12_89656726_A_G_b38	rs12579302	3,60E-18	0,35	Artery - Tibial
ENSG00000070961.15	ATP2B1	chr12_89665065_G_A_b38	rs111478946	3,60E-18	0,35	Artery - Tibial
ENSG00000070961.15	ATP2B1	chr12_89675499_T_G_b38	rs11105364	3,60E-18	0,35	Artery - Tibial
ENSG00000070961.15	ATP2B1	chr12_89680664_G_C_b38	rs11105368	7,40E-18	0,34	Artery - Tibial
ENSG00000070961.15	ATP2B1	chr12_89681466_C_T_b38	rs4842675	7,40E-18	0,34	Artery - Tibial
ENSG00000070961.15	ATP2B1	chr12_89591515_AAAGT_A_b38	rs10580742	1,20E-17	0,35	Artery - Tibial
ENSG00000070961.15	ATP2B1	chr12_89696964_C_T_b38	rs11105378	1,30E-17	0,34	Artery - Tibial
ENSG00000070961.15	ATP2B1	chr12_89666809_G_A_b38	rs17249754	1,50E-17	0,34	Artery - Tibial
ENSG00000070961.15	ATP2B1	chr12_89668599_G_A_b38	rs6538195	1,50E-17	0,34	Artery - Tibial
ENSG00000070961.15	ATP2B1	chr12_89678299_G_A_b38	rs73437358	1,50E-17	0,34	Artery - Tibial
ENSG00000070961.15	ATP2B1	chr12_89625401_C_G_b38	rs57481061	1,50E-17	0,34	Artery - Tibial
ENSG00000070961.15	ATP2B1	chr12_89548613_A_G_b38	rs11105328	1,70E-17	0,34	Artery - Tibial
ENSG00000070961.15	ATP2B1	chr12_89547772_T_C_b38	rs4842666	2,10E-17	0,34	Artery - Tibial
ENSG00000070961.15	ATP2B1	chr12_89694803_T_A_b38	rs11105375	5,70E-17	0,34	Artery - Tibial
ENSG00000070961.15	ATP2B1	chr12_89697090_A_G_b38	rs12230074	8,10E-17	0,33	Artery - Tibial
ENSG00000070961.15	ATP2B1	chr12_89660842_T_C_b38	rs73437338	1,50E-16	0,33	Artery - Tibial
ENSG00000070961.15	ATP2B1	chr12_89597536_C_T_b38	rs117742247	4,20E-16	0,34	Artery - Tibial
ENSG00000070961.15	ATP2B1	chr12_89599730_A_T_b38	rs11105337	4,60E-16	0,33	Artery - Tibial
ENSG00000070961.15	ATP2B1	chr12_89694991_G_A_b38	rs11105376	7,80E-16	0,33	Artery - Tibial
ENSG00000070961.15	ATP2B1	chr12_89709925_C_G_b38	rs2280715	4,30E-14	0,31	Artery - Tibial
ENSG00000070961.15	ATP2B1	chr12_89713495_T_C_b38	rs11105382	4,30E-14	0,31	Artery - Tibial
ENSG00000070961.15	ATP2B1	chr12_89713529_T_C_b38	rs11105383	4,30E-14	0,31	Artery - Tibial
ENSG00000070961.15	ATP2B1	chr12_89719293_T_G_b38	rs7299436	4,30E-14	0,31	Artery - Tibial
ENSG00000070961.15	ATP2B1	chr12_89709168_C_G_b38	rs73437382	8,00E-14	0,3	Artery - Tibial
ENSG00000070961.15	ATP2B1	chr12_89698005_G_C_b38	rs4842676	6,10E-13	-0,27	Artery - Tibial
ENSG00000070961.15	ATP2B1	chr12_89701396_T_C_b38	rs11105379	1,40E-12	0,3	Artery - Tibial
ENSG00000070961.15	ATP2B1	chr12_89695013_G_A_b38	rs10858917	2,50E-12	-0,26	Artery - Tibial
ENSG00000070961.15	ATP2B1	chr12_89556543_A_C_b38	rs7302816	4,50E-12	0,25	Artery - Tibial
ENSG00000070961.15	ATP2B1	chr12_89713058_A_G_b38	rs11105381	2,90E-11	-0,25	Artery - Tibial
ENSG00000070961.15	ATP2B1	chr12_89715560_T_C_b38	rs2408046	5,50E-11	-0,24	Artery - Tibial
ENSG00000070961.15	ATP2B1	chr12_89703890_G_C_b38	rs10745511	5,70E-11	-0,25	Artery - Tibial
ENSG00000070961.15	ATP2B1	chr12_89705620_T_A_b38	rs2113894	5,70E-11	-0,25	Artery - Tibial
ENSG00000070961.15	ATP2B1	chr12_89696509_CAA_C_b38	rs10550903	7,60E-11	-0,23	Artery - Tibial
ENSG00000070961.15	ATP2B1	chr12_89738370_G_A_b38	rs12579052	5,40E-10	0,28	Artery - Tibial
ENSG00000070961.15	ATP2B1	chr12_90001084_A_T_b38	rs4408360	3,40E-08	-0,18	Esophagus - Mucosa
ENSG00000070961.15	ATP2B1	chr12_90024395_T_G_b38	rs149193253	4,40E-08	1	Brain - Anterior cingulate cortex (BA24)
ENSG00000070961.15	ATP2B1	chr12_90049701_T_C_b38	rs150184539	4,40E-08	1	Brain - Anterior cingulate cortex (BA24)
ENSG00000070961.15	ATP2B1	chr12_90137707_G_A_b38	rs77187603	4,40E-08	1	Brain - Anterior cingulate cortex (BA24)
ENSG00000070961.15	ATP2B1	chr12_90143905_A_G_b38	rs150119566	4,40E-08	1	Brain - Anterior cingulate cortex (BA24)
ENSG00000070961.15	ATP2B1	chr12_89677042_CAAAG_C_b38	rs143087380	5,00E-08	-0,17	Artery - Tibial
ENSG00000070961.15	ATP2B1	chr12_90006794_TA_T_b38	rs67882152	8,80E-08	-0,18	Esophagus - Mucosa
ENSG00000070961.15	ATP2B1	chr12_90006796_A_T_b38	rs114744356	8,80E-08	-0,18	Esophagus - Mucosa
ENSG00000070961.15	ATP2B1	chr12_90010274_T_C_b38	rs7488223	1,10E-07	-0,17	Esophagus - Mucosa
ENSG00000070961.15	ATP2B1	chr12_89973923_G_C_b38	rs4842701	1,30E-07	-0,17	Esophagus - Mucosa
ENSG00000070961.15	ATP2B1	chr12_89997003_T_C_b38	rs7968803	1,30E-07	-0,18	Esophagus - Mucosa

ENSG00000070961.15	ATP2B1	chr12_89991517_A_G_b38	rs10777217	1,60E-07	-0,17	Esophagus - Mucosa
ENSG00000070961.15	ATP2B1	chr12_89595822_G_A_b38	rs1401982	2,30E-07	-0,16	Artery - Tibial
ENSG00000070961.15	ATP2B1	chr12_89576142_A_AACTC_b38	rs10689649	2,50E-07	0,16	Artery - Tibial
ENSG00000070961.15	ATP2B1	chr12_89968275_A_G_b38	rs4466894	2,70E-07	-0,17	Esophagus - Mucosa
ENSG00000070961.15	ATP2B1	chr12_89584456_C_T_b38	rs1689040	2,80E-07	0,16	Artery - Tibial
ENSG00000070961.15	ATP2B1	chr12_89951830_C_T_b38	rs4842699	3,00E-07	-0,17	Esophagus - Mucosa
ENSG00000070961.15	ATP2B1	chr12_89631845_G_A_b38	rs2681485	3,30E-07	-0,16	Artery - Tibial
ENSG00000070961.15	ATP2B1	chr12_90140138_C_T_b38	rs55849728	3,80E-07	-0,18	Esophagus - Mucosa
ENSG00000070961.15	ATP2B1	chr12_89571272_C_T_b38	rs7313874	3,90E-07	0,16	Artery - Tibial
ENSG00000070961.15	ATP2B1	chr12_89434814_A_G_b38	rs8181784	4,20E-07	0,17	Artery - Tibial
ENSG00000070961.15	ATP2B1	chr12_89435693_C_T_b38	rs11105287	4,20E-07	0,17	Artery - Tibial
ENSG00000070961.15	ATP2B1	chr12_89569762_G_GAGA_b38	rs35533953	4,20E-07	0,16	Artery - Tibial
ENSG00000070961.15	ATP2B1	chr12_89687411_T_C_b38	rs7136259	5,00E-07	-0,16	Artery - Tibial
ENSG00000070961.15	ATP2B1	chr12_89702568_T_C_b38	rs10858918	5,00E-07	-0,17	Artery - Tibial
ENSG00000070961.15	ATP2B1	chr12_89943269_A_C_b38	rs4842697	5,20E-07	-0,17	Esophagus - Mucosa
ENSG00000070961.15	ATP2B1	chr12_89436943_T_C_b38	rs10777163	6,60E-07	0,17	Artery - Tibial
ENSG00000070961.15	ATP2B1	chr12_89643729_T_C_b38	rs111337717	7,20E-07	0,35	Artery - Tibial
ENSG00000070961.15	ATP2B1	chr12_89713948_C_T_b38	rs1590008	7,20E-07	-0,16	Artery - Tibial
ENSG00000070961.15	ATP2B1	chr12_89717005_G_A_b38	rs1980235	7,20E-07	-0,16	Artery - Tibial
ENSG00000070961.15	ATP2B1	chr12_90156621_AG_A_b38	rs369917221	7,60E-07	0,75	Artery - Tibial
ENSG00000070961.15	ATP2B1	chr12_90025415_G_A_b38	rs10858944	8,30E-07	-0,16	Esophagus - Mucosa
ENSG00000070961.15	ATP2B1	chr12_90034251_A_G_b38	rs73208536	8,40E-07	-0,16	Esophagus - Mucosa
ENSG00000070961.15	ATP2B1	chr12_90385245_G_A_b38	rs1594565	8,60E-07	0,38	Brain - Spinal cord (cervical c-1)
ENSG00000070961.15	ATP2B1	chr12_90047438_T_C_b38	rs10777221	8,70E-07	-0,16	Esophagus - Mucosa
ENSG00000070961.15	ATP2B1	chr12_90048224_G_A_b38	rs7488974	8,70E-07	-0,16	Esophagus - Mucosa
ENSG00000070961.15	ATP2B1	chr12_89987295_A_ATGTT_b38	rs10625779	9,80E-07	-0,16	Esophagus - Mucosa
ENSG00000070961.15	ATP2B1	chr12_89708115_T_A_b38	rs1358350	9,90E-07	-0,16	Artery - Tibial
ENSG00000070961.15	ATP2B1	chr12_89640625_G_GC_b38	rs66786558	0,0000011	-0,16	Artery - Tibial
ENSG00000070961.15	ATP2B1	chr12_89640630_T_C_b38	rs78409878	0,0000011	-0,16	Artery - Tibial
ENSG00000070961.15	ATP2B1	chr12_89648365_C_G_b38	rs11105358	0,0000011	-0,16	Artery - Tibial
ENSG00000070961.15	ATP2B1	chr12_90398938_C_T_b38	rs7955801	0,0000012	0,38	Brain - Spinal cord (cervical c-1)
ENSG00000070961.15	ATP2B1	chr12_90392860_A_C_b38	rs2116497	0,0000012	0,37	Brain - Spinal cord (cervical c-1)
ENSG00000070961.15	ATP2B1	chr12_89437093_C_T_b38	rs10858851	0,0000012	0,17	Artery - Tibial
ENSG00000070961.15	ATP2B1	chr12_90072266_A_G_b38	rs6538223	0,0000012	-0,16	Esophagus - Mucosa
ENSG00000070961.15	ATP2B1	chr12_90168240_C_T_b38	rs4842509	0,0000012	-0,17	Esophagus - Mucosa
ENSG00000070961.15	ATP2B1	chr12_90170518_A_G_b38	rs17836934	0,0000014	-0,17	Esophagus - Mucosa
ENSG00000070961.15	ATP2B1	chr12_89482366_T_C_b38	rs11105298	0,0000015	0,17	Artery - Tibial
ENSG00000070961.15	ATP2B1	chr12_90391772_A_C_b38	rs7976895	0,0000016	0,37	Brain - Spinal cord (cervical c-1)
ENSG00000070961.15	ATP2B1	chr12_90393455_G_A_b38	rs12580873	0,0000016	0,37	Brain - Spinal cord (cervical c-1)
ENSG00000070961.15	ATP2B1	chr12_90084874_G_A_b38	rs10858948	0,0000016	-0,15	Esophagus - Mucosa
ENSG00000070961.15	ATP2B1	chr12_90079719_A_G_b38	rs6538224	0,0000017	-0,16	Esophagus - Mucosa
ENSG00000070961.15	ATP2B1	chr12_90376827_T_C_b38	rs11105589	0,0000018	0,37	Brain - Spinal cord (cervical c-1)
ENSG00000070961.15	ATP2B1	chr12_90386667_C_T_b38	rs6538234	0,0000018	0,37	Brain - Spinal cord (cervical c-1)
ENSG00000070961.15	ATP2B1	chr12_90388244_T_A_b38	rs7979392	0,0000018	0,37	Brain - Spinal cord (cervical c-1)
ENSG00000070961.15	ATP2B1	chr12_90390285_G_C_b38	rs2043213	0,0000018	0,37	Brain - Spinal cord (cervical c-1)
ENSG00000070961.15	ATP2B1	chr12_89526956_C_T_b38	rs11105312	0,0000018	0,16	Artery - Tibial
ENSG00000070961.15	ATP2B1	chr12_89527498_C_T_b38	rs10777185	0,0000018	0,16	Artery - Tibial
ENSG00000070961.15	ATP2B1	chr12_89625452_C_T_b38	rs7297206	0,0000018	-0,15	Artery - Tibial
ENSG00000070961.15	ATP2B1	chr12_90059201_T_C_b38	rs7959983	0,0000021	-0,16	Esophagus - Mucosa
ENSG00000070961.15	ATP2B1	chr12_90080698_G_A_b38	rs7485419	0,0000021	-0,16	Esophagus - Mucosa
ENSG00000070961.15	ATP2B1	chr12_90376071_T_C_b38	rs12582977	0,0000023	0,37	Brain - Spinal cord (cervical c-1)
ENSG00000070961.15	ATP2B1	chr12_89533400_G_A_b38	rs11105319	0,0000024	0,16	Artery - Tibial
ENSG00000070961.15	ATP2B1	chr12_89534012_A_G_b38	rs10858902	0,0000024	0,16	Artery - Tibial
ENSG00000070961.15	ATP2B1	chr12_89534195_G_A_b38	rs10858903	0,0000024	0,16	Artery - Tibial
ENSG00000070961.15	ATP2B1	chr12_89539669_T_C_b38	rs4842662	0,0000024	0,16	Artery - Tibial
ENSG00000070961.15	ATP2B1	chr12_89539986_G_C_b38	rs3741898	0,0000024	0,16	Artery - Tibial
ENSG00000070961.15	ATP2B1	chr12_89540697_C_T_b38	rs10858906	0,0000024	0,16	Artery - Tibial
ENSG00000070961.15	ATP2B1	chr12_89541074_CAGAG_C_b38	rs71897440	0,0000024	0,16	Artery - Tibial
ENSG00000070961.15	ATP2B1	chr12_89541195_G_C_b38	rs12369195	0,0000024	0,16	Artery - Tibial
ENSG00000070961.15	ATP2B1	chr12_89528255_C_A_b38	rs10858897	0,0000025	0,16	Artery - Tibial
ENSG00000070961.15	ATP2B1	chr12_89535128_A_G_b38	rs12369685	0,0000026	0,17	Artery - Tibial
ENSG00000070961.15	ATP2B1	chr12_90068229_T_C_b38	rs7310135	0,0000026	-0,15	Esophagus - Mucosa
ENSG00000070961.15	ATP2B1	chr12_90079906_G_A_b38	rs6419356	0,0000026	-0,15	Esophagus - Mucosa

ENSG00000070961.15	ATP2B1	chr12_90081169_G_A_b38	rs7962435	0,0000026	-0,15	Esophagus - Mucosa
ENSG00000070961.15	ATP2B1	chr12_90084513_C_T_b38	rs7132441	0,0000026	-0,15	Esophagus - Mucosa
ENSG00000070961.15	ATP2B1	chr12_89530034_G_C_b38	rs10858899	0,0000027	0,16	Artery - Tibial
ENSG00000070961.15	ATP2B1	chr12_89530596_A_ATCT_b38	rs112698976	0,0000027	0,16	Artery - Tibial
ENSG00000070961.15	ATP2B1	chr12_89530782_C_T_b38	rs11105314	0,0000027	0,16	Artery - Tibial
ENSG00000070961.15	ATP2B1	chr12_89531423_T_G_b38	rs11105316	0,0000027	0,16	Artery - Tibial
ENSG00000070961.15	ATP2B1	chr12_89933142_G_A_b38	rs11105466	0,0000028	-0,16	Esophagus - Mucosa
ENSG00000070961.15	ATP2B1	chr12_89432148_T_C_b38	rs10858848	0,0000029	0,16	Artery - Tibial
ENSG00000070961.15	ATP2B1	chr12_89497589_G_A_b38	rs11105302	0,000003	0,16	Artery - Tibial
ENSG00000070961.15	ATP2B1	chr12_89497697_T_G_b38	rs10858881	0,000003	0,16	Artery - Tibial
ENSG00000070961.15	ATP2B1	chr12_89441951_C_A_b38	rs10858854	0,0000032	0,17	Artery - Tibial
ENSG00000070961.15	ATP2B1	chr12_89445295_C_T_b38	rs11105291	0,0000032	0,17	Artery - Tibial
ENSG00000070961.15	ATP2B1	chr12_89445926_A_G_b38	rs10858860	0,0000032	0,17	Artery - Tibial
ENSG00000070961.15	ATP2B1	chr12_90049705_A_G_b38	rs10858945	0,0000032	-0,15	Esophagus - Mucosa
ENSG00000070961.15	ATP2B1	chr12_90050234_G_T_b38	rs7958749	0,0000032	-0,15	Esophagus - Mucosa
ENSG00000070961.15	ATP2B1	chr12_89481583_G_A_b38	rs75615848	0,0000035	0,32	Artery - Tibial
ENSG00000070961.15	ATP2B1	chr12_89439561_T_C_b38	rs10858852	0,0000036	0,16	Artery - Tibial
ENSG00000070961.15	ATP2B1	chr12_89440336_C_T_b38	rs4842653	0,0000036	0,16	Artery - Tibial
ENSG00000070961.15	ATP2B1	chr12_89440651_T_A_b38	rs4842654	0,0000036	0,16	Artery - Tibial
ENSG00000070961.15	ATP2B1	chr12_89442082_C_G_b38	rs10858855	0,0000036	0,16	Artery - Tibial
ENSG00000070961.15	ATP2B1	chr12_89442149_G_A_b38	rs9795772	0,0000036	0,16	Artery - Tibial
ENSG00000070961.15	ATP2B1	chr12_89442895_G_C_b38	rs11513812	0,0000036	0,16	Artery - Tibial
ENSG00000070961.15	ATP2B1	chr12_89443404_T_C_b38	rs11105289	0,0000036	0,16	Artery - Tibial
ENSG00000070961.15	ATP2B1	chr12_89445026_A_T_b38	rs11105290	0,0000036	0,16	Artery - Tibial
ENSG00000070961.15	ATP2B1	chr12_89445465_C_T_b38	rs10858859	0,0000036	0,16	Artery - Tibial
ENSG00000070961.15	ATP2B1	chr12_89445949_A_G_b38	rs10858861	0,0000036	0,16	Artery - Tibial
ENSG00000070961.15	ATP2B1	chr12_89448276_T_C_b38	rs10858862	0,0000036	0,16	Artery - Tibial
ENSG00000070961.15	ATP2B1	chr12_89451080_T_TG_b38	rs11461256	0,0000036	0,16	Artery - Tibial
ENSG00000070961.15	ATP2B1	chr12_89452453_C_T_b38	rs10858864	0,0000036	0,16	Artery - Tibial
ENSG00000070961.15	ATP2B1	chr12_89452699_G_A_b38	rs10858865	0,0000036	0,16	Artery - Tibial
ENSG00000070961.15	ATP2B1	chr12_89457389_C_T_b38	rs10858867	0,0000036	0,16	Artery - Tibial
ENSG00000070961.15	ATP2B1	chr12_89463834_C_T_b38	rs10858869	0,0000036	0,16	Artery - Tibial
ENSG00000070961.15	ATP2B1	chr12_89466542_C_A_b38	rs3803128	0,0000036	0,16	Artery - Tibial
ENSG00000070961.15	ATP2B1	chr12_89467752_T_C_b38	rs10506972	0,0000036	0,16	Artery - Tibial
ENSG00000070961.15	ATP2B1	chr12_89468253_G_T_b38	rs12228177	0,0000036	0,16	Artery - Tibial
ENSG00000070961.15	ATP2B1	chr12_89477836_G_A_b38	rs10858871	0,0000036	0,16	Artery - Tibial
ENSG00000070961.15	ATP2B1	chr12_89479155_C_T_b38	rs10858872	0,0000036	0,16	Artery - Tibial
ENSG00000070961.15	ATP2B1	chr12_89483146_C_T_b38	rs112511312	0,0000036	0,16	Artery - Tibial
ENSG00000070961.15	ATP2B1	chr12_89484166_A_G_b38	rs11105299	0,0000036	0,16	Artery - Tibial
ENSG00000070961.15	ATP2B1	chr12_89487787_C_G_b38	rs4842488	0,0000036	0,16	Artery - Tibial
ENSG00000070961.15	ATP2B1	chr12_89490501_C_T_b38	rs10858877	0,0000036	0,16	Artery - Tibial
ENSG00000070961.15	ATP2B1	chr12_89490517_G_A_b38	rs10858878	0,0000036	0,16	Artery - Tibial
ENSG00000070961.15	ATP2B1	chr12_89492146_C_A_b38	rs6538189	0,0000036	0,16	Artery - Tibial
ENSG00000070961.15	ATP2B1	chr12_89492165_T_C_b38	rs6538190	0,0000036	0,16	Artery - Tibial
ENSG00000070961.15	ATP2B1	chr12_89492801_T_A_b38	rs10858879	0,0000036	0,16	Artery - Tibial
ENSG00000070961.15	ATP2B1	chr12_89493872_T_C_b38	rs1008764	0,0000036	0,16	Artery - Tibial
ENSG00000070961.15	ATP2B1	chr12_89503120_C_T_b38	rs10858885	0,0000036	0,16	Artery - Tibial
ENSG00000070961.15	ATP2B1	chr12_89503384_C_G_b38	rs10858886	0,0000036	0,16	Artery - Tibial
ENSG00000070961.15	ATP2B1	chr12_89503557_C_T_b38	rs11105305	0,0000036	0,16	Artery - Tibial
ENSG00000070961.15	ATP2B1	chr12_89503611_C_T_b38	rs11105306	0,0000036	0,16	Artery - Tibial
ENSG00000070961.15	ATP2B1	chr12_89508827_G_A_b38	rs10858889	0,0000036	0,16	Artery - Tibial
ENSG00000070961.15	ATP2B1	chr12_89513389_T_C_b38	rs10858890	0,0000036	0,16	Artery - Tibial
ENSG00000070961.15	ATP2B1	chr12_89516073_T_C_b38	rs11105309	0,0000036	0,16	Artery - Tibial
ENSG00000070961.15	ATP2B1	chr12_89519642_C_T_b38	rs1054807	0,0000036	0,16	Artery - Tibial
ENSG00000070961.15	ATP2B1	chr12_89520484_T_C_b38	rs10858894	0,0000036	0,16	Artery - Tibial
ENSG00000070961.15	ATP2B1	chr12_89529599_A_T_b38	rs11105313	0,0000036	0,16	Artery - Tibial
ENSG00000070961.15	ATP2B1	chr12_90056289_A_G_b38	rs12227543	0,0000036	-0,15	Esophagus - Mucosa
ENSG00000070961.15	ATP2B1	chr12_89524900_C_A_b38	rs11105310	0,0000037	0,16	Artery - Tibial
ENSG00000070961.15	ATP2B1	chr12_89525791_C_T_b38	rs10777184	0,0000037	0,16	Artery - Tibial
ENSG00000070961.15	ATP2B1	chr12_89526407_C_T_b38	rs10858896	0,0000037	0,16	Artery - Tibial
ENSG00000070961.15	ATP2B1	chr12_89527500_G_A_b38	rs10777186	0,0000037	0,16	Artery - Tibial
ENSG00000070961.15	ATP2B1	chr12_90052157_T_C_b38	rs4842710	0,0000037	-0,15	Esophagus - Mucosa
ENSG00000070961.15	ATP2B1	chr12_90057274_A_G_b38	rs10858947	0,0000037	-0,15	Esophagus - Mucosa
ENSG00000070961.15	ATP2B1	chr12_90068211_G_A_b38	rs7139279	0,0000037	-0,15	Esophagus - Mucosa
ENSG00000070961.15	ATP2B1	chr12_90077187_C_T_b38	rs7488228	0,0000037	-0,15	Esophagus - Mucosa
ENSG00000070961.15	ATP2B1	chr12_90083730_C_T_b38	rs4842505	0,0000037	-0,15	Esophagus - Mucosa
ENSG00000070961.15	ATP2B1	chr12_90083900_C_T_b38	rs7306280	0,0000037	-0,15	Esophagus - Mucosa
ENSG00000070961.15	ATP2B1	chr12_89996947_A_G_b38	rs7953742	0,0000038	-0,15	Esophagus - Mucosa
ENSG00000070961.15	ATP2B1	chr12_89996827_CA_C_b38	rs112780917	0,0000039	-0,15	Esophagus - Mucosa
ENSG00000070961.15	ATP2B1	chr12_90007154_C_CA_b38	rs11396475	0,0000039	-0,15	Esophagus - Mucosa
ENSG00000070961.15	ATP2B1	chr12_90018978_A_C_b38	rs4842705	0,0000039	-0,15	Esophagus - Mucosa
ENSG00000070961.15	ATP2B1	chr12_89531304_C_A_b38	rs10858900	0,0000041	0,16	Artery - Tibial

ENSG00000070961.15	ATP2B1	chr12_89536622_A_G_b38	rs4842661	0,0000047	0,17	Artery - Tibial
ENSG00000070961.15	ATP2B1	chr12_89539933_C_T_b38	rs3825330	0,000005	0,16	Artery - Tibial
ENSG00000070961.15	ATP2B1	chr12_89541610_C_T_b38	rs10858909	0,000005	0,16	Artery - Tibial
ENSG00000070961.15	ATP2B1	chr12_89544813_G_A_b38	rs11105325	0,000005	0,16	Artery - Tibial
ENSG00000070961.15	ATP2B1	chr12_90050964_A_G_b38	rs10777222	0,000005	-0,15	Esophagus - Mucosa
ENSG00000070961.15	ATP2B1	chr12_89540154_G_A_b38	rs3741899	0,0000051	0,16	Artery - Tibial
ENSG00000070961.15	ATP2B1	chr12_90389431_C_T_b38	rs1228496	0,0000052	0,49	Brain - Substantia nigra
ENSG00000070961.15	ATP2B1	chr12_89528334_A_G_b38	rs10858898	0,0000052	0,16	Artery - Tibial
ENSG00000070961.15	ATP2B1	chr12_89544253_G_A_b38	rs4842664	0,0000052	0,16	Artery - Tibial
ENSG00000070961.15	ATP2B1	chr12_90510721_G_T_b38	rs12578110	0,0000055	0,46	Brain - Substantia nigra
ENSG00000070961.15	ATP2B1	chr12_89532881_G_A_b38	rs11105318	0,0000056	0,16	Artery - Tibial
ENSG00000070961.15	ATP2B1	chr12_89931928_T_C_b38	rs7970120	0,0000063	-0,21	Artery - Tibial
ENSG00000070961.15	ATP2B1	chr12_89432335_C_G_b38	rs11105286	0,0000064	0,15	Artery - Tibial
ENSG00000070961.15	ATP2B1	chr12_90386667_C_T_b38	rs6538234	0,0000083	0,51	Brain - Substantia nigra
ENSG00000070961.15	ATP2B1	chr12_90388244_T_A_b38	rs7979392	0,0000083	0,51	Brain - Substantia nigra
ENSG00000070961.15	ATP2B1	chr12_90390285_G_C_b38	rs2043213	0,0000083	0,51	Brain - Substantia nigra
ENSG00000070961.15	ATP2B1	chr12_90398938_C_T_b38	rs7955801	0,0000083	0,51	Brain - Substantia nigra
ENSG00000070961.15	ATP2B1	chr12_90063091_G_A_b38	rs6538220	0,0000084	-0,15	Esophagus - Mucosa
ENSG00000070961.15	ATP2B1	chr12_90516738_A_G_b38	rs1347015	0,0000088	0,47	Brain - Substantia nigra
ENSG00000070961.15	ATP2B1	chr12_90523189_C_T_b38	rs1579534	0,0000088	0,47	Brain - Substantia nigra
ENSG00000070961.15	ATP2B1	chr12_90510105_C_T_b38	rs10858992	0,0000088	0,46	Brain - Substantia nigra
ENSG00000070961.15	ATP2B1	chr12_90519613_A_T_b38	rs61655253	0,0000088	0,46	Brain - Substantia nigra
ENSG00000070961.15	ATP2B1	chr12_90528232_A_G_b38	rs4274255	0,0000088	0,46	Brain - Substantia nigra
ENSG00000070961.15	ATP2B1	chr12_90528837_C_T_b38	rs11105663	0,0000088	0,46	Brain - Substantia nigra
ENSG00000070961.15	ATP2B1	chr12_90532822_TTCTTAAGGACAA_T_b38	rs6144807	0,0000088	0,46	Brain - Substantia nigra
ENSG00000070961.15	ATP2B1	chr12_90533124_TA_T_b38	rs36032096	0,0000088	0,46	Brain - Substantia nigra
ENSG00000070961.15	ATP2B1	chr12_90535560_T_C_b38	rs12580833	0,0000088	0,46	Brain - Substantia nigra
ENSG00000070961.15	ATP2B1	chr12_90543285_C_T_b38	rs11105668	0,0000088	0,46	Brain - Substantia nigra
ENSG00000070961.15	ATP2B1	chr12_89780365_G_T_b38	rs116990534	0,0000092	0,35	Artery - Tibial
ENSG00000070961.15	ATP2B1	chr12_89719404_G_A_b38	rs4842679	0,0000094	-0,15	Artery - Tibial
ENSG00000070961.15	ATP2B1	chr12_89504370_G_A_b38	rs138808993	0,0000095	0,65	Brain - Anterior cingulate cortex (BA24)
ENSG00000070961.15	ATP2B1	chr12_89538657_G_A_b38	rs143285018	0,0000095	0,65	Brain - Anterior cingulate cortex (BA24)
ENSG00000070961.15	ATP2B1	chr12_90129715_G_A_b38	rs59313167	0,0000097	-0,15	Esophagus - Mucosa
ENSG00000070961.15	ATP2B1	chr12_90134908_C_T_b38	rs7970711	0,0000097	-0,15	Esophagus - Mucosa
ENSG00000070961.15	ATP2B1	chr12_90136962_G_A_b38	rs7979050	0,0000097	-0,15	Esophagus - Mucosa
ENSG00000070961.15	ATP2B1	chr12_89959608_C_T_b38	rs11105474	0,00001	-0,14	Esophagus - Mucosa
ENSG00000070961.15	ATP2B1	chr12_89622750_A_G_b38	rs147471906	0,000011	0,59	Brain - Anterior cingulate cortex (BA24)
ENSG00000070961.15	ATP2B1	chr12_89643621_G_A_b38	rs149731397	0,000011	0,59	Brain - Anterior cingulate cortex (BA24)
ENSG00000070961.15	ATP2B1	chr12_90394122_TTTC_T_b38	rs10543504	0,000011	0,52	Brain - Substantia nigra
ENSG00000070961.15	ATP2B1	chr12_90385245_G_A_b38	rs1594565	0,000011	0,5	Brain - Substantia nigra
ENSG00000070961.15	ATP2B1	chr12_90088859_T_C_b38	rs11105501	0,000011	-0,15	Esophagus - Mucosa
ENSG00000070961.15	ATP2B1	chr12_90089663_G_A_b38	rs11105503	0,000011	-0,15	Esophagus - Mucosa
ENSG00000070961.15	ATP2B1	chr12_90089938_C_T_b38	rs11105504	0,000011	-0,15	Esophagus - Mucosa
ENSG00000070961.15	ATP2B1	chr12_89926004_G_A_b38	rs111342256	0,000012	0,65	Artery - Tibial
ENSG00000070961.15	ATP2B1	chr12_90195090_A_G_b38	rs10858967	0,000012	-0,34	Cells - EBV-transformed lymphocytes
ENSG00000070961.15	ATP2B1	chr12_90407150_G_A_b38	rs7969733	0,000013	0,34	Brain - Spinal cord (cervical c-1)
ENSG00000070961.15	ATP2B1	chr12_90411424_C_G_b38	rs10777244	0,000013	0,34	Brain - Spinal cord (cervical c-1)
ENSG00000070961.15	ATP2B1	chr12_90411690_G_A_b38	rs7958205	0,000013	0,34	Brain - Spinal cord (cervical c-1)
ENSG00000070961.15	ATP2B1	chr12_90411780_G_A_b38	rs7958308	0,000013	0,34	Brain - Spinal cord (cervical c-1)
ENSG00000070961.15	ATP2B1	chr12_90414020_G_T_b38	rs7966452	0,000013	0,34	Brain - Spinal cord (cervical c-1)
ENSG00000070961.15	ATP2B1	chr12_90414588_CT_C_b38	rs35829324	0,000013	0,34	Brain - Spinal cord (cervical c-1)
ENSG00000070961.15	ATP2B1	chr12_90416671_A_G_b38	rs2897862	0,000013	0,34	Brain - Spinal cord (cervical c-1)
ENSG00000070961.15	ATP2B1	chr12_90416672_A_T_b38	rs2408160	0,000013	0,34	Brain - Spinal cord (cervical c-1)
ENSG00000070961.15	ATP2B1	chr12_90422109_C_T_b38	rs7315821	0,000013	0,34	Brain - Spinal cord (cervical c-1)
ENSG00000070961.15	ATP2B1	chr12_90425535_T_A_b38	rs6538235	0,000013	0,34	Brain - Spinal cord (cervical c-1)
ENSG00000070961.15	ATP2B1	chr12_90427858_G_A_b38	rs10777245	0,000013	0,34	Brain - Spinal cord (cervical c-1)

ENSG00000070961.15	ATP2B1	chr12_90430064_C_G_b38	rs6538236	0,000013	0,34	Brain - Spinal cord (cervical c-1)
ENSG00000070961.15	ATP2B1	chr12_90436231_T_C_b38	rs1835768	0,000013	0,34	Brain - Spinal cord (cervical c-1)
ENSG00000070961.15	ATP2B1	chr12_90436298_G_A_b38	rs1835767	0,000013	0,34	Brain - Spinal cord (cervical c-1)
ENSG00000070961.15	ATP2B1	chr12_90439201_G_A_b38	rs34234695	0,000013	0,34	Brain - Spinal cord (cervical c-1)
ENSG00000070961.15	ATP2B1	chr12_90439855_C_T_b38	rs11105617	0,000013	0,34	Brain - Spinal cord (cervical c-1)
ENSG00000070961.15	ATP2B1	chr12_90443686_G_A_b38	rs66955893	0,000013	0,34	Brain - Spinal cord (cervical c-1)
ENSG00000070961.15	ATP2B1	chr12_90451488_G_A_b38	rs11105622	0,000013	0,34	Brain - Spinal cord (cervical c-1)
ENSG00000070961.15	ATP2B1	chr12_90452503_C_T_b38	rs7960740	0,000013	0,34	Brain - Spinal cord (cervical c-1)
ENSG00000070961.15	ATP2B1	chr12_90457546_A_T_b38	rs11105623	0,000013	0,34	Brain - Spinal cord (cervical c-1)
ENSG00000070961.15	ATP2B1	chr12_90468635_G_A_b38	rs7954409	0,000013	0,34	Brain - Spinal cord (cervical c-1)
ENSG00000070961.15	ATP2B1	chr12_90474639_G_A_b38	rs1864782	0,000013	0,34	Brain - Spinal cord (cervical c-1)
ENSG00000070961.15	ATP2B1	chr12_90476135_C_T_b38	rs7298940	0,000013	0,34	Brain - Spinal cord (cervical c-1)
ENSG00000070961.15	ATP2B1	chr12_90478226_G_A_b38	rs12818545	0,000013	0,34	Brain - Spinal cord (cervical c-1)
ENSG00000070961.15	ATP2B1	chr12_90482472_AAC_A_b38	rs67668093	0,000013	0,34	Brain - Spinal cord (cervical c-1)
ENSG00000070961.15	ATP2B1	chr12_90484858_C_T_b38	rs7311599	0,000013	0,34	Brain - Spinal cord (cervical c-1)
ENSG00000070961.15	ATP2B1	chr12_90559065_T_A_b38	rs2520561	0,000013	0,31	Brain - Spinal cord (cervical c-1)
ENSG00000070961.15	ATP2B1	chr12_90391772_A_C_b38	rs7976895	0,000014	0,48	Brain - Substantia nigra
ENSG00000070961.15	ATP2B1	chr12_90392860_A_C_b38	rs2116497	0,000014	0,48	Brain - Substantia nigra
ENSG00000070961.15	ATP2B1	chr12_90393455_G_A_b38	rs12580873	0,000014	0,48	Brain - Substantia nigra
ENSG00000070961.15	ATP2B1	chr12_90552916_G_A_b38	rs2520567	0,000014	0,44	Brain - Substantia nigra
ENSG00000070961.15	ATP2B1	chr12_90001825_T_C_b38	rs17192565	0,000014	0,43	Artery - Tibial
ENSG00000070961.15	ATP2B1	chr12_90459042_T_A_b38	rs7132660	0,000014	0,34	Brain - Spinal cord (cervical c-1)
ENSG00000070961.15	ATP2B1	chr12_90106317_C_CAT_b38	rs148143712	0,000015	-0,15	Esophagus - Mucosa
ENSG00000070961.15	ATP2B1	chr12_90112140_G_A_b38	rs7966278	0,000015	-0,15	Esophagus - Mucosa
ENSG00000070961.15	ATP2B1	chr12_89556543_A_C_b38	rs7302816	0,000016	0,19	Artery - Aorta
ENSG00000070961.15	ATP2B1	chr12_89422110_G_A_b38	rs80277599	0,000017	1,4	Brain - Hypothalamus
ENSG00000070961.15	ATP2B1	chr12_89428758_A_G_b38	rs112370308	0,000017	1,4	Brain - Hypothalamus
ENSG00000070961.15	ATP2B1	chr12_89441431_T_C_b38	rs113025676	0,000017	1,4	Brain - Hypothalamus
ENSG00000070961.15	ATP2B1	chr12_89457067_C_T_b38	rs61435009	0,000017	1,4	Brain - Hypothalamus
ENSG00000070961.15	ATP2B1	chr12_89471149_A_G_b38	rs74754458	0,000017	1,4	Brain - Hypothalamus
ENSG00000070961.15	ATP2B1	chr12_89478671_T_C_b38	rs60772618	0,000017	1,4	Brain - Hypothalamus
ENSG00000070961.15	ATP2B1	chr12_89484136_C_G_b38	rs74543833	0,000017	1,4	Brain - Hypothalamus
ENSG00000070961.15	ATP2B1	chr12_89487042_C_G_b38	rs58117070	0,000017	1,4	Brain - Hypothalamus
ENSG00000070961.15	ATP2B1	chr12_89488169_C_T_b38	rs185300284	0,000017	1,4	Brain - Hypothalamus
ENSG00000070961.15	ATP2B1	chr12_89488792_C_T_b38	rs76672752	0,000017	1,4	Brain - Hypothalamus
ENSG00000070961.15	ATP2B1	chr12_89490053_G_T_b38	rs78332000	0,000017	1,4	Brain - Hypothalamus
ENSG00000070961.15	ATP2B1	chr12_90581088_T_TA_b38	rs143922775	0,000017	1,4	Brain - Hypothalamus
ENSG00000070961.15	ATP2B1	chr12_90583461_C_T_b38	rs17018043	0,000017	1,4	Brain - Hypothalamus
ENSG00000070961.15	ATP2B1	chr12_90586839_A_G_b38	rs73358180	0,000017	1,4	Brain - Hypothalamus
ENSG00000070961.15	ATP2B1	chr12_90589431_T_C_b38	rs73358183	0,000017	1,4	Brain - Hypothalamus
ENSG00000070961.15	ATP2B1	chr12_90593192_C_T_b38	rs73358194	0,000017	1,4	Brain - Hypothalamus
ENSG00000070961.15	ATP2B1	chr12_90594592_G_A_b38	rs2106528	0,000017	1,4	Brain - Hypothalamus
ENSG00000070961.15	ATP2B1	chr12_90599502_C_T_b38	rs76134788	0,000017	1,4	Brain - Hypothalamus
ENSG00000070961.15	ATP2B1	chr12_90602009_G_C_b38	rs73360109	0,000017	1,4	Brain - Hypothalamus
ENSG00000070961.15	ATP2B1	chr12_90602975_T_C_b38	rs73360112	0,000017	1,4	Brain - Hypothalamus
ENSG00000070961.15	ATP2B1	chr12_90603192_A_G_b38	rs17018088	0,000017	1,4	Brain - Hypothalamus
ENSG00000070961.15	ATP2B1	chr12_90606864_CAG_C_b38	rs34639687	0,000017	1,4	Brain - Hypothalamus
ENSG00000070961.15	ATP2B1	chr12_90608792_A_G_b38	rs7306918	0,000017	1,4	Brain - Hypothalamus
ENSG00000070961.15	ATP2B1	chr12_90609936_C_T_b38	rs73360125	0,000017	1,4	Brain - Hypothalamus
ENSG00000070961.15	ATP2B1	chr12_90614576_G_T_b38	rs73360133	0,000017	1,4	Brain - Hypothalamus
ENSG00000070961.15	ATP2B1	chr12_90626419_G_T_b38	rs11829105	0,000017	1,4	Brain - Hypothalamus
ENSG00000070961.15	ATP2B1	chr12_90626488_A_T_b38	rs11833692	0,000017	1,4	Brain - Hypothalamus
ENSG00000070961.15	ATP2B1	chr12_90628581_A_G_b38	rs73360170	0,000017	1,4	Brain - Hypothalamus
ENSG00000070961.15	ATP2B1	chr12_90646115_A_G_b38	rs11830515	0,000017	1,4	Brain - Hypothalamus
ENSG00000070961.15	ATP2B1	chr12_90650906_T_C_b38	rs11105723	0,000017	1,4	Brain - Hypothalamus
ENSG00000070961.15	ATP2B1	chr12_90661217_T_C_b38	rs11105730	0,000017	1,4	Brain - Hypothalamus

ENSG00000070961.15	ATP2B1	chr12_89525853_ACC_A_b38	rs67346689	0,000017	0,16	Artery - Tibial
ENSG00000070961.15	ATP2B1	chr12_90637101_TTG_T_b38	rs1491580271	0,000018	1,4	Brain - Hypothalamus
ENSG00000070961.15	ATP2B1	chr12_90136354_G_A_b38	rs143689254	0,000018	0,68	Brain - Anterior cingulate cortex (BA24)
ENSG00000070961.15	ATP2B1	chr12_90241276_A_G_b38	rs73196463	0,000018	0,62	Artery - Tibial
ENSG00000070961.15	ATP2B1	chr12_90554733_G_A_b38	rs2520565	0,000018	0,44	Brain - Substantia nigra
ENSG00000070961.15	ATP2B1	chr12_90556726_A_G_b38	rs2520563	0,000018	0,44	Brain - Substantia nigra
ENSG00000070961.15	ATP2B1	chr12_90557663_C_T_b38	rs2520562	0,000018	0,44	Brain - Substantia nigra
ENSG00000070961.15	ATP2B1	chr12_90558072_T_C_b38	rs2723917	0,000018	0,44	Brain - Substantia nigra
ENSG00000070961.15	ATP2B1	chr12_89569279_G_A_b38	rs372643302	0,000018	0,17	Artery - Tibial
ENSG00000070961.15	ATP2B1	chr12_89950918_A_G_b38	rs7294671	0,000018	-0,13	Esophagus - Mucosa
ENSG00000070961.15	ATP2B1	chr12_89956338_C_G_b38	rs11105473	0,000018	-0,14	Esophagus - Mucosa
ENSG00000070961.15	ATP2B1	chr12_89957460_T_G_b38	rs7132526	0,000018	-0,14	Esophagus - Mucosa
ENSG00000070961.15	ATP2B1	chr12_89704243_G_A_b38	rs61926784	0,000019	0,19	Brain - Frontal Cortex (BA9)
ENSG00000070961.15	ATP2B1	chr12_90326873_G_T_b38	rs825954	0,000023	-0,15	Esophagus - Muscularis
ENSG00000070961.15	ATP2B1	chr12_90329398_C_T_b38	rs10858974	0,000024	-0,13	Thyroid
ENSG00000070961.15	ATP2B1	chr12_90299326_G_A_b38	rs11105563	0,000024	-0,35	Cells - EBV-transformed lymphocytes
ENSG00000070961.15	ATP2B1	chr12_89876539_G_A_b38	rs74851539	0,000025	0,41	Artery - Tibial
ENSG00000070961.15	ATP2B1	chr12_89940862_G_A_b38	rs12368259	0,000027	0,22	Esophagus - Mucosa
ENSG00000070961.15	ATP2B1	chr12_90299488_G_C_b38	rs10506984	0,000027	-0,34	Cells - EBV-transformed lymphocytes
ENSG00000070961.15	ATP2B1	chr12_90303890_G_C_b38	rs2056699	0,000027	-0,34	Cells - EBV-transformed lymphocytes
ENSG00000070961.15	ATP2B1	chr12_90306554_G_A_b38	rs1587440	0,000027	-0,34	Cells - EBV-transformed lymphocytes
ENSG00000070961.15	ATP2B1	chr12_90311197_C_T_b38	rs10858971	0,000027	-0,34	Cells - EBV-transformed lymphocytes
ENSG00000070961.15	ATP2B1	chr12_90631370_A_T_b38	rs11105706	0,000028	-0,28	Colon - Transverse
ENSG00000070961.15	ATP2B1	chr12_90631458_A_G_b38	rs11105707	0,000028	-0,28	Colon - Transverse
ENSG00000070961.15	ATP2B1	chr12_90632378_G_A_b38	rs11105709	0,000028	-0,28	Colon - Transverse
ENSG00000070961.15	ATP2B1	chr12_90636015_C_A_b38	rs11105712	0,000028	-0,28	Colon - Transverse
ENSG00000070961.15	ATP2B1	chr12_90638508_T_A_b38	rs112784216	0,000028	-0,28	Colon - Transverse
ENSG00000070961.15	ATP2B1	chr12_90451210_G_A_b38	rs1148676	0,000029	0,3	Brain - Spinal cord (cervical c-1)
ENSG00000070961.15	ATP2B1	chr12_89973104_A_G_b38	rs10777215	0,000029	-0,13	Esophagus - Mucosa
ENSG00000070961.15	ATP2B1	chr12_89999628_A_ATTC_b38	rs10671258	0,000029	-0,13	Esophagus - Mucosa
ENSG00000070961.15	ATP2B1	chr12_90000940_A_G_b38	rs4406875	0,000029	-0,13	Esophagus - Mucosa
ENSG00000070961.15	ATP2B1	chr12_90005097_T_A_b38	rs4240746	0,000029	-0,13	Esophagus - Mucosa
ENSG00000070961.15	ATP2B1	chr12_89559134_C_T_b38	rs112417857	0,000031	0,54	Artery - Tibial
ENSG00000070961.15	ATP2B1	chr12_89394010_T_C_b38	rs11105273	0,000031	0,18	Artery - Tibial
ENSG00000070961.15	ATP2B1	chr12_89975574_A_G_b38	rs7303123	0,000031	-0,13	Esophagus - Mucosa
ENSG00000070961.15	ATP2B1	chr12_89976239_C_T_b38	rs10777216	0,000031	-0,13	Esophagus - Mucosa
ENSG00000070961.15	ATP2B1	chr12_89957885_A_G_b38	rs4842700	0,000032	-0,18	Artery - Tibial
ENSG00000070961.15	ATP2B1	chr12_90287401_C_T_b38	rs11105559	0,000032	-0,34	Cells - EBV-transformed lymphocytes
ENSG00000070961.15	ATP2B1	chr12_90295570_G_A_b38	rs2165035	0,000032	-0,34	Cells - EBV-transformed lymphocytes
ENSG00000070961.15	ATP2B1	chr12_90273379_C_A_b38	rs12320693	0,000033	-0,37	Cells - EBV-transformed lymphocytes
ENSG00000070961.15	ATP2B1	chr12_89995520_A_C_b38	rs6538214	0,000034	-0,13	Esophagus - Mucosa
ENSG00000070961.15	ATP2B1	chr12_90007782_T_TATAAA_b38	rs57291885	0,000034	-0,13	Esophagus - Mucosa
ENSG00000070961.15	ATP2B1	chr12_90008924_A_C_b38	rs4842504	0,000034	-0,13	Esophagus - Mucosa
ENSG00000070961.15	ATP2B1	chr12_90018772_T_C_b38	rs10858942	0,000034	-0,13	Esophagus - Mucosa
ENSG00000070961.15	ATP2B1	chr12_90061403_T_C_b38	rs7303035	0,000037	-0,13	Esophagus - Mucosa
ENSG00000070961.15	ATP2B1	chr12_89042639_G_A_b38	rs76671707	0,000037	-0,57	Lung
ENSG00000070961.15	ATP2B1	chr12_90266564_G_A_b38	rs1347846	0,000039	-0,33	Cells - EBV-transformed lymphocytes
ENSG00000070961.15	ATP2B1	chr12_90259816_T_C_b38	rs4381424	0,000039	-0,34	Cells - EBV-transformed lymphocytes
ENSG00000070961.15	ATP2B1	chr12_90303378_AT_A_b38	rs141511768	0,000039	-0,36	Cells - EBV-transformed lymphocytes
ENSG00000070961.15	ATP2B1	chr12_90275487_CAA_C_b38	rs3042884	0,000041	-0,35	Cells - EBV-transformed lymphocytes
ENSG00000070961.15	ATP2B1	chr12_90328476_T_C_b38	rs1405527	0,000042	-0,13	Thyroid
ENSG00000070961.15	ATP2B1	chr12_90330109_G_A_b38	rs12818395	0,000042	-0,13	Thyroid
ENSG00000070961.15	ATP2B1	chr12_90150155_C_T_b38	rs7135166	0,000042	-0,14	Esophagus - Mucosa
ENSG00000070961.15	ATP2B1	chr12_89709017_C_A_b38	rs12369944	0,000044	0,18	Brain - Frontal Cortex (BA9)
ENSG00000070961.15	ATP2B1	chr12_89966286_A_G_b38	rs10745520	0,000044	-0,13	Esophagus - Mucosa
ENSG00000070961.15	ATP2B1	chr12_89702020_T_A_b38	rs11105380	0,000045	-0,14	Artery - Tibial
ENSG00000070961.15	ATP2B1	chr12_90153750_C_G_b38	rs2118803	0,000047	-0,14	Esophagus - Mucosa

ENSG00000070961.15	ATP2B1	chr12_90568565_A_G_b38	rs35520023	0,000048	0,13	Adipose - Subcutaneous
ENSG00000070961.15	ATP2B1	chr12_90629207_G_A_b38	rs11105704	0,000048	-0,27	Colon - Transverse
ENSG00000070961.15	ATP2B1	chr12_89666809_G_A_b38	rs17249754	0,000051	0,19	Artery - Aorta
ENSG00000070961.15	ATP2B1	chr12_89668599_G_A_b38	rs6538195	0,000051	0,19	Artery - Aorta
ENSG00000070961.15	ATP2B1	chr12_89678299_G_A_b38	rs73437358	0,000051	0,19	Artery - Aorta
ENSG00000070961.15	ATP2B1	chr12_89913265_C_T_b38	rs7972820	0,000053	-0,13	Esophagus - Mucosa
ENSG00000070961.15	ATP2B1	chr12_89784106_A_T_b38	rs113133867	0,000054	0,55	Artery - Tibial
ENSG00000070961.15	ATP2B1	chr12_89632685_G_A_b38	rs11105352	0,000054	0,19	Artery - Aorta
ENSG00000070961.15	ATP2B1	chr12_89632686_C_A_b38	rs11105353	0,000054	0,19	Artery - Aorta
ENSG00000070961.15	ATP2B1	chr12_89632746_A_G_b38	rs11105354	0,000054	0,19	Artery - Aorta
ENSG00000070961.15	ATP2B1	chr12_89656726_A_G_b38	rs12579302	0,000054	0,19	Artery - Aorta
ENSG00000070961.15	ATP2B1	chr12_89665065_G_A_b38	rs111478946	0,000054	0,19	Artery - Aorta
ENSG00000070961.15	ATP2B1	chr12_89675499_T_G_b38	rs11105364	0,000054	0,19	Artery - Aorta
ENSG00000070961.15	ATP2B1	chr12_90158457_G_GT_b38	rs79468373	0,000054	-0,14	Esophagus - Mucosa
ENSG00000070961.15	ATP2B1	chr12_89909790_G_C_b38	rs7957657	0,000057	-0,13	Esophagus - Mucosa
ENSG00000070961.15	ATP2B1	chr12_90328476_T_C_b38	rs1405527	0,000057	-0,15	Esophagus - Muscularis
ENSG00000070961.15	ATP2B1	chr12_90329398_C_T_b38	rs10858974	0,000057	-0,15	Esophagus - Muscularis
ENSG00000070961.15	ATP2B1	chr12_90330109_G_A_b38	rs12818395	0,000057	-0,15	Esophagus - Muscularis
ENSG00000070961.15	ATP2B1	chr12_90026116_GT_G_b38	rs11324040	0,000058	-0,13	Esophagus - Mucosa
ENSG00000070961.15	ATP2B1	chr12_89629273_T_C_b38	rs116858620	0,00006	0,43	Lung
ENSG00000070961.15	ATP2B1	chr12_90035251_A_G_b38	rs7311631	0,00006	-0,13	Esophagus - Mucosa
ENSG00000070961.15	ATP2B1	chr12_89879521_G_A_b38	rs2553100	0,00006	-0,13	Esophagus - Mucosa
ENSG00000070961.15	ATP2B1	chr12_89944741_G_A_b38	rs4842698	0,000062	-0,18	Artery - Tibial
ENSG00000070961.15	ATP2B1	chr12_89680664_G_C_b38	rs11105368	0,000063	0,19	Artery - Aorta
ENSG00000070961.15	ATP2B1	chr12_90577787_T_C_b38	rs11105685	0,000063	0,13	Adipose - Subcutaneous
ENSG00000070961.15	ATP2B1	chr12_89681466_C_T_b38	rs4842675	0,000065	0,19	Artery - Aorta
ENSG00000070961.15	ATP2B1	chr12_89619312_T_C_b38	rs2681492	0,000066	0,19	Artery - Aorta
ENSG00000070961.15	ATP2B1	chr12_89615182_A_G_b38	rs2681472	0,000066	0,19	Artery - Aorta
ENSG00000070961.15	ATP2B1	chr12_89956222_G_A_b38	rs10777213	0,000066	-0,13	Esophagus - Mucosa
ENSG00000070961.15	ATP2B1	chr12_89958659_C_T_b38	rs7306228	0,000066	-0,13	Esophagus - Mucosa
ENSG00000070961.15	ATP2B1	chr12_90039790_A_G_b38	rs6538218	0,000066	-0,13	Esophagus - Mucosa
ENSG00000070961.15	ATP2B1	chr12_89912752_T_C_b38	rs7135197	0,000067	-0,13	Esophagus - Mucosa
ENSG00000070961.15	ATP2B1	chr12_89915156_G_A_b38	rs7959788	0,000067	-0,13	Esophagus - Mucosa
ENSG00000070961.15	ATP2B1	chr12_90152680_A_G_b38	rs10745525	0,000068	-0,18	Artery - Tibial
ENSG00000070961.15	ATP2B1	chr12_89837533_A_G_b38	rs2722224	0,000079	-0,13	Esophagus - Mucosa
ENSG00000070961.15	ATP2B1	chr12_89696964_C_T_b38	rs11105378	0,00008	0,19	Artery - Aorta
ENSG00000070961.15	ATP2B1	chr12_89695013_G_A_b38	rs10858917	0,00008	-0,17	Artery - Aorta
ENSG00000070961.15	ATP2B1	chr12_90050278_C_T_b38	rs55846104	0,000081	-0,19	Esophagus - Mucosa
ENSG00000070961.15	ATP2B1	chr12_90147932_C_T_b38	rs4608145	0,000083	-0,13	Lung
ENSG00000070961.15	ATP2B1	chr12_90087601_T_C_b38	rs7316395	0,000087	-0,13	Lung
ENSG00000070961.15	ATP2B1	chr12_90087981_A_G_b38	rs7300611	0,000087	-0,13	Lung
ENSG00000070961.15	ATP2B1	chr12_90089145_A_G_b38	rs10735278	0,000087	-0,13	Lung
ENSG00000070961.15	ATP2B1	chr12_90089249_A_T_b38	rs10735279	0,000087	-0,13	Lung
ENSG00000070961.15	ATP2B1	chr12_90089939_T_G_b38	rs10858950	0,000087	-0,13	Lung
ENSG00000070961.15	ATP2B1	chr12_90090107_T_C_b38	rs11105505	0,000087	-0,13	Lung
ENSG00000070961.15	ATP2B1	chr12_89966684_C_T_b38	rs4584633	0,00009	-0,17	Artery - Tibial
ENSG00000070961.15	ATP2B1	chr12_89921101_C_G_b38	rs4842693	0,000093	-0,13	Esophagus - Mucosa
ENSG00000070961.15	ATP2B1	chr12_90133970_C_T_b38	rs11105516	0,000094	-0,13	Lung
ENSG00000070961.15	ATP2B1	chr12_90022580_A_C_b38	rs10777219	0,000094	-0,14	Artery - Tibial
ENSG00000070961.15	ATP2B1	chr12_90024345_G_A_b38	rs10858943	0,000094	-0,14	Artery - Tibial
ENSG00000070961.15	ATP2B1	chr12_90157130_AT_A_b38	rs34734819	0,000099	-0,13	Esophagus - Mucosa
ENSG00000070961.15	ATP2B1	chr12_89694803_T_A_b38	rs11105375	0,0001	0,18	Artery - Aorta
ENSG00000070961.15	ATP2B1	chr12_89697090_A_G_b38	rs12230074	0,0001	0,18	Artery - Aorta
ENSG00000070961.15	ATP2B1	chr12_90148406_A_C_b38	rs1438984	0,0001	-0,13	Lung
ENSG00000070961.15	ATP2B1	chr12_90148426_G_A_b38	rs1438983	0,0001	-0,13	Lung
ENSG00000070961.15	ATP2B1	chr12_90042305_C_T_b38	rs7975953	0,0001	-0,14	Artery - Tibial
ENSG00000070961.15	ATP2B1	chr12_90042794_T_C_b38	rs7963304	0,0001	-0,14	Artery - Tibial
ENSG00000070961.15	ATP2B1	chr12_90110385_A_C_b38	rs10858955	0,00011	-0,13	Lung
ENSG00000070961.15	ATP2B1	chr12_90111572_G_A_b38	rs10745523	0,00011	-0,13	Lung
ENSG00000070961.15	ATP2B1	chr12_90114532_G_T_b38	rs10777224	0,00011	-0,13	Lung
ENSG00000070961.15	ATP2B1	chr12_90120301_C_G_b38	rs10858956	0,00011	-0,13	Lung
ENSG00000070961.15	ATP2B1	chr12_89336143_A_C_b38	rs10161308	0,00012	0,22	Artery - Tibial
ENSG00000070961.15	ATP2B1	chr12_89660842_T_C_b38	rs73437338	0,00012	0,19	Artery - Aorta
ENSG00000070961.15	ATP2B1	chr12_89591515_AAAGT_A_b38	rs10580742	0,00012	0,19	Artery - Aorta
ENSG00000070961.15	ATP2B1	chr12_89677042_CAAAG_C_b38	rs143087380	0,00012	-0,11	Thyroid
ENSG00000070961.15	ATP2B1	chr12_90099780_G_A_b38	rs7486390	0,00012	-0,13	Lung
ENSG00000070961.15	ATP2B1	chr12_90100728_G_C_b38	rs7303398	0,00012	-0,13	Lung
ENSG00000070961.15	ATP2B1	chr12_89694991_G_A_b38	rs11105376	0,00013	0,19	Artery - Aorta
ENSG00000070961.15	ATP2B1	chr12_90117803_G_C_b38	rs7486630	0,00013	-0,13	Lung
ENSG00000070961.15	ATP2B1	chr12_90119438_T_A_b38	rs7139353	0,00013	-0,13	Lung
ENSG00000070961.15	ATP2B1	chr12_90121102_T_C_b38	rs10777226	0,00013	-0,13	Lung
ENSG00000070961.15	ATP2B1	chr12_90122191_G_C_b38	rs6538227	0,00013	-0,13	Lung

ENSG00000070961.15	ATP2B1	chr12_90123327_G_T_b38	rs10858957	0,00013	-0,13	Lung
ENSG00000070961.15	ATP2B1	chr12_90123338_T_C_b38	rs10858958	0,00013	-0,13	Lung
ENSG00000070961.15	ATP2B1	chr12_90124075_G_A_b38	rs7315166	0,00013	-0,13	Lung
ENSG00000070961.15	ATP2B1	chr12_90124724_G_A_b38	rs4842713	0,00013	-0,13	Lung
ENSG00000070961.15	ATP2B1	chr12_90126369_C_T_b38	rs7138547	0,00013	-0,13	Lung
ENSG00000070961.15	ATP2B1	chr12_90127423_A_G_b38	rs10777227	0,00013	-0,13	Lung
ENSG00000070961.15	ATP2B1	chr12_90127443_A_G_b38	rs10777228	0,00013	-0,13	Lung
ENSG00000070961.15	ATP2B1	chr12_90128789_G_A_b38	rs12303180	0,00013	-0,13	Lung
ENSG00000070961.15	ATP2B1	chr12_90129235_T_C_b38	rs4842714	0,00013	-0,13	Lung
ENSG00000070961.15	ATP2B1	chr12_90130357_GCTC_G_b38	rs35647353	0,00013	-0,13	Lung
ENSG00000070961.15	ATP2B1	chr12_90132074_G_T_b38	rs10858961	0,00013	-0,13	Lung
ENSG00000070961.15	ATP2B1	chr12_90133198_A_AG_b38	rs79553876	0,00013	-0,13	Lung
ENSG00000070961.15	ATP2B1	chr12_90134620_G_C_b38	rs6538228	0,00013	-0,13	Lung
ENSG00000070961.15	ATP2B1	chr12_90137321_T_C_b38	rs1595599	0,00013	-0,13	Lung
ENSG00000070961.15	ATP2B1	chr12_90137388_G_A_b38	rs1595598	0,00013	-0,13	Lung
ENSG00000070961.15	ATP2B1	chr12_90137720_T_G_b38	rs7304226	0,00013	-0,13	Lung
ENSG00000070961.15	ATP2B1	chr12_90139086_C_T_b38	rs7295818	0,00013	-0,13	Lung
ENSG00000070961.15	ATP2B1	chr12_90141861_G_T_b38	rs1438986	0,00013	-0,13	Lung
ENSG00000070961.15	ATP2B1	chr12_90142076_T_G_b38	rs1438985	0,00013	-0,13	Lung
ENSG00000070961.15	ATP2B1	chr12_90142537_C_A_b38	rs1470031	0,00013	-0,13	Lung
ENSG00000070961.15	ATP2B1	chr12_90142786_G_A_b38	rs1470030	0,00013	-0,13	Lung
ENSG00000070961.15	ATP2B1	chr12_90144066_A_C_b38	rs10745524	0,00013	-0,13	Lung
ENSG00000070961.15	ATP2B1	chr12_90144234_A_G_b38	rs1470029	0,00013	-0,13	Lung
ENSG00000070961.15	ATP2B1	chr12_90144899_A_T_b38	rs4842715	0,00013	-0,13	Lung
ENSG00000070961.15	ATP2B1	chr12_90145400_C_A_b38	rs4842716	0,00013	-0,13	Lung
ENSG00000070961.15	ATP2B1	chr12_90145401_C_T_b38	rs4842717	0,00013	-0,13	Lung
ENSG00000070961.15	ATP2B1	chr12_90145987_C_A_b38	rs1344962	0,00013	-0,13	Lung
ENSG00000070961.15	ATP2B1	chr12_90146644_T_C_b38	rs11105517	0,00013	-0,13	Lung
ENSG00000070961.15	ATP2B1	chr12_90147279_C_G_b38	rs1344961	0,00013	-0,13	Lung
ENSG00000070961.15	ATP2B1	chr12_90147385_C_G_b38	rs2218085	0,00013	-0,13	Lung
ENSG00000070961.15	ATP2B1	chr12_90147644_A_C_b38	rs2196783	0,00013	-0,13	Lung
ENSG00000070961.15	ATP2B1	chr12_90002703_T_C_b38	rs7964216	0,00013	-0,14	Artery - Tibial
ENSG00000070961.15	ATP2B1	chr12_90004485_T_C_b38	rs10777218	0,00013	-0,14	Artery - Tibial
ENSG00000070961.15	ATP2B1	chr12_90005574_C_T_b38	rs11105483	0,00013	-0,14	Artery - Tibial
ENSG00000070961.15	ATP2B1	chr12_90015717_C_T_b38	rs61926987	0,00013	-0,14	Artery - Tibial
ENSG00000070961.15	ATP2B1	chr12_90159905_G_A_b38	rs6538230	0,00014	-0,16	Artery - Tibial
ENSG00000070961.15	ATP2B1	chr12_89547597_G_A_b38	rs4842665	0,00015	0,12	Artery - Tibial
ENSG00000070961.15	ATP2B1	chr12_89865713_T_C_b38	rs2722213	0,00015	-0,12	Esophagus - Mucosa
ENSG00000070961.15	ATP2B1	chr12_89866278_G_A_b38	rs2553097	0,00015	-0,12	Esophagus - Mucosa
ENSG00000070961.15	ATP2B1	chr12_89875126_G_A_b38	rs2553104	0,00015	-0,12	Esophagus - Mucosa
ENSG00000070961.15	ATP2B1	chr12_90071866_G_A_b38	rs6538222	0,00015	-0,13	Lung
ENSG00000070961.15	ATP2B1	chr12_89851601_T_C_b38	rs2553101	0,00015	-0,13	Esophagus - Mucosa
ENSG00000070961.15	ATP2B1	chr12_89637618_C_T_b38	rs10858914	0,00017	-0,12	Artery - Tibial
ENSG00000070961.15	ATP2B1	chr12_90010069_T_C_b38	rs4393376	0,00017	-0,15	Artery - Tibial
ENSG00000070961.15	ATP2B1	chr12_90016872_A_C_b38	rs6538217	0,00017	-0,15	Artery - Tibial
ENSG00000070961.15	ATP2B1	chr12_89904501_A_C_b38	rs7965284	0,00017	-0,18	Artery - Tibial
ENSG00000070961.15	ATP2B1	chr12_90128372_C_T_b38	rs10858959	0,00019	-0,13	Artery - Tibial
ENSG00000070961.15	ATP2B1	chr12_90129926_G_A_b38	rs10858960	0,00019	-0,13	Artery - Tibial
ENSG00000070961.15	ATP2B1	chr12_89943230_A_C_b38	rs4842696	0,0002	-0,14	Esophagus - Mucosa
ENSG00000070961.15	ATP2B1	chr12_89345678_C_G_b38	rs11105262	0,00022	0,21	Artery - Tibial
ENSG00000070961.15	ATP2B1	chr12_89861997_C_A_b38	rs1946327	0,00023	-0,12	Esophagus - Mucosa
ENSG00000070961.15	ATP2B1	chr12_90003409_T_C_b38	rs4453292	0,00023	-0,15	Artery - Tibial
ENSG00000070961.15	ATP2B1	chr12_90162353_T_C_b38	rs2731241	0,00023	-0,16	Artery - Tibial
ENSG00000070961.15	ATP2B1	chr12_90164205_A_G_b38	rs1438980	0,00023	-0,16	Artery - Tibial
ENSG00000070961.15	ATP2B1	chr12_89434120_G_A_b38	rs118130015	0,00027	0,33	Artery - Tibial
ENSG00000070961.15	ATP2B1	chr12_90173201_C_T_b38	rs1347851	0,00027	-0,15	Artery - Tibial
ENSG00000070961.15	ATP2B1	chr12_89623959_G_T_b38	rs2070759	0,00028	-0,11	Artery - Tibial
ENSG00000070961.15	ATP2B1	chr12_89646107_G_A_b38	rs939329	0,00028	-0,11	Artery - Tibial
ENSG00000070961.15	ATP2B1	chr12_90149730_G_A_b38	rs10777230	0,00028	-0,12	Artery - Tibial
ENSG00000070961.15	ATP2B1	chr12_89857319_C_T_b38	rs12371308	0,00028	-0,13	Artery - Tibial
ENSG00000070961.15	ATP2B1	chr12_89857983_T_C_b38	rs11105439	0,00028	-0,13	Artery - Tibial

Appendix table S4. eQTLs SNPs for the *ATP2B1* gene from the "Genotype-Tissue Expression" (GTex) database (P <1 ×10^{-6}).

In-silico analysis pipeline identifying the presence of noncoding variants in the ATP2B1 locus located in putative elements responsible for transcriptional regulation (EQTLs). The 77 SNPs that act as eQTLs for the *ATP2B1* gene annotated with prediction functional scores calculated from the "Genome-Wide Annotation of Variants" GWAVA tool. Eight SNPs are not reported in GWAVA, and are thus excluded. Data given include ID, chromosome, position, and region score. Bold: the top six SNPs selected.

ID	Chr.	Position	Region score
**rs11105352**	**12**	**90.026.462**	**0,63**
**rs11105353**	**12**	**90.026.463**	**0,61**
**rs10777221**	**12**	**90.441.215**	**0,52**
**rs73437358**	**12**	**90.072.076**	**0,48**
**rs111337717**	**12**	**90.037.506**	**0,47**
**rs2681492**	**12**	**90.013.089**	**0,47**
rs7136259	12	90.081.188	0,46
rs17249754	12	90.060.586	0,43
rs11105382	12	90.107.272	0,43
rs73437338	12	90.054.619	0,42
rs2280715	12	90.103.702	0,42
rs149193253	12	90.418.172	0,42
rs4842699	12	90.345.607	0,41
rs114744356	12	90.400.573	0,41
rs7488223	12	90.404.051	0,41
rs11105354	12	90.026.523	0,4
rs11105337	12	89.993.507	0,39
rs2681472	12	90.008.959	0,39
rs11105364	12	90.069.276	0,39
rs10777217	12	90.385.294	0,39
rs10745511	12	90.097.667	0,38
rs2408046	12	90.109.337	0,38
rs1401982	12	89.989.599	0,37
rs12579302	12	90.050.503	0,37
rs4842675	12	90.075.243	0,37
rs11105379	12	90.095.173	0,37
rs7299436	12	90.113.070	0,37
rs73208536	12	90.428.028	0,37
rs8181784	12	89.828.591	0,36
rs10777163	12	89.830.720	0,36
rs6538195	12	90.062.376	0,35
rs4842676	12	90.091.782	0,35
rs2113894	12	90.099.397	0,35
rs1980235	12	90.110.782	0,35
rs7488974	12	90.442.001	0,35
rs150184539	12	90.443.478	0,35
rs150119566	12	90.537.682	0,35
rs12230074	12	90.090.867	0,34
rs1358350	12	90.101.892	0,34
rs73437382	12	90.102.945	0,34
rs11105381	12	90.106.835	0,34
rs2681485	12	90.025.622	0,33

rs11105368	12	90.074.441	0,33
rs11105378	12	90.090.741	0,33
rs7313874	12	89.965.049	0,32
rs1689040	12	89.978.233	0,32
rs111478946	12	90.058.842	0,32
rs11105383	12	90.107.306	0,32
rs12579052	12	90.132.147	0,32
rs1594565	12	90.779.022	0,32
rs10858918	12	90.096.345	0,31
rs4408360	12	90.394.861	0,31
rs11105287	12	89.829.470	0,3
rs7968803	12	90.390.780	0,3
rs4842666	12	89.941.549	0,29
rs11105328	12	89.942.390	0,29
rs10858917	12	90.088.790	0,29
rs7302816	12	89.950.320	0,28
rs1590008	12	90.107.725	0,28
rs4466894	12	90.362.052	0,27
rs55849728	12	90.533.915	0,27
rs11105375	12	90.088.580	0,26
rs4842697	12	90.337.046	0,26
rs77187603	12	90.531.484	0,25
rs10858944	12	90.419.192	0,24
rs11105376	12	90.088.768	0,23
rs4842701	12	90.367.700	0,19
rs117742247	12	89.991.313	0,16
rs57481061	12	90.019.178	0,14

Appendix table S5. Differentially expressed genes in HEK-293T edited cells

Differentially expressed (DE) genes comparing differentially expressed genes from RNAseq analyses comparing HEK293T (WT T/T) and isogenic generated clones (C/C). Data given include gene name, BaseMean (average of the normalized count values), FoldChange, log2Fold Change, pValue and BH adjusted p value are listed.

Gene Symbol	C.C. / T.T. base Mean	C.C. / T.T. Fold change	C.C. / T.T. Log2 Fold change	C.C. / T.T. .raw. pval	C.C. / T.T. .bh. pval
TMEM189-UBE2V1	83,432186	-8,030776	0,449601396	2,31086E-11	4,1064E-07
CCND1	1001,397770	-5,516491	0,398915171	6,5693E-10	5,83682E-06
BRINP3	286,533286	2,984710	0,262334776	1,81388E-09	1,07442E-05
SGPP2	407,418822	-2,731977	0,265271423	4,60582E-08	0,000204614
ACTRT3	160,576777	2,871753	0,284993336	9,28257E-08	0,000329903
KCNB1	82,902050	-8,504763	0,594124921	2,01426E-07	0,000596558
UBAC2-AS1	137,141414	3,239612	0,33518791	4,20766E-07	0,00097994
LOC105274304	42,515853	5,209731	0,471500485	4,41166E-07	0,00097994
DECR1	969,930136	2,615839	0,279833059	7,14043E-07	0,001409838
TUNAR	59,840352	-7,617012	0,598726825	9,96018E-07	0,001769925
AIFM2	413,442074	3,150496	0,344923044	1,58785E-06	0,002565095
IFI30	824,717238	2,366240	0,263042902	2,31328E-06	0,003425588
LINC00839	176,086803	-3,580646	0,400302795	4,28474E-06	0,00585691
IFIT1	334,126293	2,561404	0,306536035	9,56975E-06	0,012146741
ZNF442	58,360208	-5,030546	0,538613852	1,50985E-05	0,017471828
LOC105374338	57,358380	-10,111528	0,772992986	1,57315E-05	0,017471828
STC1	61,570815	-13,594827	0,874836971	1,68012E-05	0,017562241
TP53I3	647,230208	3,278584	0,404223866	2,25589E-05	0,020025016
SORCS1	580,443344	-5,656138	0,587216634	2,07132E-05	0,020025016
C11orf96	232,808814	-3,155106	0,391077311	2,24744E-05	0,020025016
ETNPPL	89,902382	2,369733	0,29823272	2,99735E-05	0,02417569
MAP6	113,007758	-2,857277	0,364924211	3,31659E-05	0,024556566
NAALAD2	172,160859	2,522826	0,324404345	3,86575E-05	0,027025416
CRTAC1	151,000507	-3,739295	0,464430892	4,18567E-05	0,027025416
ZNF83	296,337238	-4,632727	0,540401733	4,25837E-05	0,027025416
SLC9A9	260,880735	2,474374	0,318723781	4,11494E-05	0,027025416
DUSP4	199,388914	-3,327344	0,425797324	4,63653E-05	0,027463719
CACNA1C	285,991249	-2,298536	0,300564281	6,47297E-05	0,032293583
KCND3	60,278431	-3,500644	0,453509921	6,72404E-05	0,032293583
TAC1	32,763155	-6,635666	0,679767066	5,90848E-05	0,032293583
HCAR1	30,689060	3,294148	0,427826276	5,81729E-05	0,032293583
SH3BGRL3	1332,907483	2,280879	0,297318012	6,30567E-05	0,032293583
ZFAS1	3127,906649	-2,477187	0,326245427	6,03582E-05	0,032293583
INSC	111,875918	-5,631494	0,631212073	7,80323E-05	0,034665871

CA5BP1-CA5B	466,096834	-2,141693	0,27781154	7,65262E-05	0,034665871
CALCB	550,253073	-3,207646	0,427394581	8,34263E-05	0,034945057
FAM189A1	428,197582	-2,040489	0,261739715	8,45715E-05	0,034945057
SLC10A4	97,279503	2,640249	0,356808371	8,65269E-05	0,034945057
LOC105376600	64,661519	2,868588	0,3861753	8,25346E-05	0,034945057
APOBEC3B	398,824054	4,451726	0,552665277	9,69358E-05	0,038278864
MYO7A	138,110413	3,845872	0,500531182	0,000103389	0,039405917
MIR503HG	382,949889	2,740963	0,377910205	0,000118465	0,042102402
DTX4	43,232806	-2,915700	0,404222777	0,000133839	0,044873876
RASGRP1	252,508176	-2,536054	0,353223386	0,000144141	0,047433015
GPR85	89,893659	3,055436	0,424745359	0,000148388	0,047942902
INSM1	333,792159	-2,640916	0,370350247	0,000154944	0,049167057
FOXL2NB	69,535137	2,242507	0,310092596	0,000171754	0,051335806
ZNF887P	141,080914	-2,027753	0,271087935	0,000168438	0,051335806
BCL11B	128,012163	-2,141969	0,293595502	0,000181812	0,052964053
TNFSF12	92,089181	5,655907	0,674469239	0,000210342	0,057504346
GFRA3	101,151302	6,134739	0,711668641	0,000235738	0,061603889
FAM131B	181,464732	-5,544801	0,674009661	0,000246065	0,063370689
ZNF44	448,348209	-2,660575	0,386100663	0,000255784	0,064017982
UNC5A	171,595731	-2,055024	0,284091889	0,000254375	0,064017982
HLA-DPB1	257,589645	4,096743	0,557369411	0,000262095	0,0646866
H4C11	59,272138	3,145864	0,456588286	0,000293094	0,070382225
SERPINB9	222,213079	2,565982	0,376759785	0,000308057	0,072028525
DCDC2	111,351198	3,554733	0,508187792	0,00031758	0,073290751
B3GAT1	55,924350	-6,020004	0,721809715	0,000333382	0,075951172
H4C12	52,248215	2,875846	0,428253452	0,000372825	0,083861984
ITGA8	1480,265031	2,565157	0,383623932	0,000396122	0,087988497
EGR2	57,211147	-3,256387	0,48351939	0,000427247	0,090382994
CREG2	138,836938	-3,811626	0,547340446	0,000420481	0,090382994
GLT8D2	491,209835	2,068799	0,298657443	0,000445259	0,093085281
MAGEH1	23,032460	3,765570	0,545761431	0,000456687	0,094364358
KCNA3	174,522777	-5,306064	0,694577979	0,0005276	0,099863623
KCNH2	367,978109	-2,119386	0,312378761	0,000522372	0,099863623
PPP2R2C	623,556841	-3,178508	0,480416645	0,000515224	0,099863623
H2BC11	104,296047	3,505272	0,521191634	0,000516785	0,099863623
H2BC12	5516,018025	2,972387	0,452599321	0,000515759	0,099863623
TMEM271	66,685863	-5,078329	0,683493121	0,000603671	0,107272367
TNC	225,868755	-3,865169	0,569370153	0,000613059	0,107861917
MPPED1	79,902676	-2,725473	0,422693183	0,000621349	0,10824876
GRIP2	536,880773	-2,409086	0,374771203	0,000712575	0,121754415
H2BC4	158,261088	3,957894	0,587408093	0,000728037	0,123145543
PLEKHB1	991,447366	2,364495	0,3682189	0,000746997	0,124057381
CXCL12	497,709536	2,440144	0,383127391	0,000781944	0,127469281
IGSF21	19,475213	-3,463513	0,533943272	0,000789061	0,127469281
CCDC144CP	89,717015	-2,343600	0,367716468	0,000833258	0,132440319

LOC105374985	122,075149	3,935212	0,591569805	0,000834739	0,132440319
UTS2R	669,584487	2,216588	0,345007443	0,000873299	0,135418787
WFIKKN2	26,461499	3,677875	0,564654428	0,000876374	0,135418787
H3C10	84,134491	4,237925	0,627580818	0,000901255	0,138062951
EPSTI1	127,915454	2,507306	0,400637841	0,000932738	0,140464055
H2AC19	254,781970	3,581659	0,55686518	0,000948618	0,141654904
NHS	387,764739	-2,075790	0,320358692	0,001005432	0,146278352
H2AC18	241,818396	3,582827	0,560110286	0,001012506	0,146278352
CNFN	134,566492	2,034835	0,311705432	0,00100874	0,146278352
LOC102724334	45,875395	4,157796	0,626222775	0,001027497	0,147246965
H2BS1	47,617542	4,141893	0,627330158	0,001082034	0,15260112
RBP1	498,109755	2,335820	0,375359291	0,001111426	0,155512164
MYT1	336,558529	-2,496780	0,405195824	0,001122582	0,155845922
IGSF10	166,452007	2,822054	0,460107759	0,001141803	0,157285548
CLCNKB	71,124774	2,626501	0,429119494	0,001168278	0,15854326
CD2BP2-DT	102,902309	2,125394	0,33505821	0,001168777	0,15854326
SCN2A	118,744369	4,382325	0,660897543	0,001257718	0,166966859
LOC105377101	43,642001	2,453621	0,401504402	0,001259064	0,166966859
GABBR2	275,256463	-2,302025	0,37357461	0,001281988	0,167174498
ADPRHL1	206,292022	2,044902	0,320362946	0,001275461	0,167174498
GET1-SH3BGR	244,495810	-4,533027	0,678679707	0,001314393	0,169251913
MYD88	450,900886	2,744254	0,45372257	0,001327711	0,1697369
CHRNA4	93,363994	-5,174757	0,743578819	0,001426209	0,175792593
NOTUM	121,190271	-2,438830	0,402652245	0,00140174	0,175792593
ABHD14A-ACY1	86,989168	4,121113	0,640588902	0,001426147	0,175792593
RN7SL832P	86,608086	2,603285	0,432534517	0,001416503	0,175792593
PCDH1	249,128800	-3,070607	0,514882728	0,001669514	0,193903631
FSTL4	105,636621	-3,452507	0,568575036	0,001666109	0,193903631
LOC105379452	18,840817	-7,236213	0,91124622	0,001728351	0,196876904
LOC107987172	224,587454	-2,233874	0,369907133	0,001720297	0,196876904
CHGA	52,623770	2,281134	0,380346509	0,001759575	0,197896488
NEFL	3076,889048	-2,324726	0,39182036	0,001895273	0,211817637
IRS2	774,762114	-2,430070	0,412667393	0,001908012	0,211908554
DGUOK-AS1	61,009747	3,091032	0,525311791	0,001939911	0,214113167
RIC3	16,087736	-6,163998	0,849278443	0,002004773	0,219906273
LOC105372431	20,335433	-3,024195	0,517910682	0,002051481	0,223616918
OSTN	265,011136	-4,127151	0,669869737	0,002265276	0,235513736
KCP	63,009142	3,034456	0,524561128	0,002266339	0,235513736
B4GALNT4	1789,211576	-2,360494	0,406635152	0,002310023	0,23865763
H2AC20	38,358109	3,148083	0,544654169	0,002384238	0,243495518
GAPDHP21	66,226989	2,039074	0,338965735	0,002425363	0,243495518
H2BC21	467,016242	2,680520	0,470178014	0,002482406	0,247822236
CD70	238,434988	-2,135637	0,362964621	0,00256215	0,252686977
ZNF804A	161,621028	2,502031	0,442391564	0,002782586	0,26727862

PDGFA	321,135004	-2,431151	0,432302092	0,003029935	0,284755132
H2AC6	159,268699	3,655072	0,632378301	0,003107166	0,288759469
LOC105376043	16,002502	-7,968389	1,013067823	0,003119967	0,288759469
LAMA2	84,810986	2,212674	0,390290428	0,003327536	0,30168526
HLA-DRB1	261,379742	2,361657	0,423085885	0,003385552	0,305387139
C1R	326,419676	2,102984	0,366464971	0,003428663	0,306167512
CDO1	280,605339	2,471103	0,446550508	0,003469559	0,308270276
PTX3	196,772221	-3,196000	0,575713778	0,003595448	0,310480211
H4C8	36,880186	4,138057	0,703235663	0,003572774	0,310480211
LINC00673	63,137675	-2,367503	0,426173496	0,003528355	0,310480211
RNF103-CHMP3	23,006288	4,909577	0,790049364	0,003665034	0,313975487
DUSP5	188,637928	-2,127848	0,376078681	0,003770855	0,315387624
LGALS1	484,005465	3,165096	0,574286868	0,00379814	0,315387624
RSU1	4483,402702	2,096227	0,368836706	0,003791163	0,315387624
LOC100506691	154,987603	-2,603430	0,477442577	0,003836928	0,317126558
MAFF	183,948512	-2,747302	0,506155611	0,003969603	0,321912334
C2orf50	30,828723	2,691050	0,496331917	0,004009059	0,321912334
PAXIP1-AS2	96,613543	2,093501	0,370134505	0,003979131	0,321912334
PPM1F-AS1	126,491921	2,321619	0,422439835	0,00402164	0,321912334
RPL41P3	994,823529	-6,363841	0,930643075	0,004119357	0,326790034
ZNF441	217,268996	-2,460594	0,453645679	0,004190123	0,327153842
MIR3659HG	35,909683	-2,917769	0,539273373	0,004173825	0,327153842
IFI6	516,531609	2,037603	0,359182836	0,00425093	0,328430553
FAM20A	18,469118	-4,573073	0,769258599	0,004358061	0,330435232
PHF21B	829,424746	-2,110548	0,377709072	0,004330359	0,330435232
UBE2E1-AS1	30,518553	3,030628	0,561304591	0,004374402	0,330435232
FOXI3	405,501943	-2,235103	0,408071728	0,004462465	0,334590736
FAM21EP	28,952462	2,440531	0,454598738	0,004633063	0,344475022
GNG11	177,289717	2,388944	0,444408438	0,004697628	0,347820231
LOC151760	37,555359	4,055385	0,717594844	0,004881754	0,359953422
EBF4	926,500813	-2,105279	0,382956348	0,005039054	0,366983561
CLTRN	91,214118	-2,208317	0,408660804	0,005160954	0,374310337
HLA-DMA	129,970696	2,791911	0,530479769	0,005233657	0,376526647
DUSP8	1009,106859	-2,124815	0,391010065	0,005421802	0,382323077
AMER2	85,303773	-4,903363	0,827422472	0,00556807	0,386300756
NEXMIF	152,020623	-2,379328	0,451180425	0,005575786	0,386300756
LOC105372793	60,164013	-2,645564	0,505870059	0,005527368	0,386300756
ARMC3	44,004830	3,301865	0,623115799	0,005682036	0,391355711
H1-2	1253,717094	4,372085	0,778833812	0,006281695	0,402363213
NGF	28,998625	-5,312023	0,878278419	0,006085037	0,402363213
H2AC13	57,802894	2,912808	0,564885206	0,006324124	0,402363213
SUSD2	48,559757	2,332326	0,44645699	0,006207968	0,402363213
ZNF860	30,609216	3,327763	0,636034472	0,006388669	0,402363213
SOX21-AS1	454,193051	-2,288834	0,43839043	0,006430195	0,402363213
STAB1	28,840577	4,147057	0,755452179	0,006600202	0,405872299

MARVELD3	125,416640	-2,042442	0,378984335	0,006556484	0,405872299
FOXCUT	173,477735	2,678922	0,524240057	0,006691182	0,410007933
ZNF460	293,617721	-2,224608	0,427585615	0,006979412	0,411478038
A2M-AS1	60,090907	2,706839	0,531927131	0,00691806	0,411478038
LOC105375740	86,770649	-2,370976	0,46056627	0,006846118	0,411478038
NQO1	351,818692	2,235962	0,430797178	0,007043902	0,412818205
TCN2	85,481631	3,841755	0,720806098	0,007062551	0,412818205
H2AJ	362,220293	2,430186	0,476221574	0,007143814	0,412818205
LOC107985805	69,525191	2,079098	0,393589469	0,007298886	0,413061172
PAX2	233,687579	-2,136228	0,40852503	0,00735046	0,41334709
ASNSP1	344,657840	-3,151973	0,617752823	0,007338129	0,41334709
TAPT1-AS1	123,498240	-2,096041	0,39921997	0,00748669	0,418864575
CXCR4	665,122921	-2,221408	0,431640906	0,007637986	0,421512451
HMOX1	1093,831527	2,024364	0,382354463	0,007789452	0,426123586
SULF1	1407,221782	-2,493240	0,495333451	0,007793714	0,426123586
FAM78B	54,049146	-2,562930	0,511916417	0,007992772	0,430292346
LINC01597	37,243723	-2,502565	0,499487631	0,008060368	0,430292346
EXOSC10-AS1	39,898137	2,091504	0,401575181	0,008027439	0,430292346
SNHG32	4816,524514	-2,008954	0,38031661	0,008136959	0,432915455
H2AC11	72,462803	2,602216	0,522608452	0,008288056	0,435951051
UBL4B	8,989938	-6,065390	0,984368804	0,008244327	0,435951051
MASP1	21,080358	-4,784451	0,85785675	0,008474581	0,441072181
SETBP1	104,230686	-4,504099	0,827547562	0,008697979	0,442373736
DENND1C	166,681322	2,236305	0,442279609	0,00865723	0,442373736
ABCC6P2	58,605638	2,124452	0,414419093	0,00871163	0,442373736
LOC105375519	123,303250	2,201741	0,43332124	0,008596054	0,442373736
SPINK5	56,960169	3,380029	0,671658903	0,00889759	0,446638892
DNAH17	660,196411	-12,991896	1,415355257	0,008952578	0,44813329
GNG4	2828,753979	-2,154744	0,424255402	0,009041138	0,450555045
CCDC116	50,743509	2,828669	0,574753817	0,009053493	0,450555045
ABCF2-H2BE1	79,017769	-3,696435	0,723836331	0,009167405	0,452063237
SH2D3C	173,250310	-3,670695	0,722574101	0,009422087	0,452514835
SOX21	192,512570	-2,283514	0,457850407	0,009272394	0,452514835
TMED10P2	13,875128	-4,819407	0,871634639	0,009241487	0,452514835
SHC2	684,471429	-2,390509	0,485047011	0,009537637	0,453549176
RFTN2	82,707924	2,864247	0,588418628	0,009878295	0,461769397
LOC105379504	41,293086	-2,451935	0,501916547	0,009938537	0,461769397
IFIT3	43,831074	2,507580	0,51614904	0,010181648	0,462265878
KCNA2	82,223961	-2,978108	0,611573092	0,010043193	0,462265878
PTPN6	78,379271	2,699979	0,557503478	0,010161193	0,462265878
LDHD	129,209991	2,010303	0,391882921	0,010149346	0,462265878
LOC101927372	254,006279	-2,567526	0,528534871	0,010056872	0,462265878
LOC114841040	32,439531	2,586396	0,533564783	0,010187365	0,462265878
DAB1	90,481342	-3,717556	0,738909645	0,010355817	0,46706311
LOC644285	414,486063	-2,280702	0,463925576	0,010349105	0,46706311

LOC105374334	32,195839	4,638824	0,863822353	0,010384734	0,467181598
ALOX5	68,900380	-2,642849	0,54872954	0,010613777	0,473270214
LOC105376483	15,592257	3,677065	0,735519121	0,010647714	0,473270214
SLCO3A1	50,987492	-2,509594	0,520864958	0,010816953	0,474027365
KRT222	194,081512	2,808758	0,585660287	0,010958442	0,47441647
PDE1A	76,600583	2,668715	0,559252946	0,011334662	0,475644889
WNT4	40,266779	-3,593715	0,731933331	0,011689823	0,475644889
ERAP2	220,731598	2,214322	0,454493509	0,011622886	0,475644889
H3C14	19,132920	3,126135	0,649555568	0,011356085	0,475644889
SCARA5	71,406056	-4,983017	0,918286074	0,011629218	0,475644889
LOC100420528	20,732670	2,665940	0,557335307	0,011141539	0,475644889
MYOF	98,844708	2,405213	0,503064848	0,011839179	0,477389443
PTHLH	67,818047	-2,799376	0,591552441	0,012055277	0,482482583
SAMD9	284,810135	2,572262	0,544851383	0,012360968	0,486358865
LOC107986617	17,359839	3,855924	0,77857966	0,012391103	0,486358865
LOC107987250	48,694225	2,309197	0,482601656	0,012355056	0,486358865
CALCRL	179,524358	2,819903	0,599296854	0,012572062	0,487190171
H3C15	18,019846	3,213396	0,674335694	0,012510092	0,487190171
LPP-AS2	27,195223	2,953635	0,626554803	0,01263898	0,487190171
FOSL2	498,345889	-2,160202	0,449119202	0,013357203	0,491346421
GPER1	43,831085	2,068560	0,424244067	0,013445204	0,491346421
ONECUT1	80,375529	-2,513206	0,53652296	0,013210403	0,491346421
LOC105378808	60,735803	-2,405349	0,511242528	0,013256575	0,491346421
LOC105370363	22,128300	2,915329	0,625509733	0,013593049	0,49295607
GALR2	18,776678	2,690987	0,582278692	0,014180182	0,505122879
MGC16275	19,913205	2,699908	0,584681385	0,014255746	0,505122879
PURPL	905,326998	-2,178588	0,458829099	0,014349526	0,505122879
PCAT7	44,179737	2,529582	0,547027132	0,014381473	0,505122879
LOC107986673	32,149159	-2,555470	0,55349272	0,014463524	0,505122879
BHLHE40	3211,357365	-2,035148	0,420187279	0,01469918	0,507367599
ZNNT1	193,418348	-2,055119	0,426198756	0,014754478	0,507367599
ABCC2	147,085656	-2,064318	0,429579076	0,014926274	0,5081224
MID1	8398,040743	2,442604	0,530037031	0,015065038	0,509915674
SCN3B	251,318239	-3,127228	0,678378073	0,015319743	0,513718244
LOC105376504	61,762559	-3,448586	0,737366292	0,015429375	0,515375921
MAGEB2	150,733781	5,985202	1,066853118	0,015535903	0,517960597
SAMD11	3551,242289	2,512037	0,550171652	0,015720042	0,520195799
LMCD1	280,590523	-2,331061	0,50615077	0,015852283	0,522625367
LOC101928092	140,132142	-2,301133	0,501431022	0,016492685	0,528692213
LOC101927441	20,980243	2,953978	0,652620361	0,016645856	0,530308548
LOC105371870	96,721458	-2,043582	0,430801613	0,016690937	0,530308548
H3C6	480,760988	2,091117	0,445043835	0,016784642	0,530717248
PHYH	1351,003601	2,079548	0,442799167	0,017058774	0,531333198
VAMP8	885,013422	2,171423	0,468580666	0,016972804	0,531333198
ZNF528	51,419408	-2,789087	0,620849434	0,017149	0,531333198

LOC105379392	30,522192	2,168717	0,468142051	0,017047111	0,531333198
H4C15	848,081978	2,326789	0,513961658	0,017764578	0,543333142
ZSCAN18	496,028424	-2,477565	0,553161678	0,01796893	0,544894016
BST1	42,447076	-2,733265	0,613235969	0,018004493	0,545042312
H2BC5	325,329786	2,052075	0,43867119	0,018071646	0,546144805
EXTL1	39,272401	2,955607	0,662931628	0,01835417	0,550006069
FANCF	24,796607	3,274535	0,726371963	0,01847581	0,55028118
LOC105375010	29,577349	-2,326060	0,51692616	0,018471957	0,55028118
HCN4	200,515863	-2,341954	0,52183483	0,018638418	0,551089327
MIR1915HG	622,393508	2,143832	0,467832047	0,018688765	0,551660042
SHBG	16,336178	2,600599	0,586773003	0,018779639	0,552506929
LOC105374493	34,884114	-2,082786	0,450736212	0,018853601	0,552852287
SMIM2-AS1	39,907480	2,069649	0,447096195	0,018919456	0,553627805
LOC112268241	16,162227	2,576821	0,58192947	0,018942358	0,553627805
PTPRO	109,503379	-3,192074	0,717533457	0,019612448	0,564851214
GABRQ	117,505298	-3,325180	0,743274717	0,019692484	0,565850958
ST6GALNAC5	58,661560	-2,277628	0,509295209	0,019715577	0,565850958
AGGF1P1	75,236533	-2,001329	0,429735175	0,019846073	0,566829963
BTBD19	19,815384	-2,256164	0,505427563	0,020204302	0,569046054
TOP1P1	20,975574	2,395720	0,545105753	0,020760003	0,576763449
LBH	348,392939	-2,007632	0,436482617	0,021243329	0,580922357
JMJD1C-AS1	20,547817	2,177953	0,486735487	0,021046359	0,580922357
CRHR1	74,163037	-3,119579	0,714829279	0,021667636	0,587838014
ALDH3A1	51,564457	2,030258	0,445706127	0,021892099	0,589428174
TOGARAM2	64,410262	2,032265	0,446168429	0,021844844	0,589428174
LY6G6F-LY6G6D	30,422044	2,837476	0,656716005	0,021957089	0,589475932
ISM1	168,213279	2,253060	0,514240247	0,022674901	0,596937774
HLA-DRB5	66,684569	2,095448	0,474913907	0,024622952	0,6124707
PLXNC1	125,599244	-2,032641	0,455195742	0,024565674	0,6124707
OLFM1	87,194852	-2,502325	0,588324214	0,024498682	0,6124707
ZMAT1	154,767848	-2,057124	0,460991629	0,023984921	0,6124707
COL23A1	161,782803	-2,293905	0,533073397	0,024641254	0,6124707
GRIN3B	75,913529	-2,122059	0,480329354	0,023831561	0,6124707
TCAM1P	292,359082	-2,164467	0,494167664	0,024176086	0,6124707
SNORA11F	26,338611	2,425985	0,565904865	0,023862346	0,6124707
LINC02154	46,614149	-4,990886	1,026013531	0,023790692	0,6124707
PVALB	47,946661	2,151926	0,493283792	0,025002553	0,613180123
LAMB2P1	81,418210	-2,224735	0,515092519	0,025112833	0,613180123
PLPP4	83,437231	-2,586149	0,6118097	0,02505361	0,613180123
HAPLN2	66,310597	-2,150832	0,493860281	0,025269234	0,613519736
ZNF136	547,872865	-2,288885	0,537473715	0,026235772	0,618327315
DLEC1	69,802038	-2,346366	0,557397501	0,027282522	0,618327315
ZNF681	67,945174	-3,170104	0,753168711	0,027102651	0,618327315
SOX1-OT	27,428964	-2,472606	0,588381895	0,026438999	0,618327315
LOC107987248	27,404747	-2,656373	0,638453499	0,027271334	0,618327315

LOC107987251	24,939658	-2,492595	0,591782975	0,025975741	0,618327315
LEF1-AS1	10,382565	2,965153	0,712581027	0,027764282	0,62136913
DST-AS1	18,634134	2,385808	0,570050732	0,027761299	0,62136913
LOC105369250	54,782954	-2,358759	0,563292125	0,027960272	0,624188491
INSM2	11,886420	-4,562444	0,998014051	0,028223603	0,626134106
CHST8	147,860459	-2,191799	0,516440039	0,028368458	0,62747547
LOC102724378	107,836594	2,044709	0,471499984	0,028630399	0,631967839
CILP2	1056,471899	2,191085	0,51891651	0,029199336	0,638129205
LOC105376328	35,957406	3,257089	0,781766807	0,029320919	0,638129205
BST2	129,190668	-2,603304	0,637831445	0,030455147	0,638242279
HRH1	31,364331	-3,547574	0,839254605	0,029500518	0,638242279
IGF2	298,928826	-2,190637	0,523733752	0,030760052	0,638242279
SPOCK1	74,099071	-2,533698	0,620688448	0,030703218	0,638242279
HRK	1442,061989	-2,067082	0,482684959	0,029980322	0,638242279
CEACAM19	421,866469	-2,111206	0,496631935	0,029949543	0,638242279
AGAP11	226,224745	-4,149321	0,945037251	0,029835361	0,638242279
LOC100132813	26,858145	3,591384	0,848840792	0,029779515	0,638242279
TGM2	102,982558	2,036974	0,476814833	0,031344216	0,641216622
GRM4	33,914389	-2,166065	0,518157665	0,031397154	0,641295886
IL6R	455,226024	-2,414722	0,597222214	0,033202873	0,657379503
STUM	712,383401	-2,019302	0,475845422	0,033118578	0,657379503
KRT8	1692,119095	-3,237575	0,803462327	0,034900367	0,659904218
PDE1B	121,592956	-2,420183	0,614313225	0,037923755	0,659904218
TF	53,626629	-2,140486	0,526316224	0,036971291	0,659904218
TNXB	126,148950	-2,777447	0,700142904	0,03529643	0,659904218
H2AC17	44,397996	2,123504	0,514353935	0,034664515	0,659904218
H3C2	37,279100	2,441449	0,611098822	0,035095964	0,659904218
H4C14	171,407851	2,145295	0,529799692	0,037665465	0,659904218
RBMXL2	7,056080	-4,092136	0,970121257	0,036129778	0,659904218
TBX21	23,823518	-3,535255	0,87011824	0,036281811	0,659904218
MXRA8	742,309992	4,938746	1,100322983	0,036254433	0,659904218
FBXO43	27,910448	2,324499	0,586318879	0,037937867	0,659904218
NKX1-2	328,033589	-2,063241	0,498482414	0,03606565	0,659904218
LOC101929174	40,160169	-2,477070	0,620696243	0,03500213	0,659904218
LOC105370910	97,580591	-2,037697	0,492260299	0,03696304	0,659904218
CCDC153	45,688284	2,117462	0,521734304	0,038033238	0,660655556
GFAP	56,207540	2,525608	0,645819608	0,038483651	0,665228083
TXLNB	67,542515	2,138848	0,532055281	0,039255018	0,672672778
SUGT1P4-STRA6LP	90,262837	2,070079	0,509493046	0,039374107	0,672767195
ADCYAP1R1	465,193709	-2,198484	0,551971334	0,039494182	0,674170625
LOC105371532	14,132693	2,467460	0,634079108	0,039879884	0,676255363
LOC729557	9,871495	-3,513475	0,883260477	0,040120401	0,676413214
SCARNA17	28,820570	-2,044434	0,503553726	0,040477633	0,676756734
MYRIP	88,728956	-3,270970	0,835919301	0,040823763	0,677578065
LOC100128164	19,536298	2,490301	0,644092608	0,040985032	0,677578065

LOC107985770	68,489353	2,396833	0,617532415	0,041130972	0,677578065
PTGER2	85,787112	2,029074	0,504340009	0,042962561	0,679242342
SORCS2	32,286955	-2,270660	0,580786738	0,04164157	0,679242342
DLGAP3	87,714925	-2,189738	0,557256084	0,042442684	0,679242342
NOD2	29,843526	-2,656171	0,696051762	0,042890477	0,679242342
LRRTM4	150,016199	2,702259	0,707208258	0,042567824	0,679242342
SYT9	129,882797	-2,769766	0,722887965	0,042033928	0,679242342
TCTE1	51,052442	-2,897185	0,755715189	0,042282642	0,679242342
LINC01402	10,805989	-3,217822	0,829104117	0,041989727	0,679242342
LOC105371017	36,450784	-2,459946	0,637256278	0,041565889	0,679242342
LOC105378833	20,204920	-2,245461	0,576124057	0,042803151	0,679242342
KCNMA1	74,965729	-2,250091	0,579673145	0,043554829	0,679530352
NTRK2	141,708054	-2,044637	0,510582031	0,04328823	0,679530352
PAX5	19,568319	-2,734964	0,717831779	0,043166855	0,679530352
LOC112268295	24,415084	-2,997949	0,783904476	0,043318754	0,679530352
IL11	44,702869	-2,752191	0,725039729	0,043958762	0,67984961
MRO	15,883299	2,329972	0,605762425	0,043956368	0,67984961
STAC2	260,470414	2,553762	0,6713543	0,043928815	0,67984961
MSANTD1	33,984032	-2,022199	0,50443212	0,044009807	0,680047188
THBS4	76,990769	-2,139081	0,545757687	0,044427726	0,681228534
SUGT1P3	19,701491	-2,308214	0,601111382	0,044688301	0,681311766
TMEM178A	54,875002	-2,018488	0,505256216	0,04491301	0,681468869
PHOSPHO1	96,998389	2,060482	0,520749767	0,045193765	0,682184047
LOC107986246	15,956279	-2,135533	0,546524024	0,045195192	0,682184047
SMIM38	89,398948	-4,752361	1,12486107	0,045603814	0,682746789
PEG10	51,475601	2,171752	0,560292324	0,045833778	0,683667226
C8orf34-AS1	36,077362	-2,149353	0,55358984	0,046143368	0,683667226
LOC105376010	162,695106	-4,282941	1,050605227	0,045769033	0,683667226
H2BC17	62,093953	2,172750	0,562547537	0,046580628	0,687489829
TNFRSF12A	702,530996	-2,201738	0,572560751	0,046736058	0,688639922
SNORD15A	12,494615	-3,166962	0,837004461	0,046926124	0,689367271
GNAO1	354,579308	-2,304205	0,606754546	0,047169963	0,69095057
SATB2-AS1	24,904431	2,408586	0,640765043	0,047796243	0,69481449
RPL5P30	13,576400	-3,342785	0,880028155	0,04788322	0,69481449
C15orf48	44,677075	-2,657012	0,717744712	0,049505175	0,706024844
LOC441087	36,410145	-2,278663	0,606125248	0,049960668	0,707138674

KEGG	N. of SigGenes	Pvalue	Bonferroni	FDR	Number of SigGenes UP	Number of SigGenes DOWN
Systemic lupus erythematosus	31	1,23E-38	2,83E-36	2,83E-36	31	0
Alcoholism	32	8,62E-36	1,98E-33	9,88E-34	27	5
Neutrophil extracellular trap formation	26	2,57E-26	5,89E-24	1,96E-24	26	0

Viral carcinogenesis	15	2,83E-11	6,49E-09	1,62E-09	13	2
**PI3K-Akt signaling pathway**	14	2,63E-07	6,02E-05	1,20E-05	3	11
Neuroactive ligand-receptor interaction	14	3,96E-07	9,06E-05	1,51E-05	4	10
Herpes simplex virus 1 infection	15	3,00E-06	6,87E-04	9,81E-05	6	9
Toxoplasmosis	8	5,21E-06	1,19E-03	1,49E-04	6	2
Necroptosis	9	5,88E-06	1,35E-03	1,50E-04	9	0
Intestinal immune network for IgA production	6	1,17E-05	2,68E-03	2,68E-04	5	1
Taste transduction	7	1,41E-05	3,23E-03	2,93E-04	2	5
Morphine addiction	7	1,95E-05	4,46E-03	3,72E-04	2	5
Pathways in cancer	14	2,21E-05	5,06E-03	3,89E-04	6	8
Viral myocarditis	6	3,18E-05	7,29E-03	5,21E-04	5	1
Inflammatory bowel disease	6	4,70E-05	1,08E-02	7,17E-04	4	2
Renin secretion	6	6,27E-05	1,44E-02	8,97E-04	2	4
**MAPK signaling pathway**	10	9,54E-05	2,18E-02	1,28E-03	1	9
Leishmaniasis	6	1,06E-04	2,43E-02	1,35E-03	6	0
**Calcium signaling pathway**	9	1,60E-04	3,67E-02	1,93E-03	1	8
Transcriptional misregulation in cancer	8	1,80E-04	4,12E-02	2,06E-03	5	3
GABAergic synapse	6	2,10E-04	4,81E-02	2,29E-03	1	5

Appendix table S6. Differentially expressed proteins in HEK293T-ACE2 cells upon treatment with 1 µM compound #7 for 24 h.

Proteomic assay based on LC-MS/MS approach performed on HEK-293T-ACE2 cells treated with PI-7 molecule (1 µM) for 24 h. In the treated cells, there were found 17 downregulated and 66 upregulated proteins. Data given include protein entry, protein names, gene names, log2 fold changes (log2FC), fold change (FC), Student's *t*-test false discovery rate (FDR), number of peptides (Razor + unique peptides), and proportion of sequence coverage (Unique + razon sequence coverage).

Entry	Protein name	Gene name	log2FC	FC	Student's *t*-test FDR	Razor + unique peptides	Unique + razor sequence coverage (%)
Q6FI81	Anamorsin	CIAPIN1	-0,92	0,53	3,71E-02	4	24,4
P55145	Mesencephalic astrocyte-derived neurotrophic factor	MANF	-0,87	0,55	4,88E-02	6	33
P10412	Histone H1.4	HIST1H1E	-0,73	0,60	5,44E-04	12	32,4
P51572	B-cell receptor-associated protein 31	BCAP31	-0,72	0,61	6,31E-03	7	26
P62280	40S ribosomal protein S11	RPS11	-0,70	0,61	5,76E-03	8	41,1
P47813	Eukaryotic translation initiation factor 1A, X-chromosomal	EIF1AX	-0,70	0,62	3,17E-03	6	34
P62750	60S ribosomal protein L23a	RPL23A	-0,62	0,65	3,54E-03	9	37,2
P62979	Ubiquitin-40S ribosomal protein S27a	RPS27A	-0,62	0,65	5,82E-05	12	55,8
P84090	Enhancer of rudimentary homolog	ERH	-0,58	0,67	1,32E-02	4	37,5
P60866	40S ribosomal protein S20	RPS20	-0,58	0,67	2,10E-03	4	28,6
P35613	Basigin	BSG	-0,57	0,67	2,79E-03	6	22,9
Q9H444	Charged multivesicular body protein 4b	CHMP4B	-0,57	0,67	5,54E-04	6	27,7
Q07020	60S ribosomal protein L18	RPL18	-0,56	0,68	9,09E-03	7	35,6
P42677	40S ribosomal protein S27	RPS27	-0,55	0,68	2,24E-04	5	40,5
P25788	Proteasome subunit alpha type-3	PSMA3	-0,52	0,70	5,44E-04	6	22,4
P13073	Cytochrome c oxidase subunit 4 isoform 1, mitochondrial	COX4I1	-0,50	0,71	7,07E-03	6	31,4
Q9BRA2	Thioredoxin domain-containing protein 17	TXNDC17	-0,50	0,71	3,68E-03	4	46,3
P38159	RNA-binding motif protein, X chromosome	RBMX	-0,50	0,71	2,35E-04	10	26,1
P48643	T-complex protein 1 subunit epsilon	CCT5	0,50	1,41	6,28E-04	28	68,6
P12532	Creatine kinase U-type, mitochondrial	CKMT1A	0,50	1,41	6,28E-04	7	35,5
P04844	Dolichyl-diphosphooligosaccharide--protein glycosyltransferase subunit 2	RPN2	0,50	1,41	1,71E-04	16	39,9
Q9Y5L0	Transportin-3	TNPO3	0,50	1,41	4,25E-02	6	11,1
P63208	S-phase kinase-associated protein 1	SKP1	0,50	1,41	7,76E-03	5	33,7
P06748	Nucleophosmin	NPM1	0,51	1,42	3,58E-03	11	36,4
O15160	DNA-directed RNA polymerases I and III subunit RPAC1	POLR1C	0,52	1,43	3,37E-03	5	21,7
Q9Y285	Phenylalanine--tRNA ligase alpha subunit	FARSA	0,52	1,44	1,69E-02	6	19,5
P23921	Ribonucleoside-diphosphate reductase large subunit	RRM1	0,53	1,44	8,05E-03	8	19,4
Q13126	S-methyl-5-thioadenosine phosphorylase	MTAP	0,55	1,46	3,11E-03	8	48,1
Q9BSJ8	Extended synaptotagmin-1	ESYT1	0,55	1,47	1,37E-02	5	6,4
Q15435	Protein phosphatase 1 regulatory subunit 7	PPP1R7	0,55	1,47	4,65E-03	6	23,9
Q92616	eIF-2-alpha kinase activator GCN1	GCN1	0,55	1,47	1,74E-04	35	21

P24534	Elongation factor 1-beta	EEF1B2	0,56	1,47	2,75E-03	5	37,3
P09104	Gamma-enolase	ENO2	0,56	1,47	2,35E-04	13	48,6
Q14257	Reticulocalbin-2	RCN2	0,57	1,49	6,68E-03	4	20,8
P62917	60S ribosomal protein L8	RPL8	0,58	1,50	9,79E-03	7	33,9
Q8IY67	Ribonucleoprotein PTB-binding 1	RAVER1	0,58	1,50	2,35E-04	5	17,3
Q8IZL8	Proline-, glutamic acid- and leucine-rich protein 1	PELP1	0,59	1,51	3,75E-03	6	9,4
P61011	Signal recognition particle 54 kDa protein	SRP54	0,60	1,51	2,37E-02	7	21
Q9UBE0	SUMO-activating enzyme subunit 1	SAE1	0,60	1,52	4,37E-04	11	47,1
Q6P1J9	Parafibromin	CDC73	0,60	1,52	2,70E-02	4	9,6
Q9ULC4	Malignant T-cell-amplified sequence 1	MCTS1	0,62	1,54	3,58E-03	7	57,5
P47985	Cytochrome b-c1 complex subunit Rieske, mitochondrial	UQCRFS1	0,62	1,54	3,37E-03	4	22,6
Q8N3U4	Cohesin subunit SA-2	STAG2	0,63	1,55	1,48E-02	7	8,1
P26038	Moesin	MSN	0,63	1,55	5,64E-03	7	15,8
P60953	Cell division control protein 42 homolog	CDC42	0,63	1,55	7,15E-03	8	48,2
Q15021	Condensin complex subunit 1	NCAPD2	0,64	1,55	3,53E-03	14	16,2
Q96P70	Importin-9	IPO9	0,66	1,58	1,03E-02	5	9,1
P26599	Polypyrimidine tract-binding protein 1	PTBP1	0,66	1,59	2,24E-04	16	52
Q9C0B1	Alpha-ketoglutarate-dependent dioxygenase FTO	FTO	0,67	1,59	6,56E-03	7	20,4
Q92896	Golgi apparatus protein 1	GLG1	0,69	1,61	2,52E-02	4	4,8
Q96CS3	FAS-associated factor 2	FAF2	0,69	1,62	2,35E-04	4	16,2
P61970	Nuclear transport factor 2	NUTF2	0,70	1,62	3,58E-03	5	69,3
P63010	AP-2 complex subunit beta	AP2B1	0,70	1,63	2,39E-04	10	20,4
P04632	Calpain small subunit 1	CAPNS1	0,71	1,63	3,81E-02	5	31
Q9Y6G9	Cytoplasmic dynein 1 light intermediate chain 1	DYNC1LI1	0,73	1,65	2,07E-03	5	18,4
Q9HAV4	Exportin-5	XPO5	0,73	1,65	2,21E-03	13	15,9
O75947	ATP synthase subunit d, mitochondrial	ATP5PD	0,73	1,66	1,71E-03	8	64
P08708	40S ribosomal protein S17	RPS17	0,74	1,67	9,90E-04	8	60
P20290	Transcription factor BTF3	BTF3	0,75	1,68	1,20E-03	6	48,1
Q96AC1	Fermitin family homolog 2	FERMT2	0,75	1,68	1,48E-02	8	18,4
Q5JTH9	RRP12-like protein	RRP12	0,79	1,72	4,43E-02	11	12,8
Q9H0A0	RNA cytidine acetyltransferase	NAT10	0,79	1,73	5,44E-04	12	17,2
Q96T76	MMS19 nucleotide excision repair protein homolog	MMS19	0,80	1,75	5,44E-04	6	10,2
P62081	40S ribosomal protein S7	RPS7	0,82	1,76	2,31E-03	12	55,7
O00429	Dynamin-1-like protein	DNM1L	0,82	1,77	1,42E-03	12	27,3
P35270	Sepiapterin reductase	SPR	0,87	1,83	1,81E-02	4	24,5
O00764	Pyridoxal kinase	PDXK	0,87	1,83	1,97E-02	4	21,2
O15260	Surfeit locus protein 4	SURF4	0,88	1,83	1,82E-03	4	18,2
Q9BXW7	Haloacid dehalogenase-like hydrolase domain-containing 5	HDHD5	0,92	1,89	2,99E-03	6	25,1
P61086	Ubiquitin-conjugating enzyme E2 K	UBE2K	0,92	1,90	3,36E-02	4	33
P99999	Cytochrome c	CYCS	0,97	1,95	6,59E-04	4	41
Q14978	Nucleolar and coiled-body phosphoprotein 1	NOLC1	1,01	2,01	2,14E-02	8	15,2
Q99436	Proteasome subunit beta type-7	PSMB7	1,01	2,02	7,16E-03	6	34,3
Q9GZS3	WD repeat-containing protein 61	WDR61	1,02	2,02	2,21E-03	4	26,9
Q9BWD1	Acetyl-CoA acetyltransferase, cytosolic	ACAT2	1,03	2,04	2,07E-04	5	35
Q05639	Elongation factor 1-alpha 2	EEF1A2	1,04	2,05	8,77E-04	8	38,2
Q14011	Cold-inducible RNA-binding protein	CIRBP	1,06	2,09	4,55E-04	4	32
Q16836	Hydroxyacyl-coenzyme A dehydrogenase, mitochondrial	HADH	1,07	2,09	4,26E-04	8	42,4
Q8N684	Cleavage and polyadenylation specificity factor subunit 7	CPSF7	1,08	2,11	2,71E-02	4	13,4
Q99598	Translin-associated protein X	TSNAX	1,11	2,16	7,56E-03	4	22,8
Q15428	Splicing factor 3A subunit 2	SF3A2	1,11	2,17	4,19E-02	7	24,4
P25787	Proteasome subunit alpha type-2	PSMA2	1,12	2,17	1,71E-04	7	45,7
P07602	Prosaposin	PSAP	1,31	2,47	2,35E-02	4	9,2
Q7L1Q6	Basic leucine zipper and W2 domain-containing protein 1	BZW1	2,10	4,28	1,76E-03	8	23,4

Appendix table S7. Proteins that take part in the interaction-network, according to those downregulated by compound #7 in HEK293T-ACE2 cells.

A protein interaction network was generated using the Search Tool for the Retrieval of Interacting Genes/ Proteins (STRING) database (https://string-db.org), using only those proteins that were downregulated in PI-7- treated cells. Data given include term ID, term description, observed gene count in the network, background gene count, strength, false discovery rate (FDR), and matching network proteins.

Term ID	Term description	Observed gene count	Background gene count	Strength	FDR	Matching proteins in the network
GO:0070972	Protein localization to endoplasmic reticulum	8	140	1.79	4.64e-09	CHMP4B,RPS11,RPS27A,RPS27,RPL23A,BCAP31,RPS20,RPL18
GO:0045047	Protein targeting to endoplasmic reticulum	7	110	1.84	4.39e-08	CHMP4B,RPS11,RPS27A,RPS27,RPL23A,RPS20,RPL18
GO:0006413	Translational initiation	7	141	1.73	1.16e-07	RPS11,RPS27A,RPS27,EIF1AX,RPL23A,RPS20,RPL18
GO:0006612	Protein targeting to membrane	7	165	1.66	2.69e-07	CHMP4B,RPS11,RPS27A,RPS27,RPL23A,RPS20,RPL18
GO:0006614	SRP-dependent cotranslational protein targeting to membrane	6	96	1.83	6.50e-07	RPS11,RPS27A,RPS27,RPL23A,RPS20,RPL18
GO:0019083	Viral transcription	6	115	1.75	1.38e-06	RPS11,RPS27A,RPS27,RPL23A,RPS20,RPL18
GO:0000184	Nuclear-transcribed mRNA catabolic process, nonsense-mediated decay	6	119	1.74	1.49e-06	RPS11,RPS27A,RPS27,RPL23A,RPS20,RPL18
GO:0072657	Protein localization to membrane	8	495	1.24	6.70e-06	CHMP4B,RPS11,RPS27A,BSG,RPS27,RPL23A,RPS20,RPL18
GO:0016032	Viral process	9	776	1.1	8.86e-06	PSMA3,CHMP4B,RPS11,RPS27A,RPS27,RPL23A,BCAP31,RPS20,RPL18
GO:0044265	Cellular macromolecule catabolic process	8	917	0.98	0.00035	PSMA3,CHMP4B,RPS11,RPS27A,RPS27,RPL23A,RPS20,RPL18
GO:0006886	Intracellular protein transport	8	999	0.94	0.00062	CHMP4B,RPS11,RPS27A,RPS27,RPL23A,BCAP31,RPS20,RPL18
GO:0016071	mRNA metabolic process	7	678	1.05	0.00065	RPS11,RPS27A,RPS27,RBMX,RPL23A,RPS20,RPL18
GO:0034613	Cellular protein localization	9	1610	0.78	0.0016	CHMP4B,RPS11,RPS27A,BSG,RPS27,RPL23A,BCAP31,RPS20,RPL18
GO:0044271	Cellular nitrogen compound biosynthetic process	8	1522	0.76	0.0095	RPS11,RPS27A,RPS27,RBMX,EIF1AX,RPL23A,RPS20,RPL18
GO:0034645	Cellular macromolecule biosynthetic process	8	1592	0.74	0.0118	RPS11,RPS27A,RPS27,RBMX,EIF1AX,RPL23A,RPS20,RPL18
GO:0008152	Metabolic process	16	8298	0.32	0.0167	PSMA3,CHMP4B,TXNDC17,RPS11,RPS27A,HIST1H1E,BSG,RPS27,RBMX,EIF1AX,CIAPIN1,RPL23A,RPS20,RPL18,ERH,COX4I1
GO:0034622	Cellular protein-containing complex assembly	6	816	0.9	0.0173	CHMP4B,RPS27A,HIST1H1E,RPS27,RBMX,RPL23A
GO:0051641	Cellular localization	10	2967	0.56	0.0220	CHMP4B,RPS11,RPS27A,BSG,RPS27,RPL23A,BCAP31,RPS20,MANF,RPL18
GO:0051649	Establishment of localization in cell	9	2375	0.61	0.0247	CHMP4B,RPS11,RPS27A,RPS27,RPL23A,BCAP31,RPS20,MANF,RPL18
GO:0044237	Cellular metabolic process	15	7513	0.34	0.0294	PSMA3,CHMP4B,RPS11,RPS27A,HIST1H1E,BSG,RPS27,RBMX,EIF1AX,CIAPIN1,RPL23A,RPS20,RPL18,ERH,COX4I1
GO:0010629	Negative regulation of gene expression	8	2014	0.64	0.0485	RPS11,RPS27A,HIST1H1E,RPS27,RBMX,RPL23A,RPS20,RPL18

Appendix table S8. Proteins that take part in the interaction network, according to those upregulated by compound #7 in HEK293T-ACE2 cells.

A protein interaction network was generated using STRING database (https://string-db.org) by using only those proteins found upregulated in PI-7-treated cells. Data given include term ID, term description, observed gene count in the network, background gene count, strength, false discovery rate (FDR), and matching network proteins.

Term ID	Term description	Observed gene count	Background gene count	Strength	FDR	Matching proteins in the network
GO:0034641	Cellular nitrogen compound metabolic process	32	3282	0.46	3.69e-05	EEF1A2,SF3A2,SPR,NAT10,RPL8,XPO5,WDR61,PDXK,RAVER1,NPM1,GCN1L1,RRM1,PELP1,ATP5H,FARSA,RPS7,CPSF7,RPS17,PTBP1,TSNAX,CDC73,MCTS1,POLR1C,MTAP,BTF3,EEF1B2,PSAP,NOLC1,MMS19,FTO,ENO2,RRP12
GO:0010608	Posttranscriptional regulation of gene expression	14	574	0.86	4.75e-05	EEF1A2,PSMA2,NAT10,PSMB7,XPO5,NPM1,GCN1L1,PTBP1,TSNAX,MCTS1,EEF1B2,NOLC1,FTO,CIRBP
GO:0044237	Cellular metabolic process	48	7513	0.28	7.28e-05	EEF1A2,SF3A2,PSMA2,SKP1,SPR,RPN2,NAT10,PSMB7,UBE2K,FAF2,RPL8,XPO5,ESYT1,WDR61,SAE1,PDXK,RAVER1,NPM1,GCN1L1,RRM1,PELP1,ATP5H,UQCRFS1,CYCS,FARSA,CECR5,RPS7,CPSF7,RPS17,PTBP1,TSNAX,ACAT2,CDC73,MCTS1,POLR1C,MTAP,BTF3,EEF1B2,PSAP,CDC42,NOLC1,CKMT1A,MMS19,FTO,ENO2,RRP12,HADH,AP2B1
GO:0006807	Nitrogen compound metabolic process	45	6852	0.29	0.00015	EEF1A2,SF3A2,PSMA2,SKP1,SPR,RPN2,CAPNS1,NAT10,PSMB7,UBE2K,FAF2,RPL8,XPO5,ESYT1,WDR61,SAE1,PDXK,RAVER1,NPM1,GCN1L1,RRM1,PELP1,ATP5H,CYCS,FARSA,RPS7,CPSF7,RPS17,PTBP1,TSNAX,CDC73,MCTS1,POLR1C,MTAP,BTF3,EEF1B2,PSAP,CDC42,NOLC1,CKMT1A,MMS19,FTO,ENO2,RRP12,AP2B1
GO:0071704	Organic substance metabolic process	48	7755	0.26	0.00015	EEF1A2,SF3A2,PSMA2,SKP1,SPR,RPN2,CAPNS1,NAT10,PSMB7,UBE2K,FAF2,RPL8,XPO5,ESYT1,WDR61,SAE1,PDXK,RAVER1,NPM1,GCN1L1,RRM1,PELP1,ATP5H,CYCS,FARSA,CECR5,RPS7,CPSF7,RPS17,PTBP1,TSNAX,ACAT2,CDC73,MCTS1,POLR1C,MTAP,BTF3,EEF1B2,PSAP,CDC42,NOLC1,CKMT1A,MMS19,FTO,ENO2,RRP12,HADH,AP2B1
GO:0010467	Gene expression	23	2056	0.52	0.00027	EEF1A2,SF3A2,NAT10,RPL8,WDR61,RAVER1,GCN1L1,PELP1,FARSA,RPS7,CPSF7,RPS17,PTBP1,TSNAX,CDC73,MCTS1,POLR1C,BTF3,EEF1B2,PSAP,NOLC1,MMS19,RRP12
GO:0006725	Cellular aromatic compound metabolic process	27	2882	0.44	0.00030	SF3A2,SPR,NAT10,RPL8,XPO5,WDR61,PDXK,RAVER1,NPM1,RRM1,PELP1,ATP5H,FARSA,RPS7,CPSF7,RPS17,PTBP1,TSNAX,CDC73,POLR1C,MTAP,BTF3,NOLC1,MMS19,FTO,ENO2,RRP12
GO:0008152	Metabolic process	49	8298	0.24	0.00030	EEF1A2,SF3A2,PSMA2,SKP1,SPR,RPN2,CAPNS1,NAT10,PSMB7,UBE2K,FAF2,RPL8,XPO5,ESYT1,WDR61,SAE1,PDXK,RAVER1,NPM1,GCN1L1,RRM1,PELP1,ATP5H,UQCRFS1,CYCS,FARSA,CECR5,RPS7,CPSF7,RPS17,PTBP1,TSNAX,ACAT2,CDC73,MCTS1,POLR1C,MTAP,BTF3,EEF1B2,PSAP,CDC42,NOLC1,CKMT1A,MMS19,FTO,ENO2,RRP12,HADH,AP2B1
GO:0016070	RNA metabolic process	20	1584	0.57	0.00030	SF3A2,NAT10,RPL8,XPO5,WDR61,RAVER1,PELP1,FARSA,RPS7,CPSF7,RPS17,PTBP1,TSNAX,CDC73,POLR1C,BTF3,NOLC1,MMS19,FTO,RRP12
GO:0022613	Ribonucleoprotein complex biogenesis	11	423	0.89	0.00030	SF3A2,NAT10,NPM1,PELP1,RPS7,CPSF7,RPS17,CDC73,MCTS1,NOLC1,RRP12
GO:0046483	Heterocycle metabolic process	27	2840	0.45	0.00030	SF3A2,SPR,NAT10,RPL8,XPO5,WDR61,PDXK,RAVER1,NPM1,RRM1,PELP1,ATP5H,FARSA,R

						PS7,CPSF7,RPS17,PTBP1,TSNAX,CDC73,POLR1C,MTAP,BTF3,NOLC1,MMS19,FTO,ENO2,RRP12
GO:1901360	Organic cyclic compound metabolic process	28	3118	0.43	0.00033	SF3A2,SPR,NAT10,RPL8,XPO5,WDR61,PDXK,RAVER1,NPM1,RRM1,PELP1,ATP5H,FARSA,RPS7,CPSF7,RPS17,PTBP1,TSNAX,ACAT2,CDC73,POLR1C,MTAP,BTF3,NOLC1,MMS19,FTO,ENO2,RRP12
GO:0044238	Primary metabolic process	45	7332	0.26	0.00043	EEF1A2,SF3A2,PSMA2,SKP1,RPN2,CAPNS1,NAT10,PSMB7,UBE2K,FAF2,RPL8,XPO5,ESYT1,WDR61,SAE1,RAVER1,NPM1,GCN1L1,RRM1,PELP1,ATP5H,CYCS,FARSA,CECR5,RPS7,CPSF7,RPS17,PTBP1,TSNAX,ACAT2,CDC73,MCTS1,POLR1C,MTAP,BTF3,EEF1B2,PSAP,CDC42,NOLC1,MMS19,FTO,ENO2,RRP12,HADH,AP2B1
GO:0044271	Cellular nitrogen compound biosynthetic process	19	1522	0.57	0.00043	EEF1A2,SPR,RPL8,WDR61,PDXK,GCN1L1,RRM1,ATP5H,FARSA,RPS7,CPSF7,RPS17,CDC73,MCTS1,POLR1C,MTAP,BTF3,EEF1B2,MMS19
GO:0006139	Nucleobase-containing compound metabolic process	25	2659	0.45	0.00068	SF3A2,NAT10,RPL8,XPO5,WDR61,RAVER1,NPM1,RRM1,PELP1,ATP5H,FARSA,RPS7,CPSF7,RPS17,PTBP1,TSNAX,CDC73,POLR1C,MTAP,BTF3,NOLC1,MMS19,FTO,ENO2,RRP12
GO:0006417	Regulation of translation	10	398	0.87	0.00081	EEF1A2,NAT10,NPM1,GCN1L1,PTBP1,MCTS1,EEF1B2,NOLC1,FTO,CIRBP
GO:0016032	Viral process	13	776	0.7	0.0014	SKP1,PSMB7,RPL8,XPO5,DYNC1LI1,NPM1,RPS7,RPS17,PTBP1,MSN,MCTS1,CDC42,AP2B1
GO:0044085	Cellular component biogenesis	24	2583	0.44	0.0014	STAG2,SF3A2,SKP1,NAT10,UBE2K,NPM1,RRM1,PELP1,FARSA,RPS7,FERMT2,CPSF7,RPS17,CDC73,MCTS1,PSAP,CDC42,NOLC1,MMS19,RRP12,DNM1L,SRP54,CIRBP,AP2B1
GO:1901576	Organic substance biosynthetic process	24	2734	0.42	0.0032	EEF1A2,SPR,RPN2,RPL8,WDR61,PDXK,GCN1L1,RRM1,ATP5H,FARSA,CECR5,RPS7,CPSF7,RPS17,ACAT2,CDC73,MCTS1,POLR1C,MTAP,BTF3,EEF1B2,CKMT1A,MMS19,ENO2
GO:0090304	Nucleic acid metabolic process	21	2178	0.46	0.0036	SF3A2,NAT10,RPL8,XPO5,WDR61,RAVER1,NPM1,PELP1,FARSA,RPS7,CPSF7,RPS17,PTBP1,TSNAX,CDC73,POLR1C,BTF3,NOLC1,MMS19,FTO,RRP12
GO:0043170	Macromolecule metabolic process	38	6137	0.26	0.0054	EEF1A2,SF3A2,PSMA2,SKP1,RPN2,CAPNS1,NAT10,PSMB7,UBE2K,FAF2,RPL8,XPO5,WDR61,SAE1,RAVER1,NPM1,GCN1L1,RRM1,PELP1,CYCS,FARSA,RPS7,CPSF7,RPS17,PTBP1,TSNAX,CDC73,MCTS1,POLR1C,BTF3,EEF1B2,PSAP,CDC42,NOLC1,MMS19,FTO,RRP12,AP2B1
GO:1903311	Regulation of mrna metabolic process	8	338	0.85	0.0104	PSMA2,PSMB7,NPM1,CPSF7,PTBP1,CDC73,FTO,CIRBP
GO:0006396	RNA processing	12	854	0.62	0.0130	SF3A2,NAT10,RAVER1,PELP1,RPS7,CPSF7,RPS17,PTBP1,TSNAX,CDC73,NOLC1,RRP12
GO:0016055	Wnt signaling pathway	8	351	0.83	0.0130	PSMA2,SKP1,PSMB7,WDR61,FERMT2,CDC73,CDC42,AP2B1
GO:0051290	Protein heterotetramerization	3	15	1.77	0.0130	RRM1,FARSA,CPSF7
GO:0006412	Translation	8	366	0.81	0.0149	EEF1A2,RPL8,GCN1L1,FARSA,RPS7,RPS17,MCTS1,EEF1B2
GO:0031329	Regulation of cellular catabolic process	12	875	0.61	0.0149	EEF1A2,PSMA2,CAPNS1,PSMB7,UBE2K,NPM1,RPS7,MSN,PSAP,FTO,DNM1L,CIRBP
GO:0046907	Intracellular transport	16	1520	0.49	0.0149	NUTF2,FAF2,RPL8,XPO5,TNPO3,DYNC1LI1,NPM1,ATP5H,RPS7,RPS17,IPO9,PSAP,CDC42,DNM1L,SRP54,AP2B1
GO:0009987	Cellular process	63	15024	0.09	0.0158	GLG1,EEF1A2,STAG2,NUTF2,SF3A2,PSMA2,SKP1,SPR,RPN2,CAPNS1,NAT10,PSMB7,UBE2K,FAF2,RPL8,XPO5,TNPO3,ESYT1,WDR61,SAE1,DYNC1LI1,CCT5,PDXK,RAVER1,NPM1,GCN1L1,RRM1,PELP1,ATP5H,UQCRFS1,CYCS,FARSA,NCAPD2,CECR5,RPS7,FERMT2,CPSF7,RPS17,PTBP1,MSN,IPO9,TSNAX,ACAT2,CDC73,MCTS1,SURF4,POLR1C,MTAP,BTF3,EEF1B2,PSAP,CDC42,NOLC1,CKMT1A,MMS19,FTO,ENO2,RRP12,DNM1L,SRP54,CIRBP,HADH,AP2B1

GO:0032268	Regulation of cellular protein metabolic process	22	2693	0.38	0.0170	GLG1,EEF1A2,SKP1,PPP1R7,NAT10,UBE2K,WDR61,SAE1,NPM1,GCN1L1,CYCS,RPS7,PTBP1,MSN,MCTS1,EEF1B2,PSAP,CDC42,NOLC1,FTO,DNM1L,CIRBP
GO:0034645	Cellular macromolecule biosynthetic process	16	1592	0.47	0.0226	EEF1A2,RPN2,RPL8,WDR61,GCN1L1,RRM1,FARSA,RPS7,CPSF7,RPS17,CDC73,MCTS1,POLR1C,BTF3,EEF1B2,MMS19
GO:0042254	Ribosome biogenesis	7	292	0.85	0.0226	NAT10,NPM1,PELP1,RPS7,RPS17,NOLC1,RRP12
GO:0051641	Cellular localization	23	2967	0.36	0.0226	NUTF2,PSMA2,SKP1,PSMB7,FAF2,RPL8,XPO5,TNPO3,ESYT1,DYNC1LI1,PDXK,NPM1,ATP5H,RPS7,FERMT2,RPS17,IPO9,SURF4,PSAP,CDC42,DNM1L,SRP54,AP2B1
GO:0034613	Cellular protein localization	16	1610	0.47	0.0239	NUTF2,SKP1,FAF2,RPL8,XPO5,TNPO3,NPM1,RPS7,FERMT2,RPS17,IPO9,PSAP,CDC42,DNM1L,SRP54,AP2B1
GO:0051649	Establishment of localization in cell	20	2375	0.4	0.0241	NUTF2,PSMA2,PSMB7,FAF2,RPL8,XPO5,TNPO3,DYNC1LI1,PDXK,NPM1,ATP5H,RPS7,RPS17,IPO9,SURF4,PSAP,CDC42,DNM1L,SRP54,AP2B1
GO:0006364	rRNA processing	6	212	0.92	0.0264	NAT10,PELP1,RPS7,RPS17,NOLC1,RRP12
GO:0019080	Viral gene expression	5	132	1.05	0.0264	RPL8,RPS7,RPS17,PTBP1,MCTS1
GO:0044249	Cellular biosynthetic process	21	2611	0.38	0.0264	EEF1A2,SPR,RPN2,RPL8,WDR61,PDXK,GCN1L1,RRM1,ATP5H,FARSA,CECR5,RPS7,CPSF7,RPS17,CDC73,MCTS1,POLR1C,MTAP,BTF3,EEF1B2,MMS19
GO:0071840	Cellular component organization or biogenesis	34	5633	0.25	0.0264	STAG2,SF3A2,SKP1,CAPNS1,NAT10,UBE2K,FAF2,ESYT1,WDR61,DYNC1LI1,NPM1,RRM1,PELP1,ATP5H,CYCS,FARSA,NCAPD2,RPS7,FERMT2,CPSF7,RPS17,MSN,CDC73,MCTS1,SURF4,PSAP,CDC42,NOLC1,MMS19,RRP12,DNM1L,SRP54,CIRBP,AP2B1
GO:0042274	Ribosomal small subunit biogenesis	4	70	1.23	0.0293	NAT10,NPM1,RPS7,RPS17
GO:0072594	Establishment of protein localization to organelle	8	433	0.74	0.0293	NUTF2,RPL8,TNPO3,RPS7,RPS17,IPO9,SRP54,AP2B1
GO:0006886	Intracellular protein transport	12	999	0.55	0.0301	NUTF2,FAF2,RPL8,XPO5,TNPO3,NPM1,RPS7,RPS17,IPO9,PSAP,SRP54,AP2B1
GO:1904666	Regulation of ubiquitin protein ligase activity	3	26	1.53	0.0302	SKP1,RPS7,DNM1L
GO:1901566	Organonitrogen compound biosynthetic process	14	1346	0.49	0.0334	EEF1A2,SPR,RPN2,RPL8,PDXK,GCN1L1,ATP5H,FARSA,RPS7,RPS17,MCTS1,MTAP,EEF1B2,CKMT1A
GO:0034660	ncRNA metabolic process	8	467	0.71	0.0419	NAT10,XPO5,PELP1,FARSA,RPS7,RPS17,NOLC1,RRP12
GO:0048522	Positive regulation of cellular process	33	5579	0.24	0.0423	EEF1A2,NUTF2,SF3A2,PSMA2,SKP1,PPP1R7,CAPNS1,NAT10,PSMB7,UBE2K,XPO5,WDR61,SAE1,DYNC1LI1,CCT5,NPM1,GCN1L1,PELP1,CYCS,RPS7,PTBP1,MSN,CDC73,MCTS1,SURF4,PSAP,CDC42,NOLC1,MMS19,FTO,DNM1L,CIRBP,AP2B1
GO:0033365	Protein localization to organelle	10	743	0.6	0.0427	NUTF2,SKP1,RPL8,TNPO3,RPS7,RPS17,IPO9,DNM1L,SRP54,AP2B1
GO:0048518	Positive regulation of biological process	35	6112	0.23	0.0427	EEF1A2,NUTF2,SF3A2,PSMA2,SKP1,PPP1R7,CAPNS1,NAT10,PSMB7,UBE2K,XPO5,WDR61,SAE1,DYNC1LI1,CCT5,NPM1,GCN1L1,PELP1,CYCS,RPS7,PTBP1,MSN,CDC73,MCTS1,SURF4,POLR1C,PSAP,CDC42,NOLC1,MMS19,FTO,DNM1L,CIRBP,HADH,AP2B1
GO:0019222	Regulation of metabolic process	38	6948	0.21	0.0453	GLG1,EEF1A2,NUTF2,PSMA2,SKP1,PPP1R7,SPR,CAPNS1,NAT10,PSMB7,UBE2K,RPL8,XPO5,WDR61,SAE1,CCT5,NPM1,GCN1L1,PELP1,CYCS,RPS7,CPSF7,RPS17,PTBP1,MSN,TSNAX,CDC73,MCTS1,POLR1C,EEF1B2,PSAP,CDC42,NOLC1,MMS19,FTO,DNM1L,CIRBP,HADH

Appendix table S9

Sequences of the primers used in this study for qPCR analyses.

**CoV-2 N Forward:**	GACCCCAAAATCAGCGAAAT
**CoV-2 N Reverse:**	TCTGGTTACTGCCAGTTGAATCTG
**ACTB Forward:**	GACCCAGATCATGTTTGAGACCTT
**ACTB Reverse:**	CCAGAGGCGTACAGGGATAGC
**IL-1β Forward:**	ATGATGGCTTATTACAGTGGCAA
**IL-1β Reverse:**	GTCGGAGATTCGTAGCTGGA
**TNFA Forward:**	CTCTTCTGCCTGCTGCACTTTG
**TNFA Reverse:**	ATGGGCTACAGGCTTGTCACTC
**ATP2B1 Forward:**	CGATCTCACTGGCTTATTCAGTCA
**ATP2B1 Reverse:**	TAGCTGTAGCATTTCCCATGGTT
**ATP2A1 Forward:**	AACGAGGCCAAAGGTGTCTA
**ATP2A1 Reverse:**	TCGAGAGGCTTCTCACATC
**IL-12 Forward:**	TGATGGCCCTGTGCCTTAGT
**IL-12 Reverse:**	GGATCCATCAGAAGCTTTGCAT
**ACE2 Forward:**	GAAATTCCCAAAGACCAGTGGA
**ACE2 Reverse:**	CCCCAACTATCTCTCGCTTCAT
**Spike Forward:**	ATTGCCACTAGTCTCTAGT
**Spike Reverse:**	AGGATCTGAAAACTTTGTCA
**TMEM16 Forward:**	GAAGCGGAAACAGATGCGACTC
**TMEM16 Reverse:**	CTGGCTTCGTATTCAGCTCTAGG
