# Supplementary figures and images for: Targeting ATP2B1 impairs PI3K/Akt/FOXO signaling and reduces SARS-COV-2 infection and replication
Source: EMBO Rep. 2024 May 30;25(7):12. doi: 10.1038/s44319-024-00164-z (PMC11239940; doi:10.1038/s44319-024-00164-z)

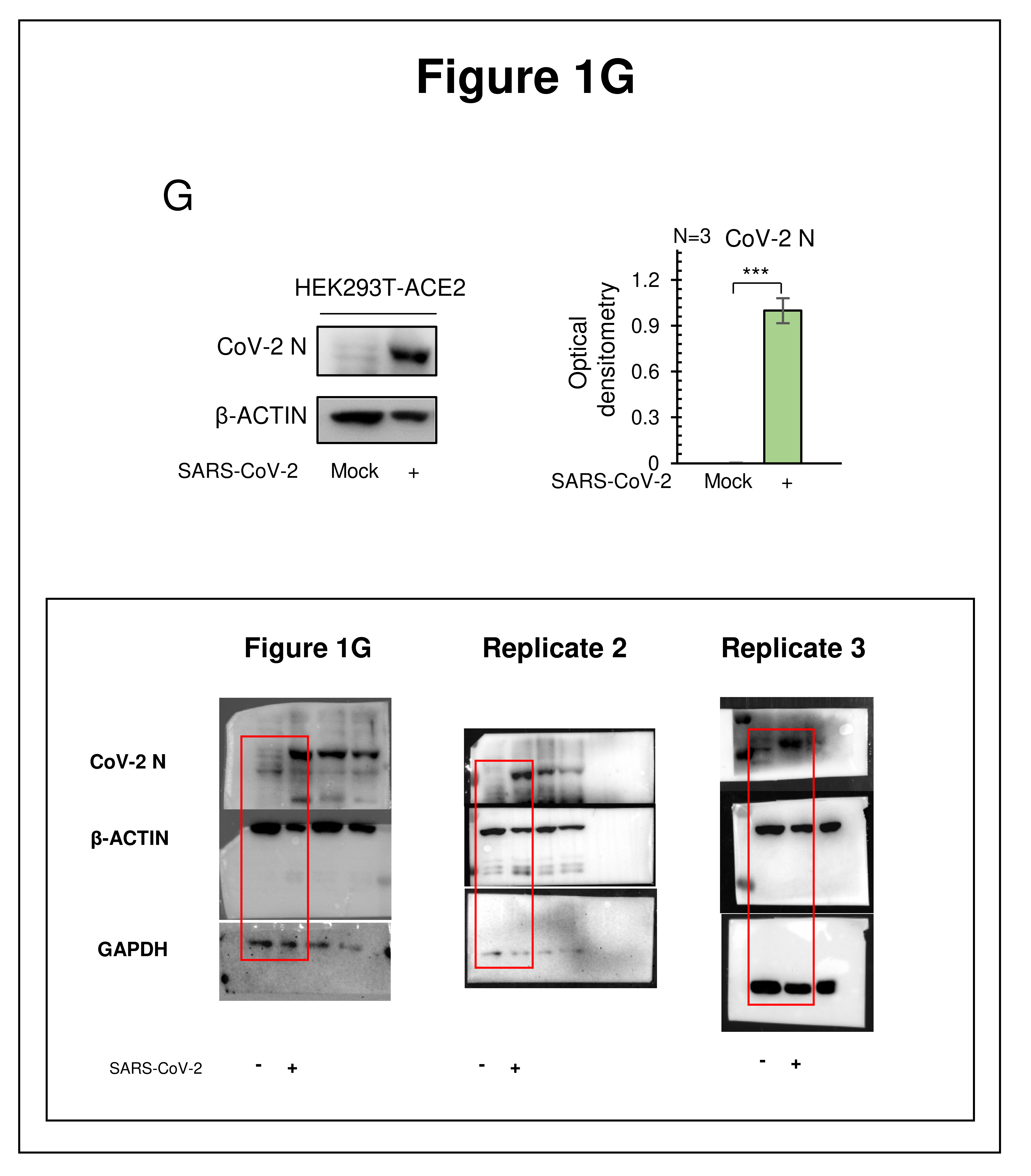

Supplement: Supplementary file 3 — Source data Fig. 1 [file 44319_2024_164_MOESM3_ESM.zip › Figure1/Figure1G/Figure1G.tif]

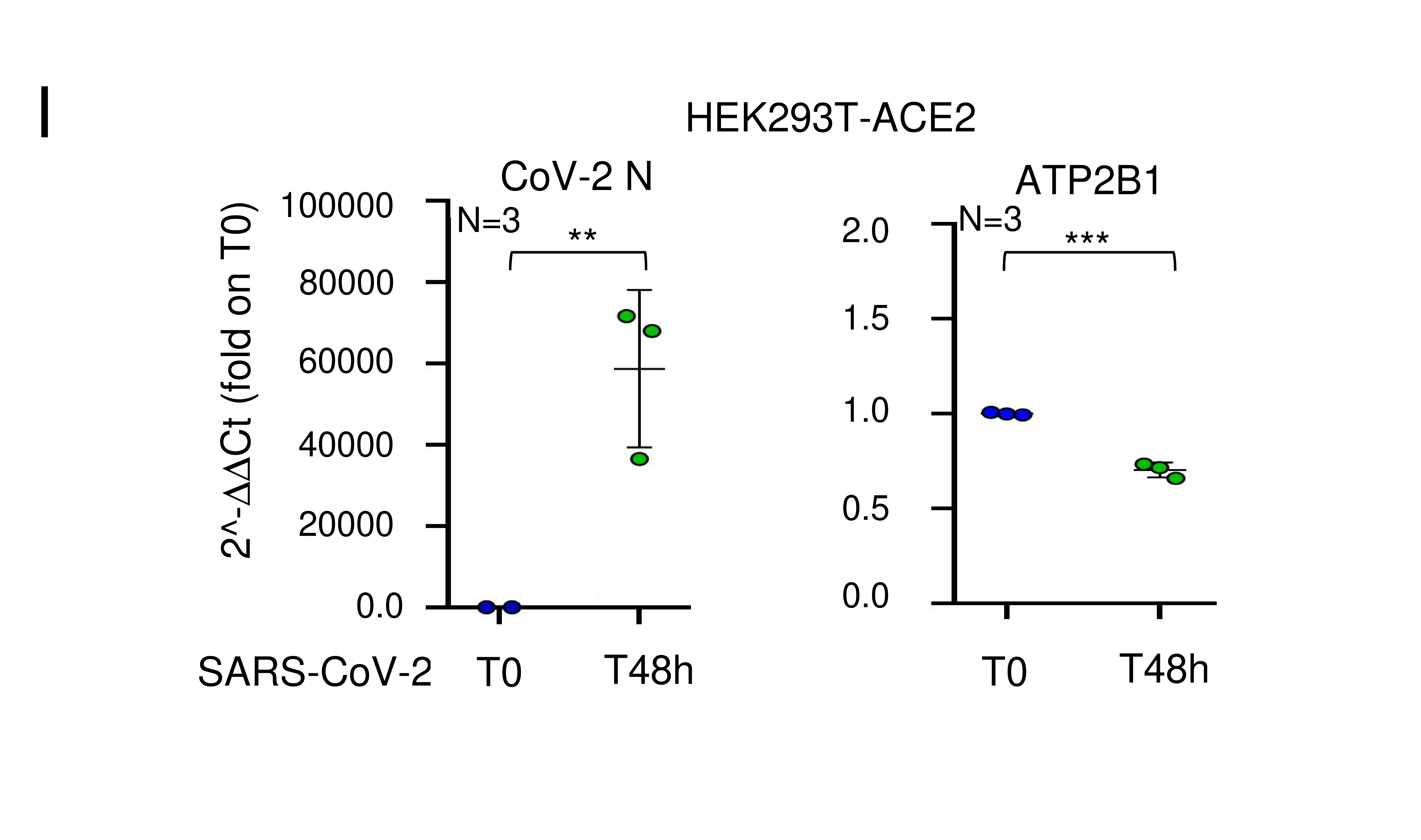

Supplement: Supplementary file 3 — Source data Fig. 1 [file 44319_2024_164_MOESM3_ESM.zip › Figure1/Figure1I/Figure1I.tif]

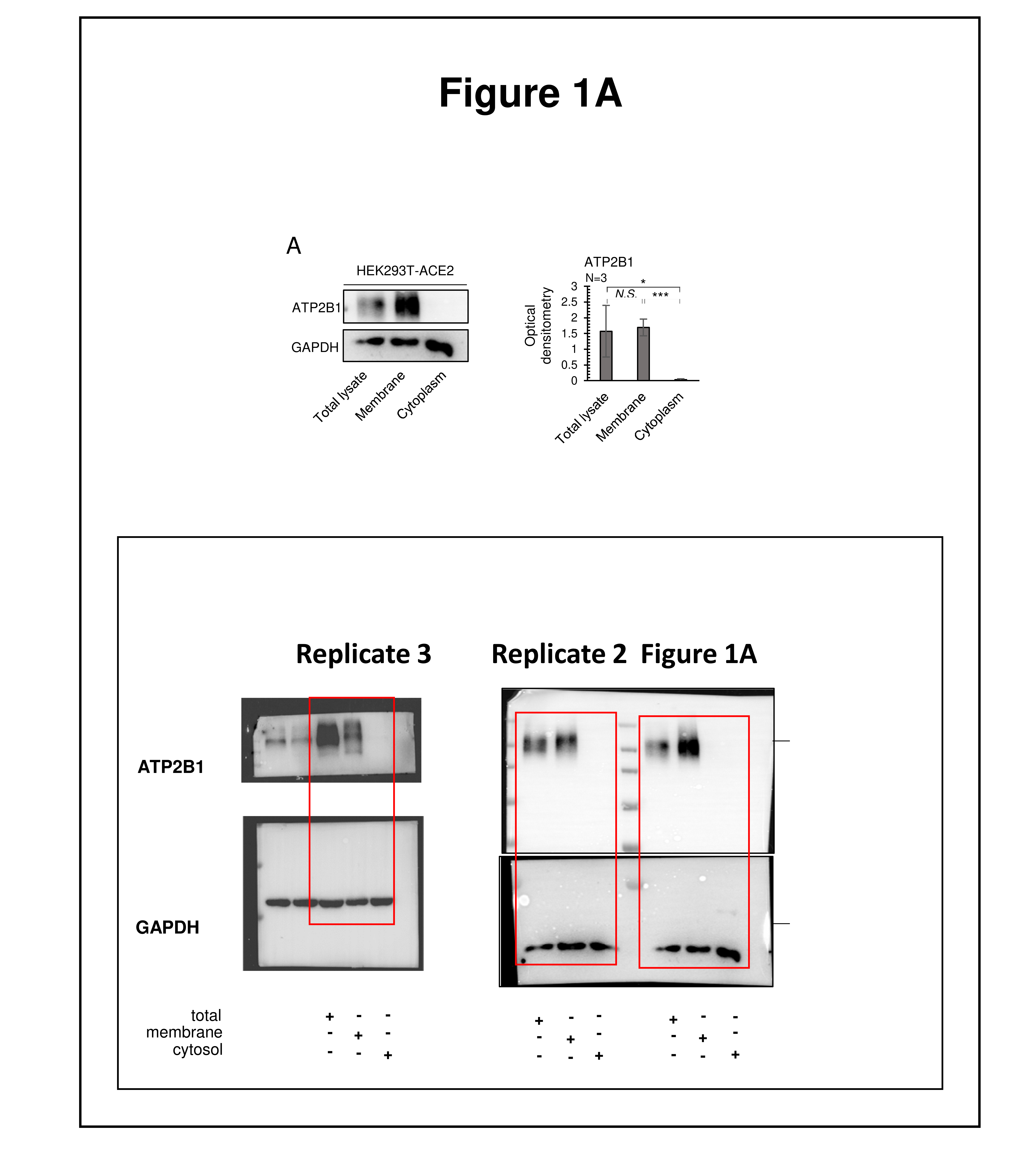

Supplement: Supplementary file 3 — Source data Fig. 1 [file 44319_2024_164_MOESM3_ESM.zip › Figure1/Figure1A/Figure1A.tif]

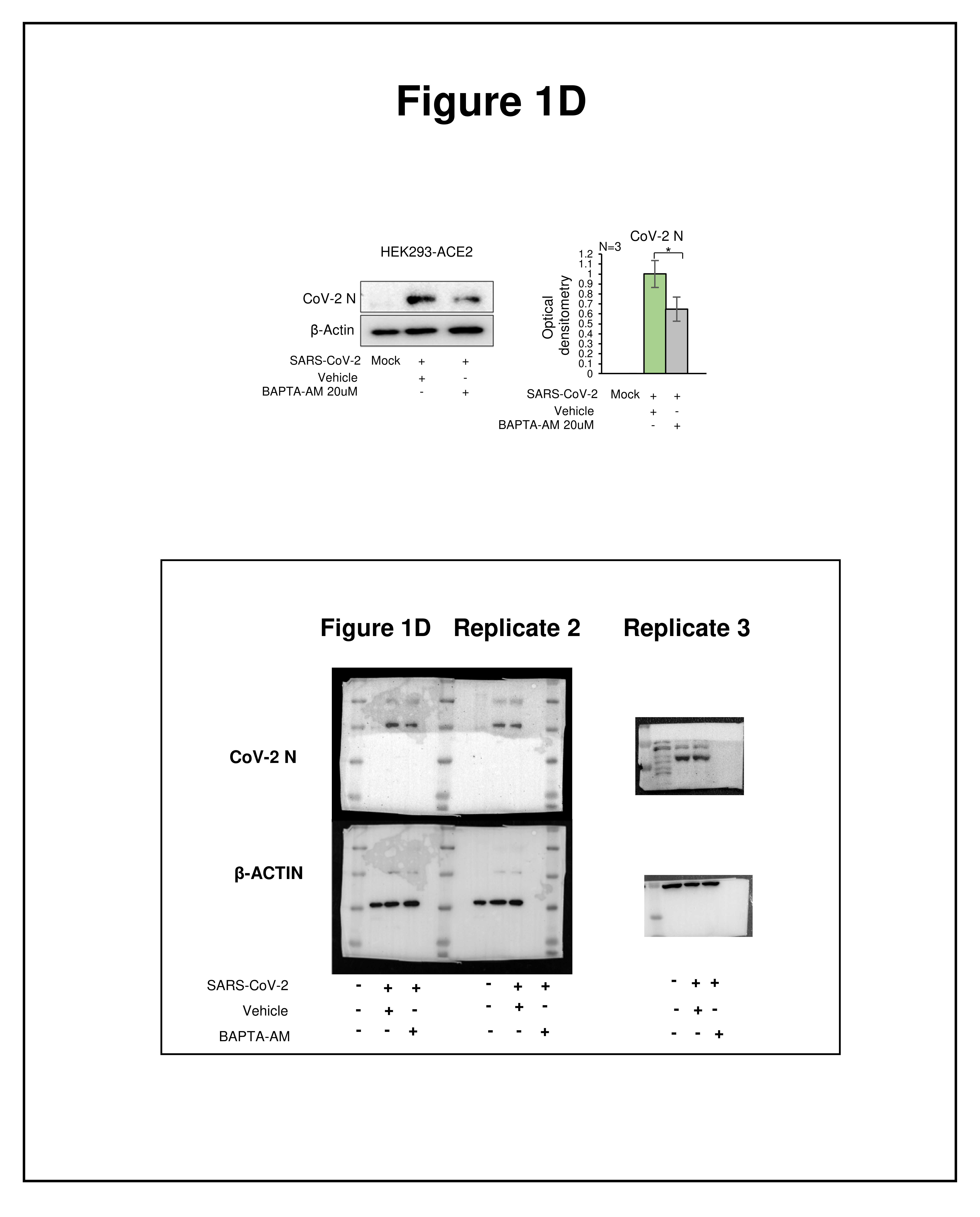

Supplement: Supplementary file 3 — Source data Fig. 1 [file 44319_2024_164_MOESM3_ESM.zip › Figure1/Figure1D/Figure1D.tif]

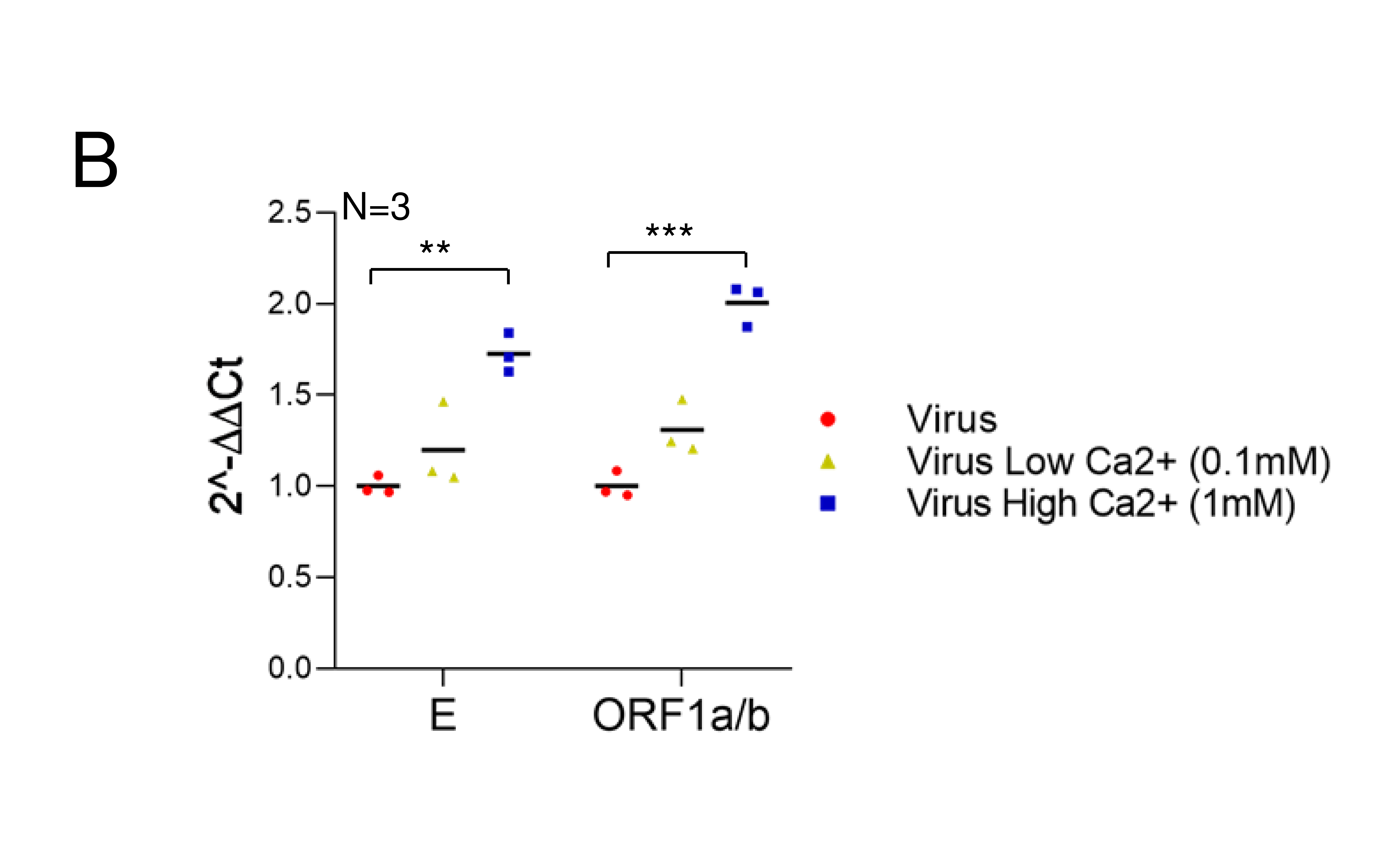

Supplement: Supplementary file 3 — Source data Fig. 1 [file 44319_2024_164_MOESM3_ESM.zip › Figure1/Figure1B/Figure1B.tif]

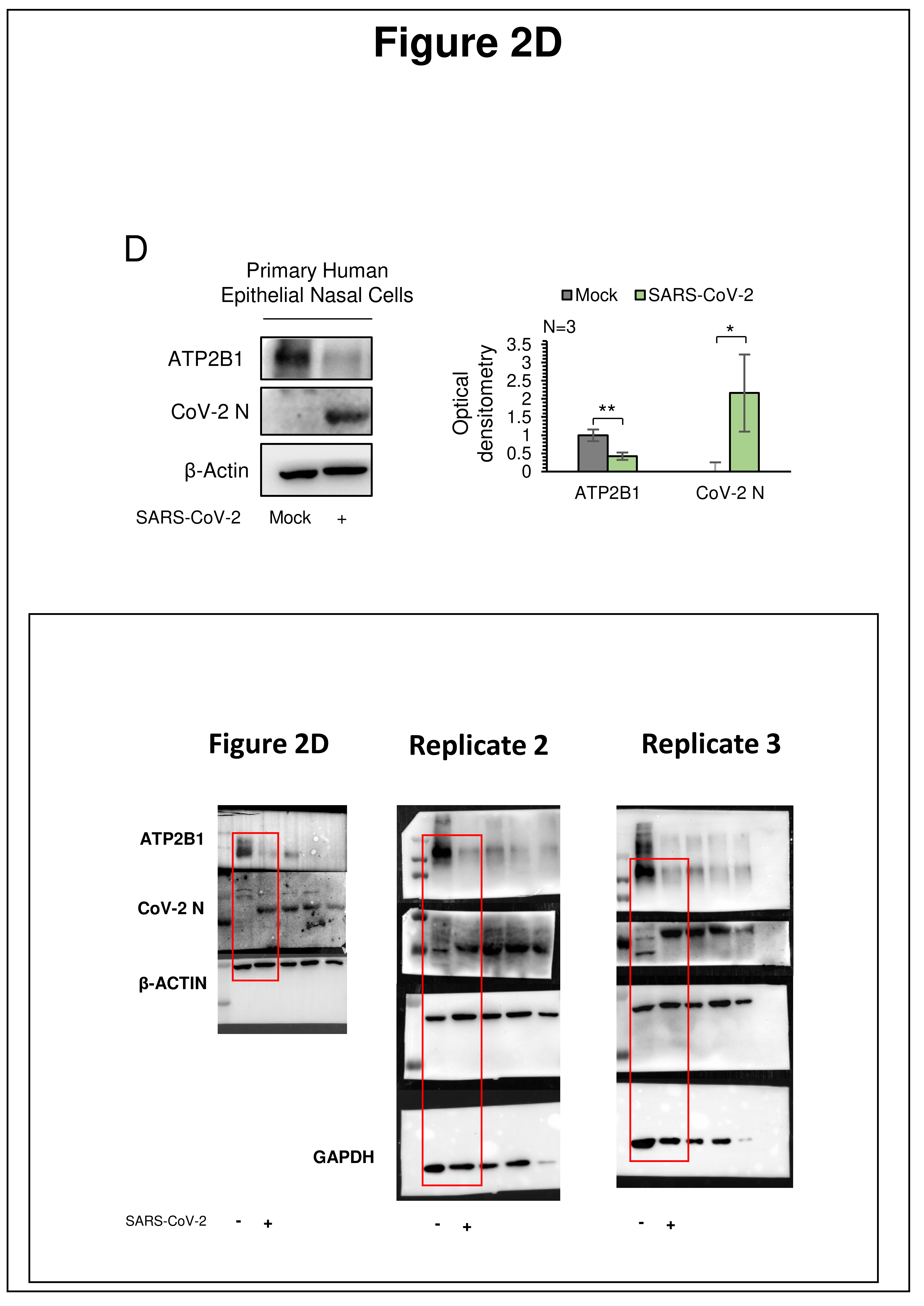

Supplement: Supplementary file 4 — Source data Fig. 2 [file 44319_2024_164_MOESM4_ESM.zip › Figure2/Figure2D-EX2C/Figure2D.tif]

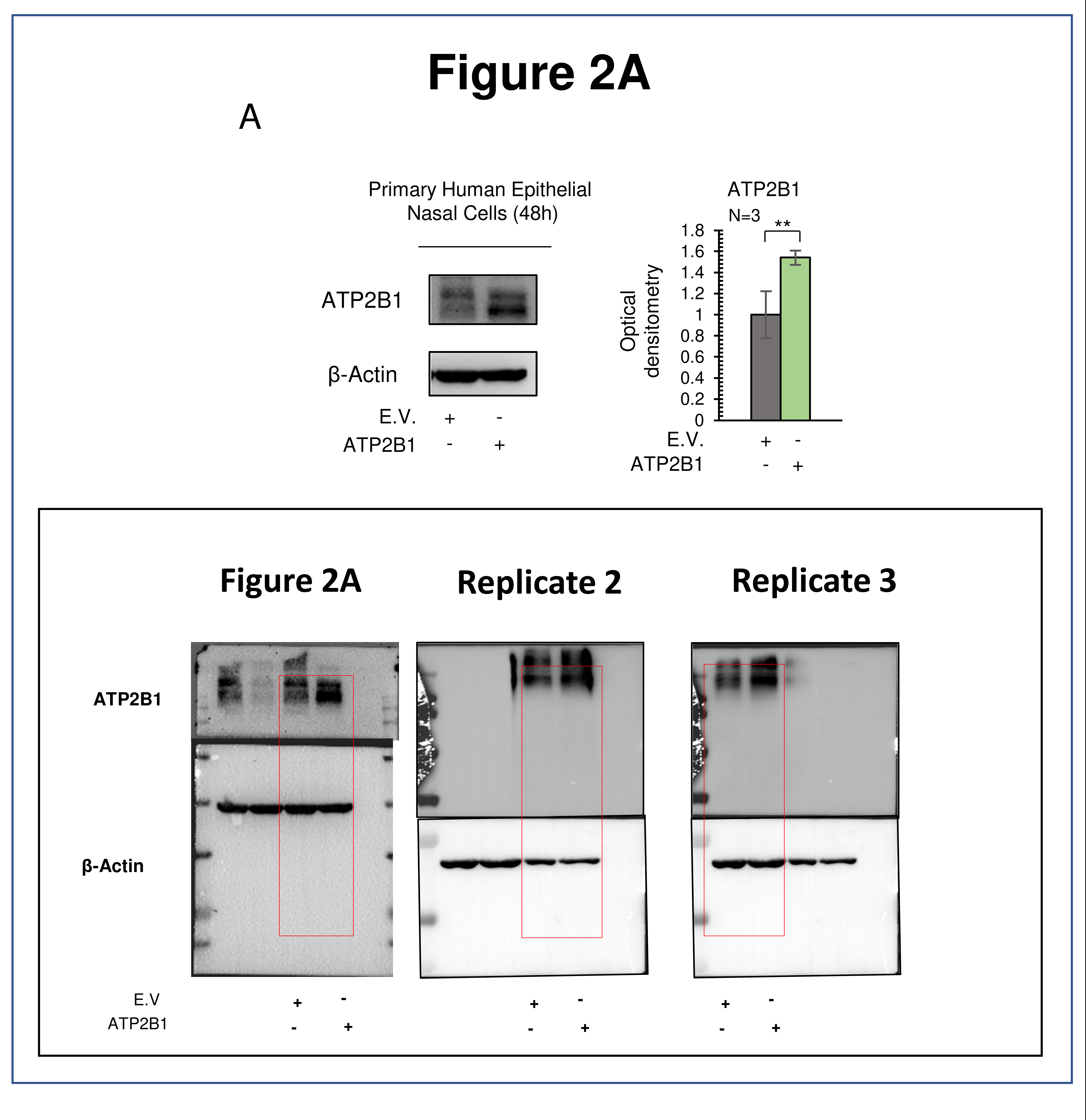

Supplement: Supplementary file 4 — Source data Fig. 2 [file 44319_2024_164_MOESM4_ESM.zip › Figure2/Figure2A/Figure2A.tif]

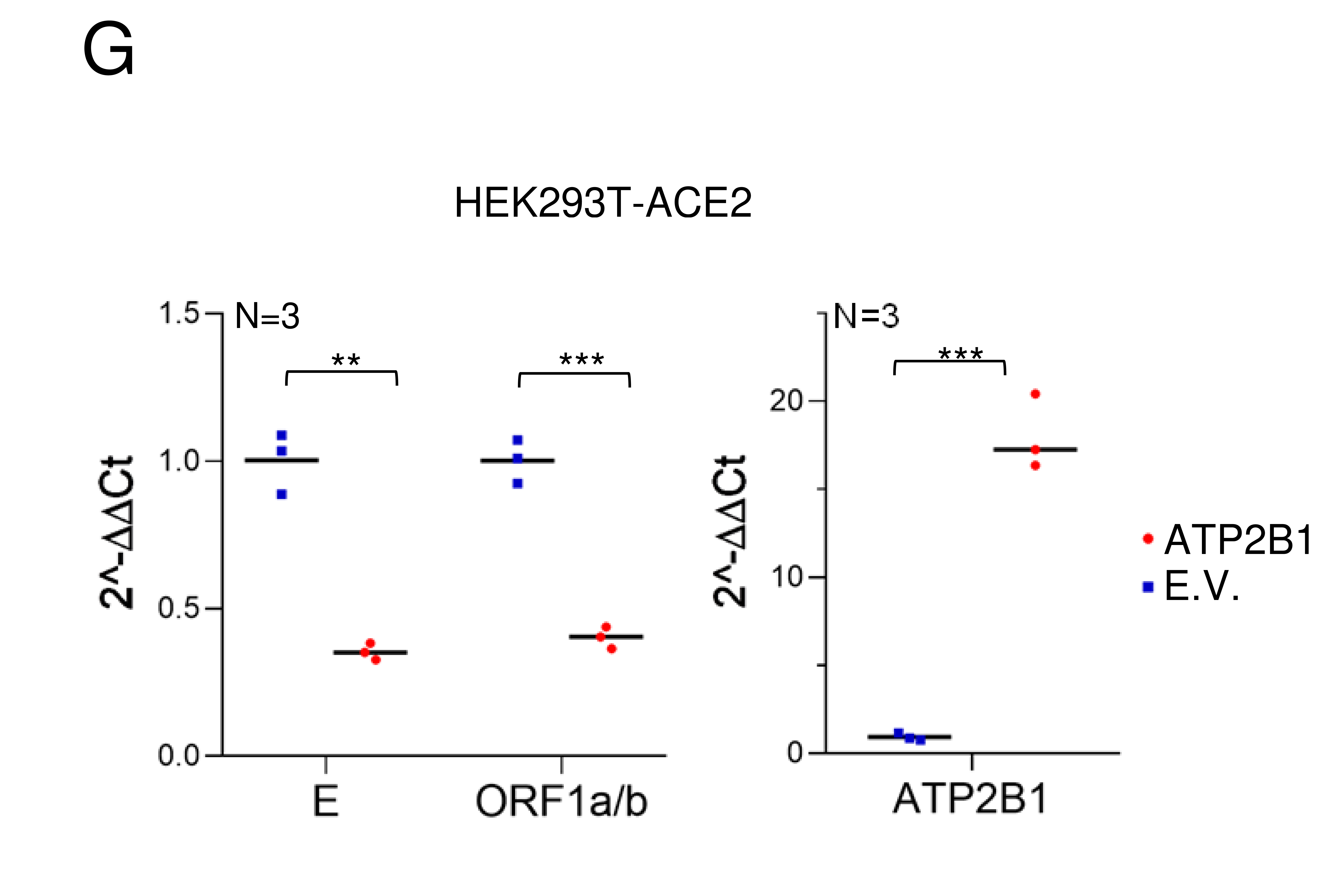

Supplement: Supplementary file 4 — Source data Fig. 2 [file 44319_2024_164_MOESM4_ESM.zip › Figure2/Figure2G/Figure2G.tif]

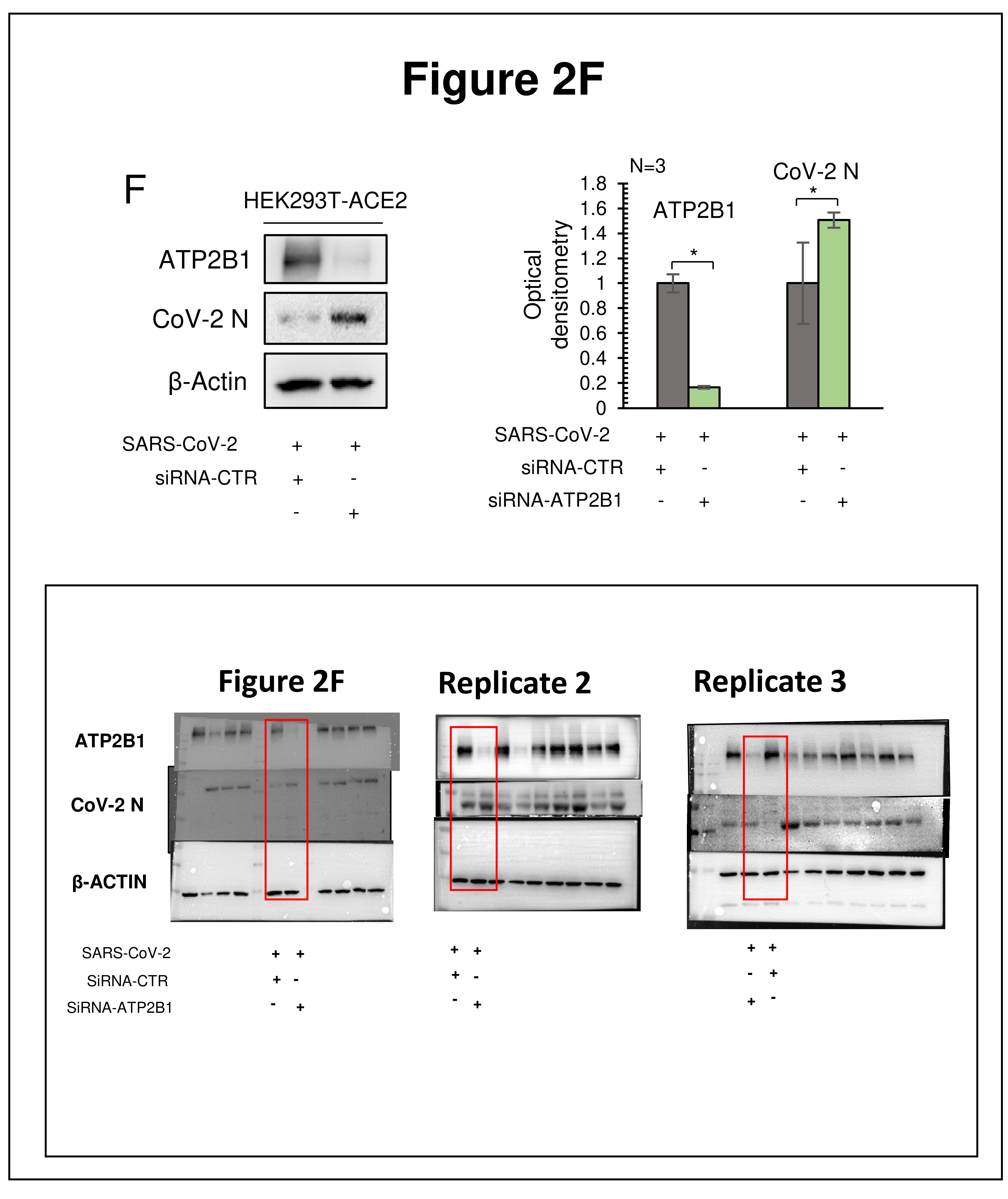

Supplement: Supplementary file 4 — Source data Fig. 2 [file 44319_2024_164_MOESM4_ESM.zip › Figure2/Figure2F-EX2E/Figure2F.tif]

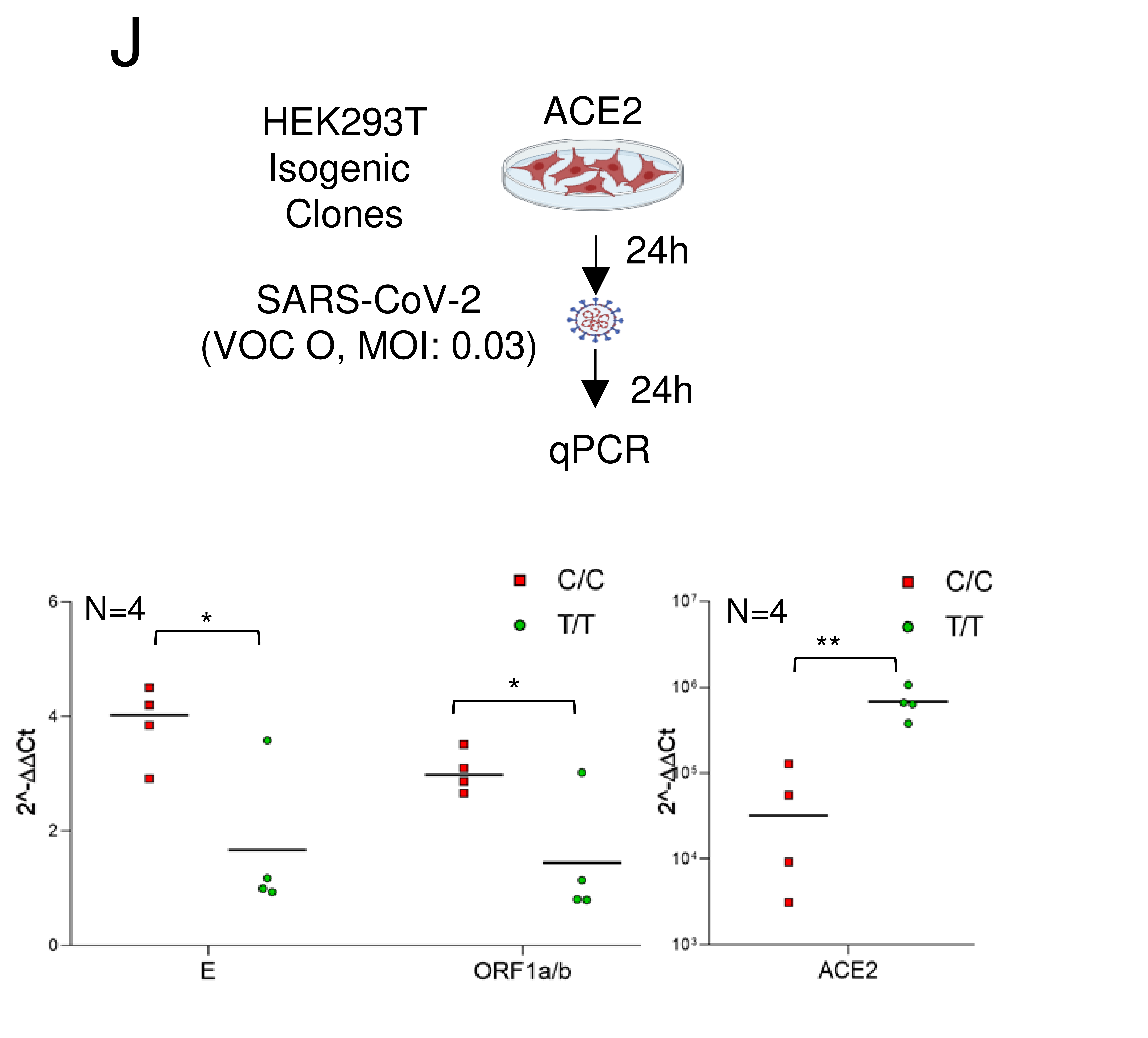

Supplement: Supplementary file 5 — Source data Fig. 3 [file 44319_2024_164_MOESM5_ESM.zip › Figure3/Figure3J/Figure3J.tif]

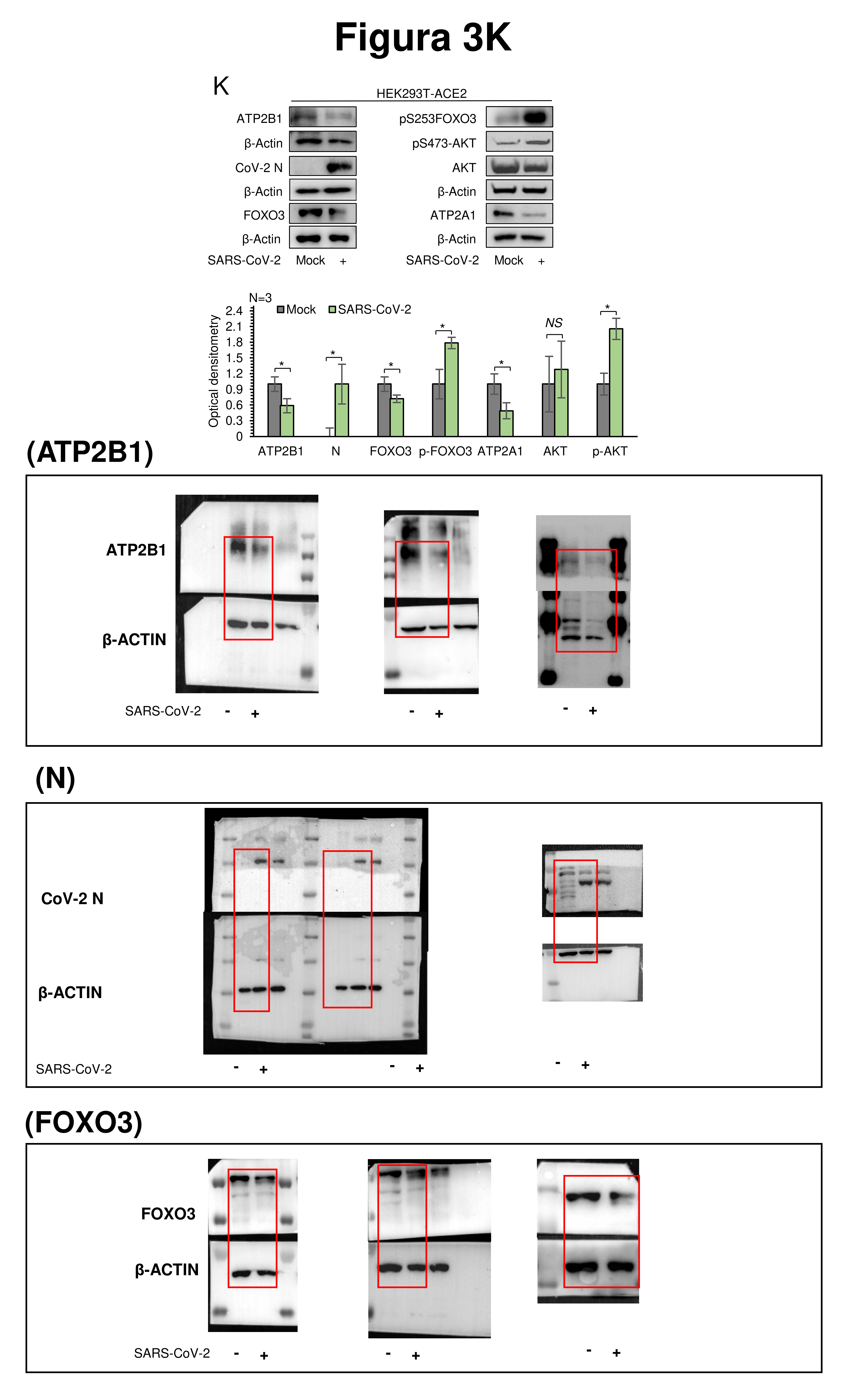

Supplement: Supplementary file 5 — Source data Fig. 3 [file 44319_2024_164_MOESM5_ESM.zip › Figure3/Figure3K/figure3K-a.tif]

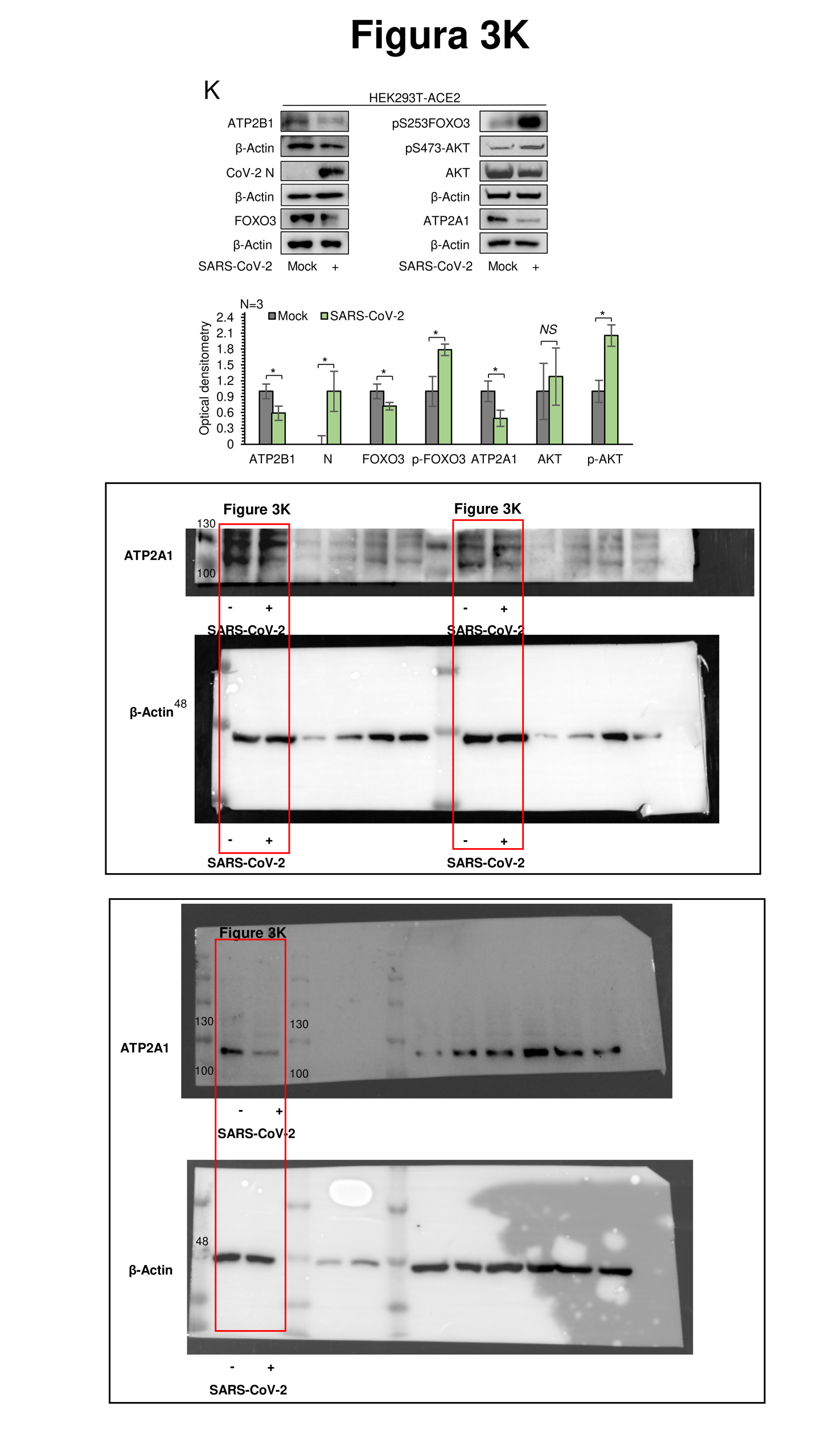

Supplement: Supplementary file 5 — Source data Fig. 3 [file 44319_2024_164_MOESM5_ESM.zip › Figure3/Figure3K/figure3K-c.tif]

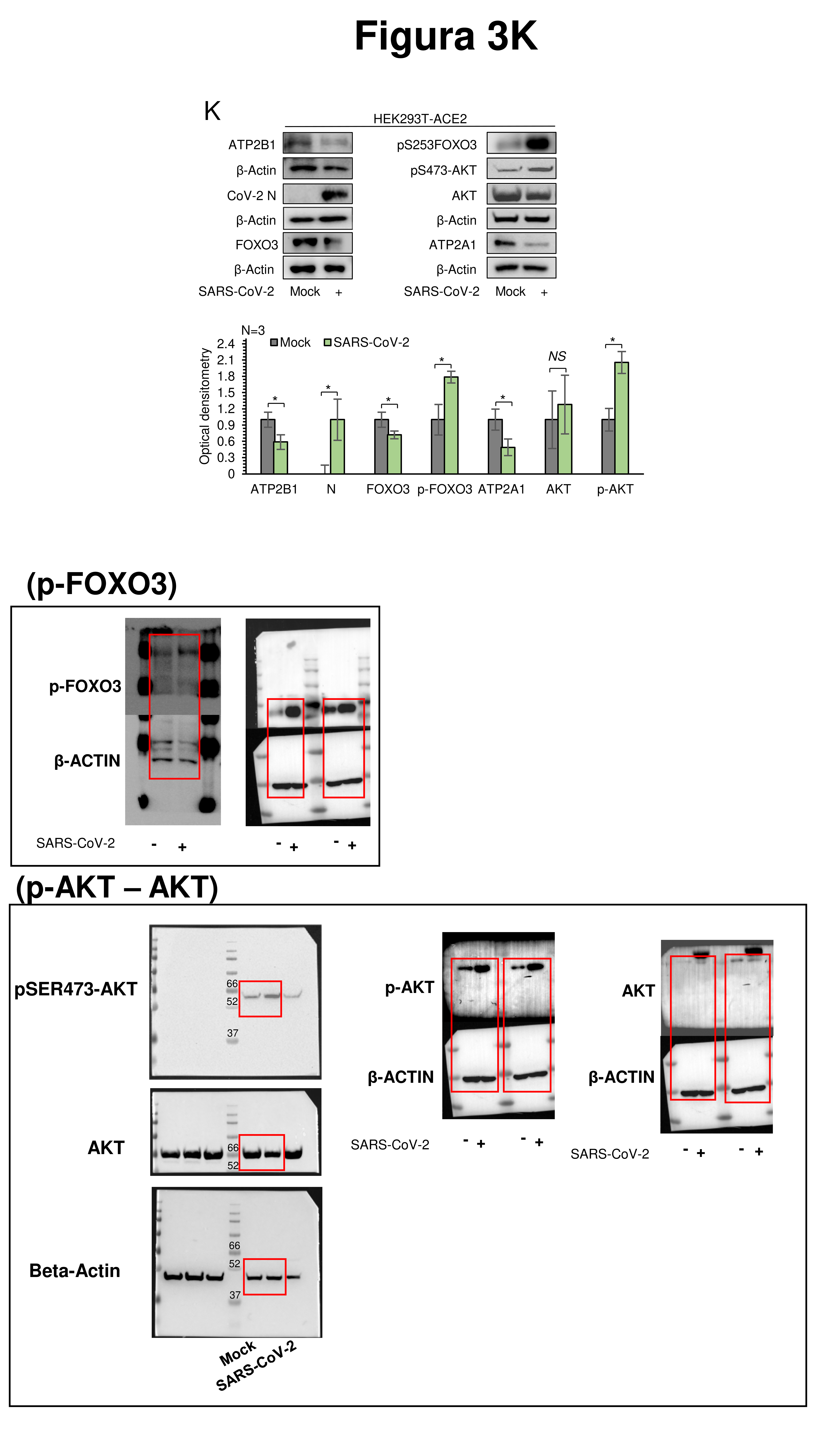

Supplement: Supplementary file 5 — Source data Fig. 3 [file 44319_2024_164_MOESM5_ESM.zip › Figure3/Figure3K/figure3K-b.tif]

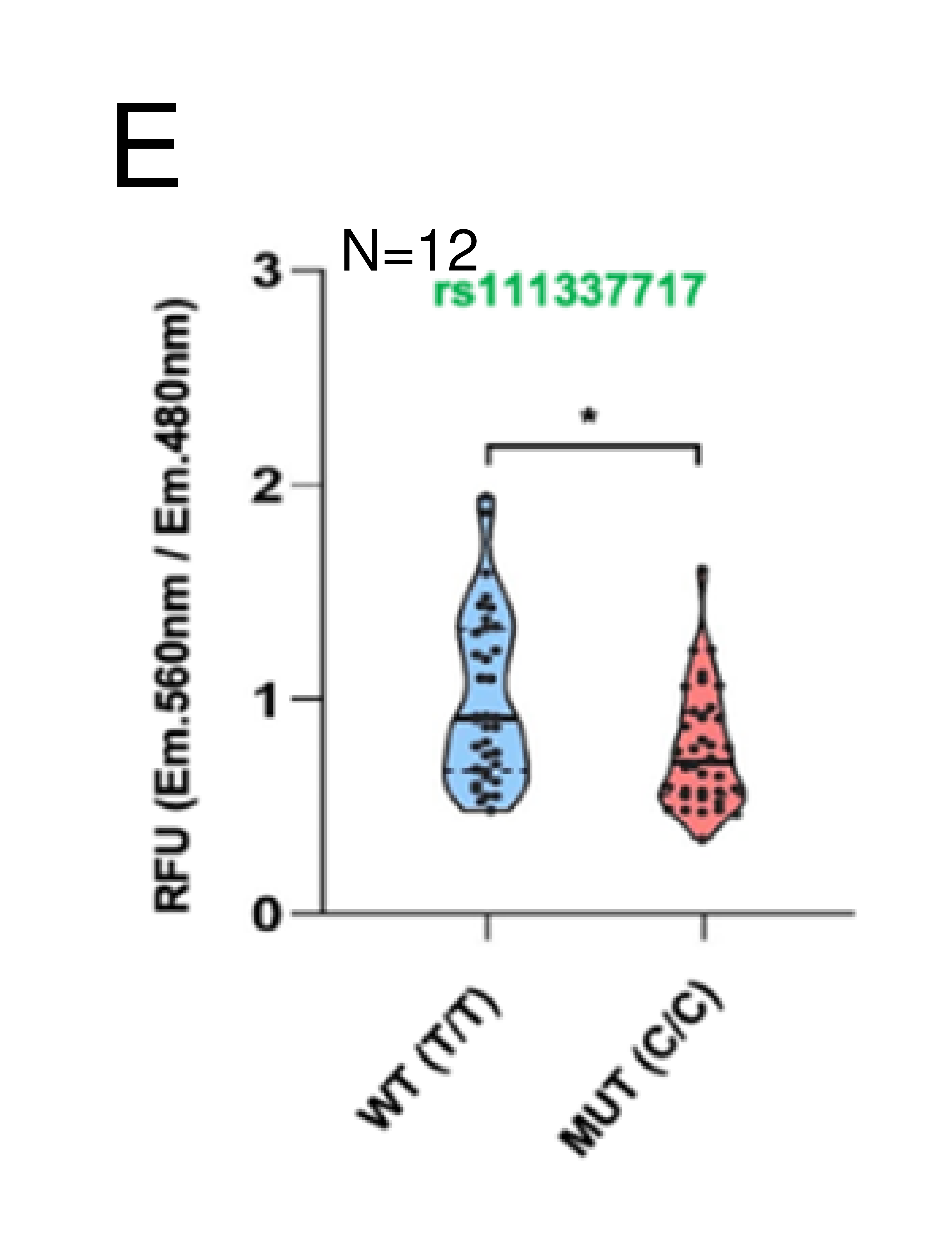

Supplement: Supplementary file 5 — Source data Fig. 3 [file 44319_2024_164_MOESM5_ESM.zip › Figure3/Figure3E/Figure3E.tif]

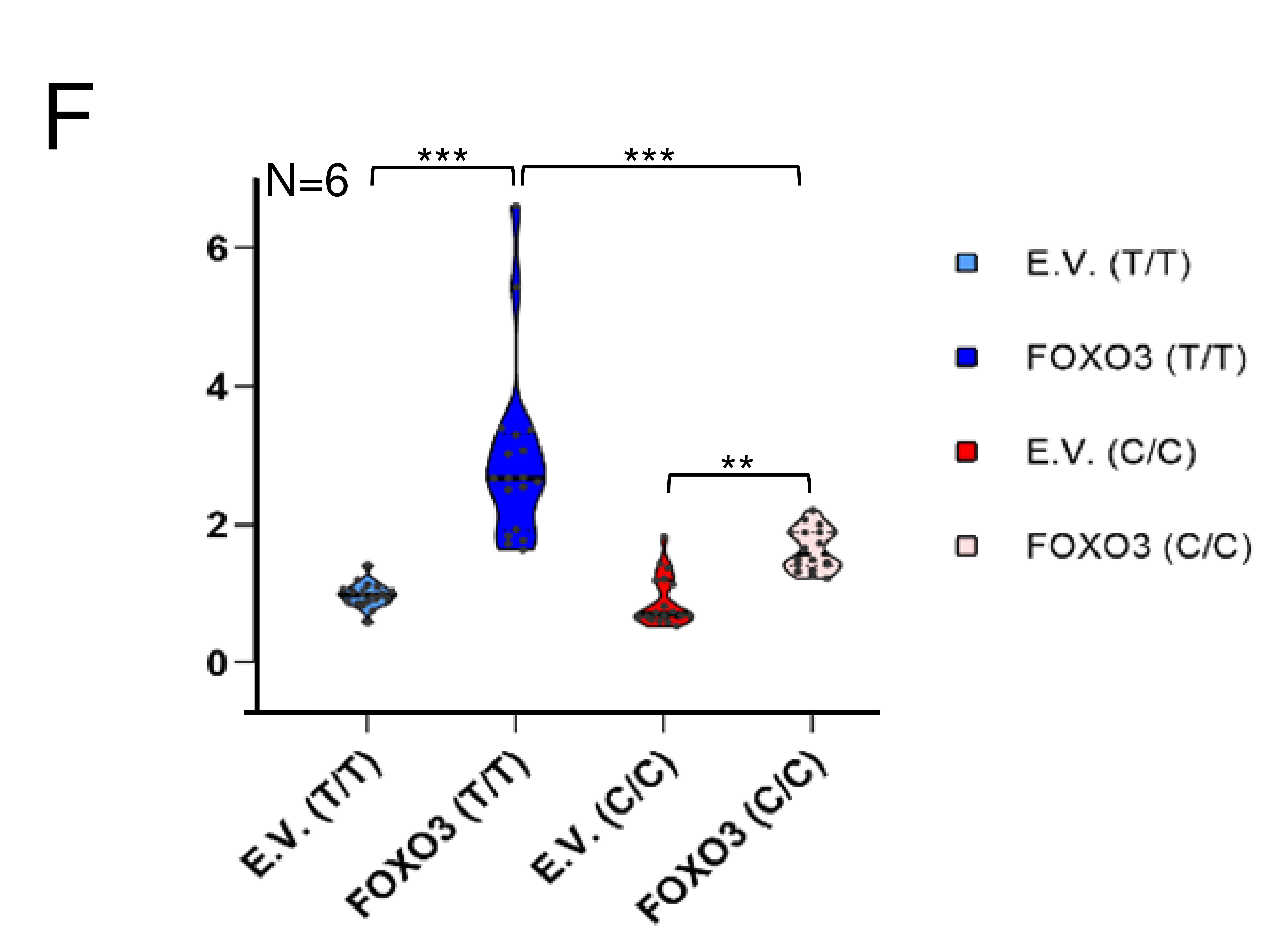

Supplement: Supplementary file 5 — Source data Fig. 3 [file 44319_2024_164_MOESM5_ESM.zip › Figure3/Figure3F/Figure3F.tif]

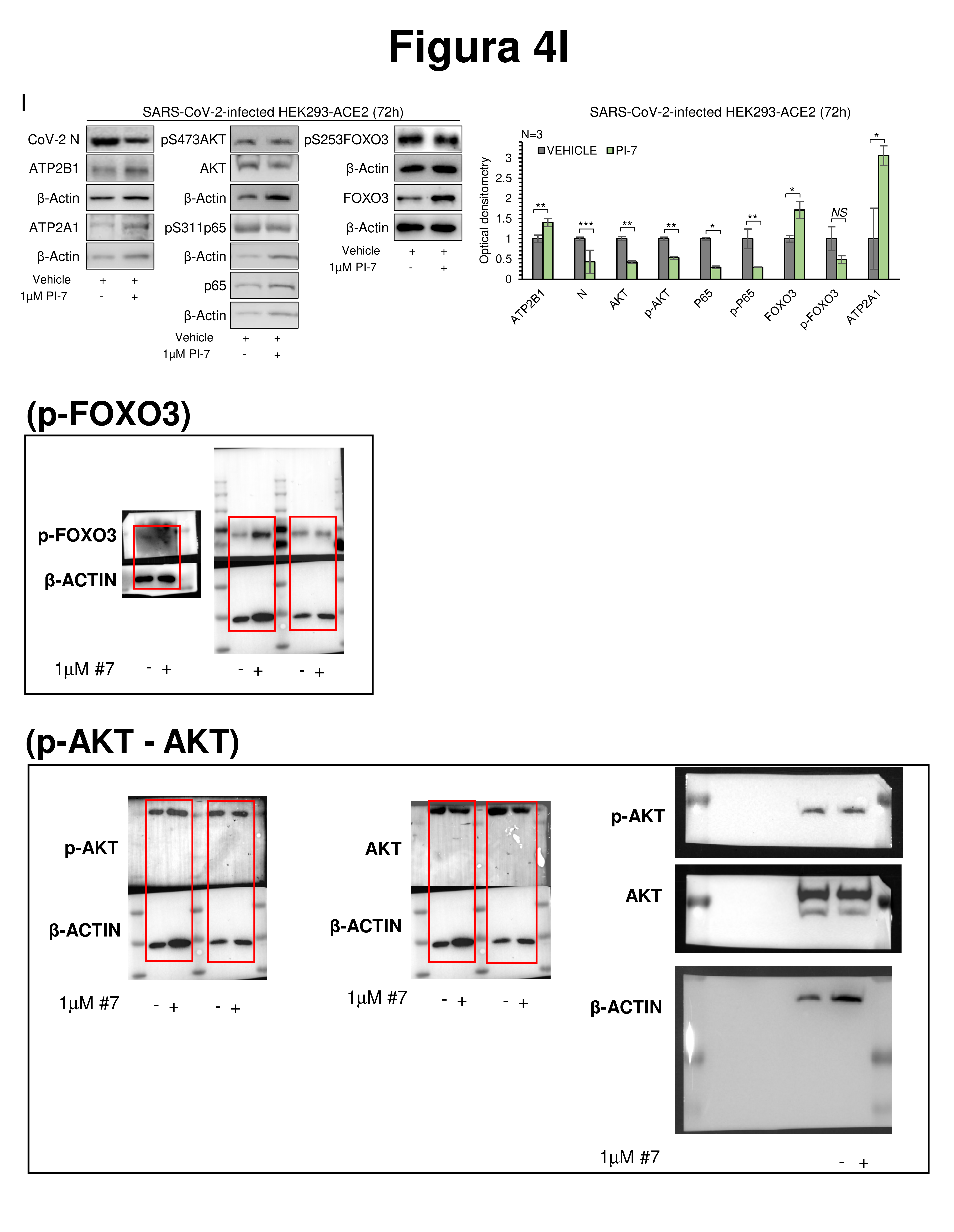

Supplement: Supplementary file 6 — Source data Fig. 4 [file 44319_2024_164_MOESM6_ESM.zip › Figure4/Figure4I-EX4H/Figure4I-b.tif]

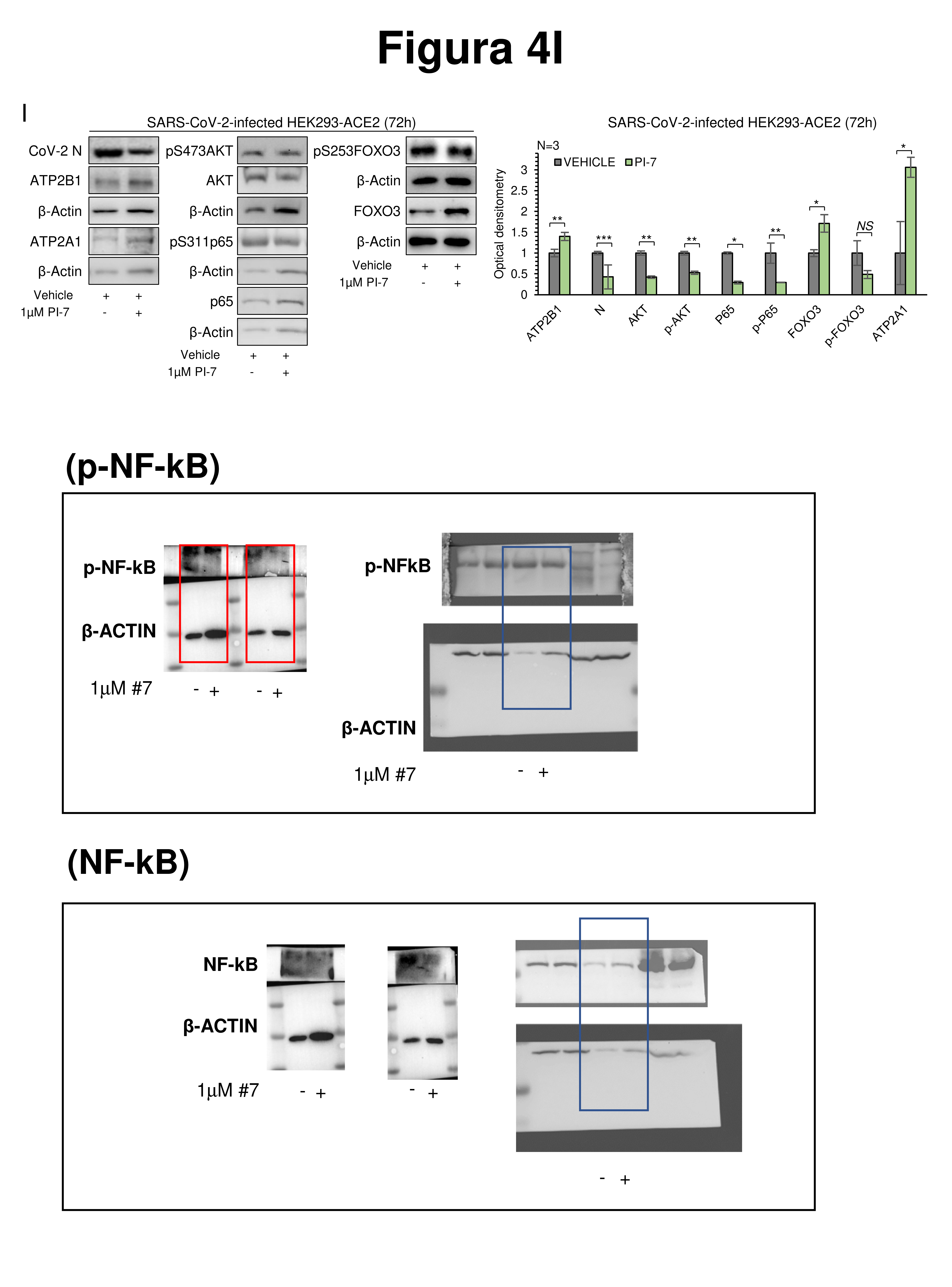

Supplement: Supplementary file 6 — Source data Fig. 4 [file 44319_2024_164_MOESM6_ESM.zip › Figure4/Figure4I-EX4H/Figure4I-c.tif]

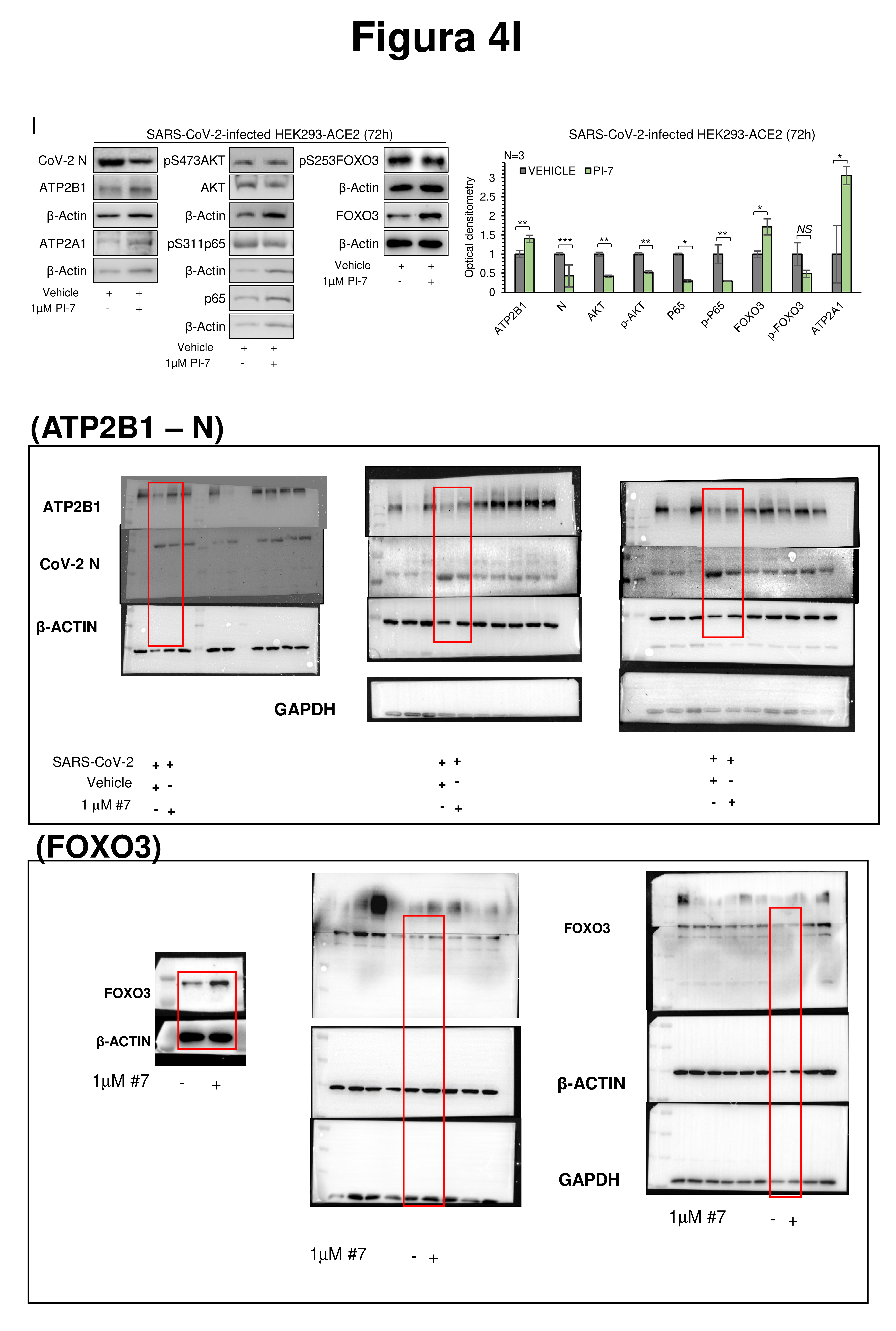

Supplement: Supplementary file 6 — Source data Fig. 4 [file 44319_2024_164_MOESM6_ESM.zip › Figure4/Figure4I-EX4H/Figure4I-a.tif]

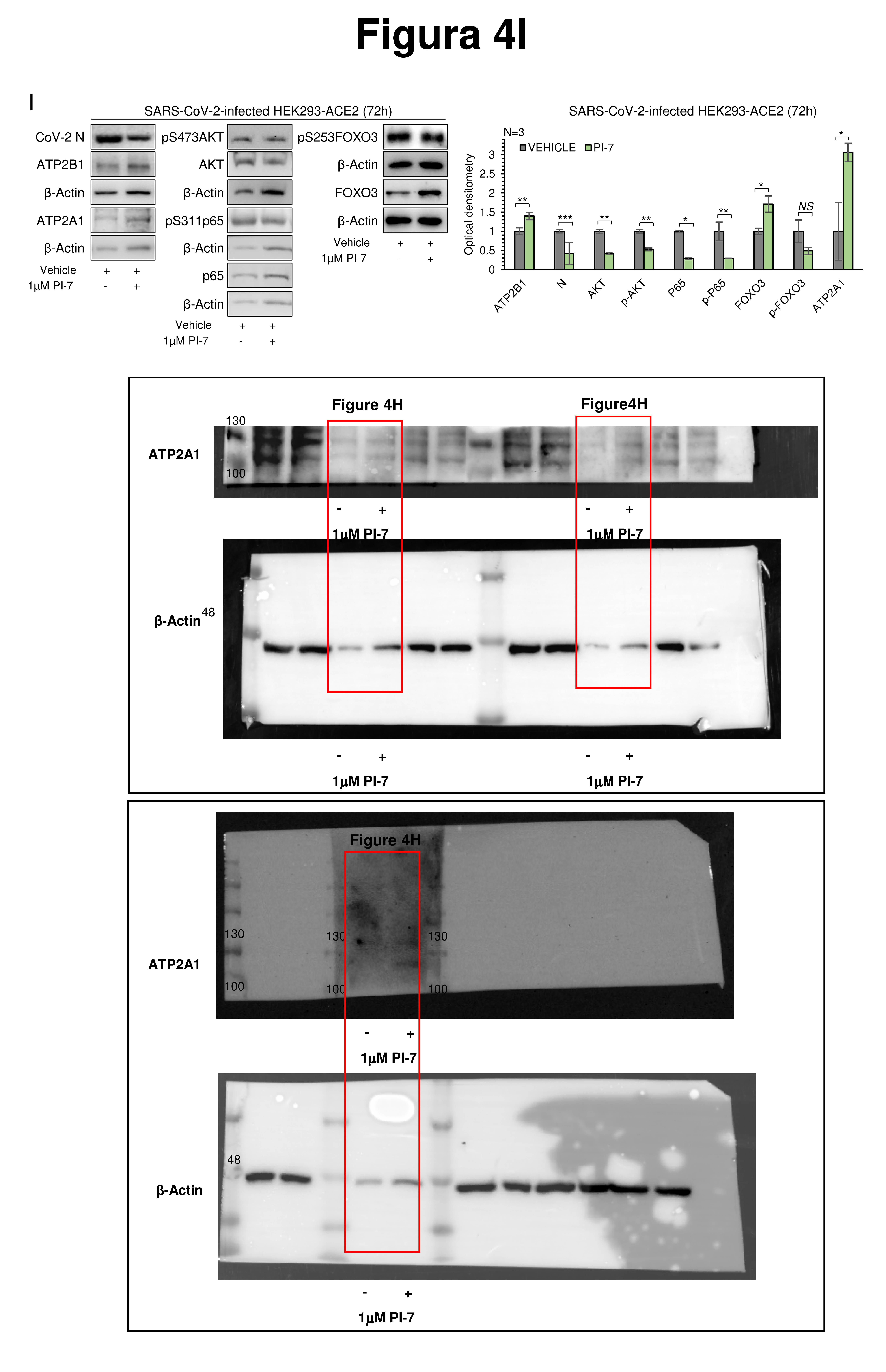

Supplement: Supplementary file 6 — Source data Fig. 4 [file 44319_2024_164_MOESM6_ESM.zip › Figure4/Figure4I-EX4H/Figure4I-d.tif]

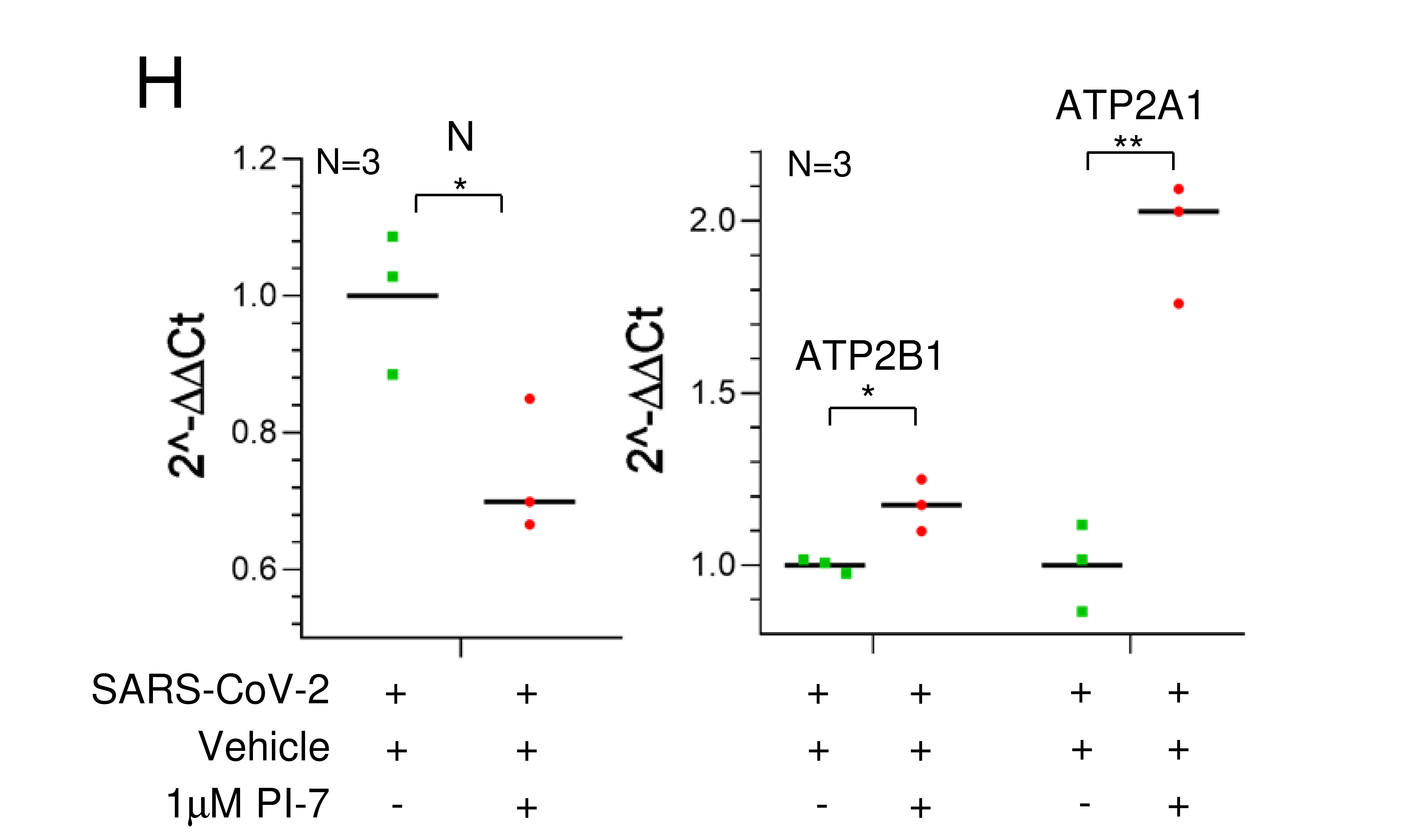

Supplement: Supplementary file 6 — Source data Fig. 4 [file 44319_2024_164_MOESM6_ESM.zip › Figure4/Figure4H-EX4I/Figure4H.tif]

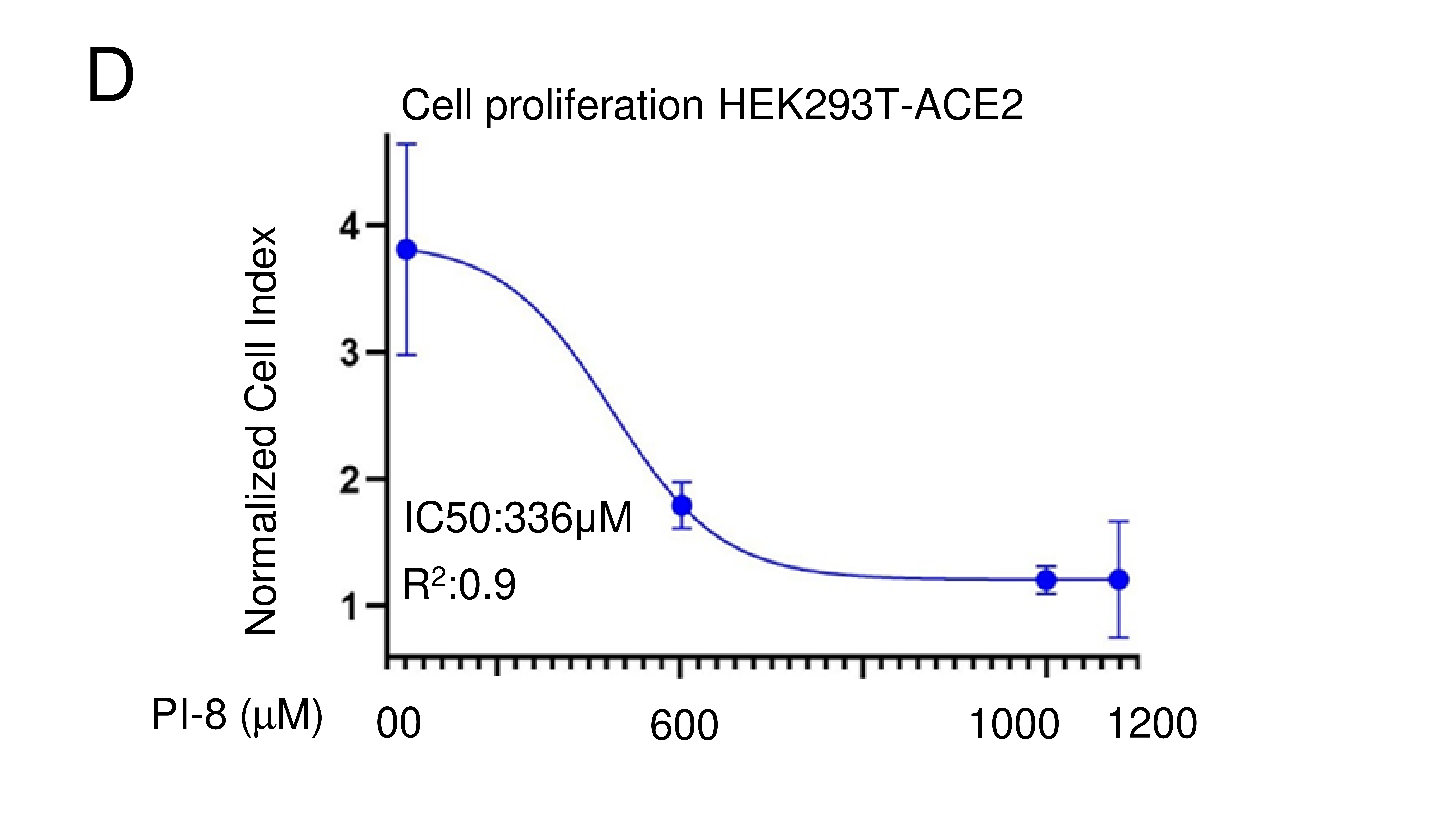

Supplement: Supplementary file 6 — Source data Fig. 4 [file 44319_2024_164_MOESM6_ESM.zip › Figure4/Figure4D/Figure4D.tif]

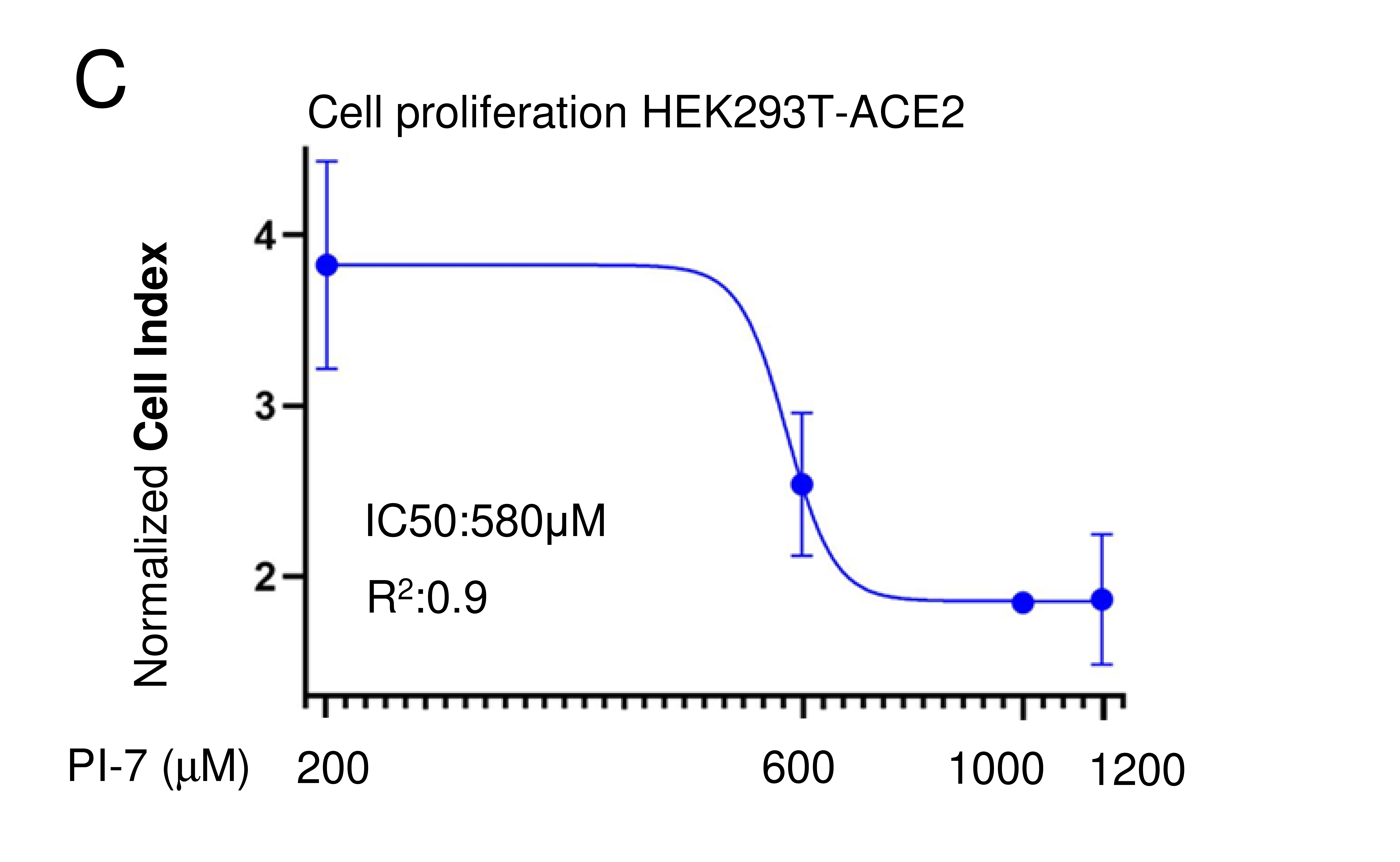

Supplement: Supplementary file 6 — Source data Fig. 4 [file 44319_2024_164_MOESM6_ESM.zip › Figure4/Figure4C/Figure4C.tif]

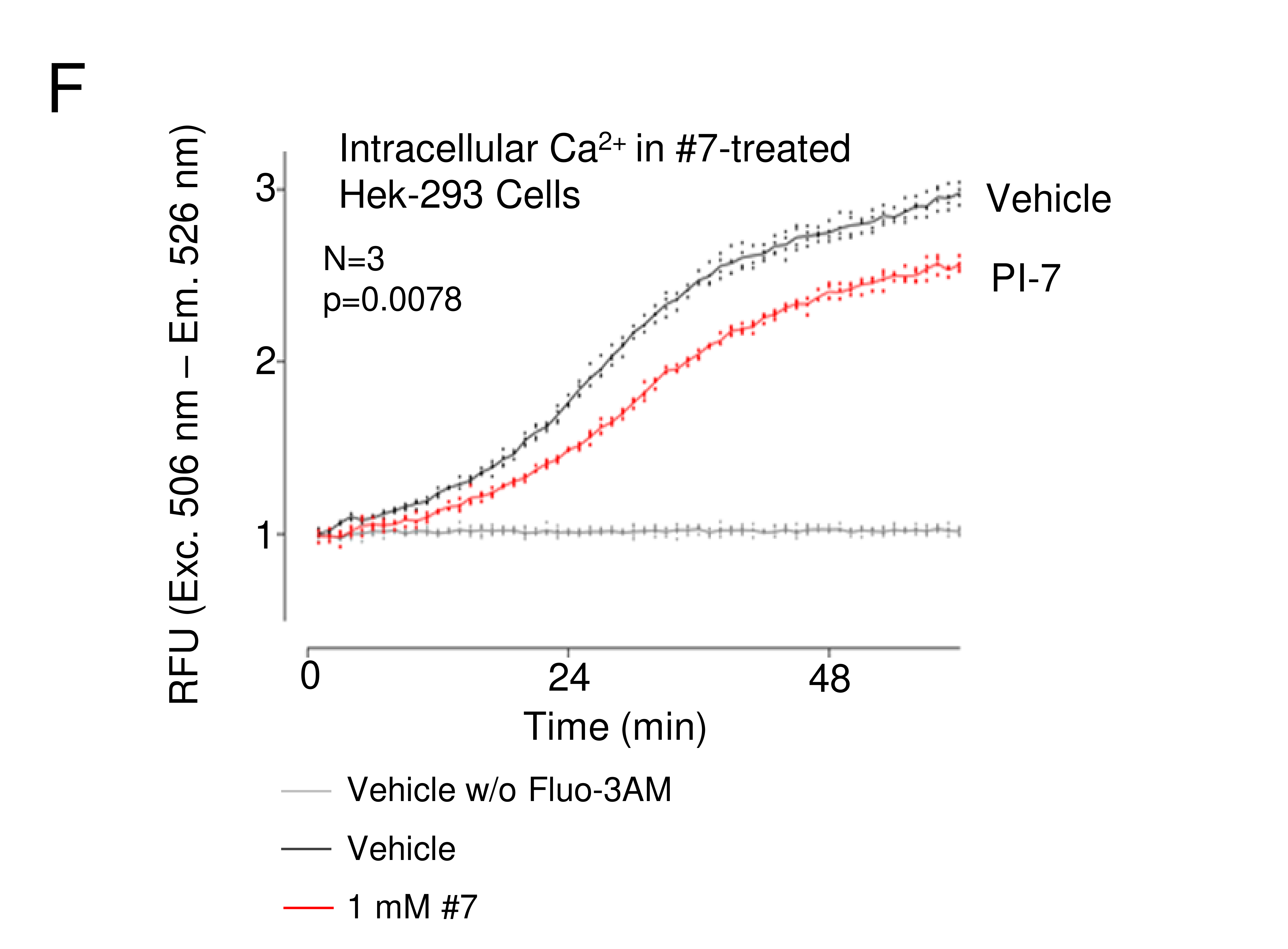

Supplement: Supplementary file 6 — Source data Fig. 4 [file 44319_2024_164_MOESM6_ESM.zip › Figure4/Figure4F/figure4F.tif]
