# Supplementary material for: Targeting ATP2B1 impairs PI3K/Akt/FOXO signaling and reduces SARS-COV-2 infection and replication
Source: EMBO Rep. 2024 May 30;25(7):12. doi: 10.1038/s44319-024-00164-z (PMC11239940; doi:10.1038/s44319-024-00164-z)
Supplement: Supplementary file 9 — Expanded View Figures [file 44319_2024_164_MOESM9_ESM.pdf]

Expanded View Figures

Figure EV1. Deregulated Ca^{2+} pumps during SARS-CoV-2 infection, including ATP2B1. Related to Fig. 1.

(**A**) A representative immunoblotting analysis using antibodies against the CoV-2 N protein from cells treated with escalating concentration of "gossypol-pubChem CID 3503" at 1 and 5 µM and vehicle as control in SARS-CoV-2 (VOC Δ) infected HEK293T-ACE2 cells. β-Actin was used as the loading control. (**B**) Quantification of Cov-2 N gene ($2^{-\Delta\Delta Ct}$) in gossypol-treated HEK293T-ACE2 (5 µM—48 h) and infected as in (**A**). Cells treated with vehicle were used as negative control. Scattered plot shows the individual value and mean as indicated by the horizontal black lines of $N = 3$ biological replicates. Unpaired two-tailed *T* Student tests Bonferroni corrected. *$p < 0.05$ (vehicle vs. gossypol); the other comparisons are not statistically significant, as expected. (**C**) A representative immunoblotting analysis using antibodies against the Cov-2 N protein on total protein lysates obtained from human primary epithelial nasal cells treated with BAPTA at 20 µM concentration. Vehicle-treated cells were used as control. β-Actin is used as the loading control. (**D**) RNA Sequencing (RNA-seq) analyses was performed in HEK293T-ACE2 cells treated as in Fig. 2E. (**E**) The Gene Set Enrichment Analysis (see GSEA project on https://doi.org/10.1073/pnas.0506580102) is applied for the identification of deregulated key genes and pathways. KEGG pathways analyses by statistical KS global test, $P < 0.05$. KEGG pathway enrichment analysis indicates those significant deregulated genes were highly clustered in calcium signaling pathway (red box). *P* adj: adjusted *P* values. (**F**) In silico analysis of publicly available datasets of single-cell RNA sequencing (https://singlecell.broadinstitute.org) for the expression of the plasma membrane calcium ATPases members (PMCAs or ATP2B1-4) of the large family of type Ca^{2+}ion pumps in multiple cell type in the lung parenchyma (including alveolar macrophages and in the alveolar epithelial cells type I and type II). (**G**) Literature public search on available datasets obtained from a single-nuclei RNA-seq (snRNA-seq) on >116,000 nuclei from n.19 COVID-19 autopsy lungs and n.7 pre-pandemic controls (Melms et al, 2021); to verify expression of PMCAs and SERCAs pumps (ATP2B1-4 and ATP2A1-3 genes, respectively). The numbers within the dots are the median percentage level of expression between the two populations tested. (**H**) Expression levels of ATP2B1 in single-nuclei RNA-seq database (snRNA-seq) as described above in (**G**). Source data are available online for this figure.

© The Author(s)

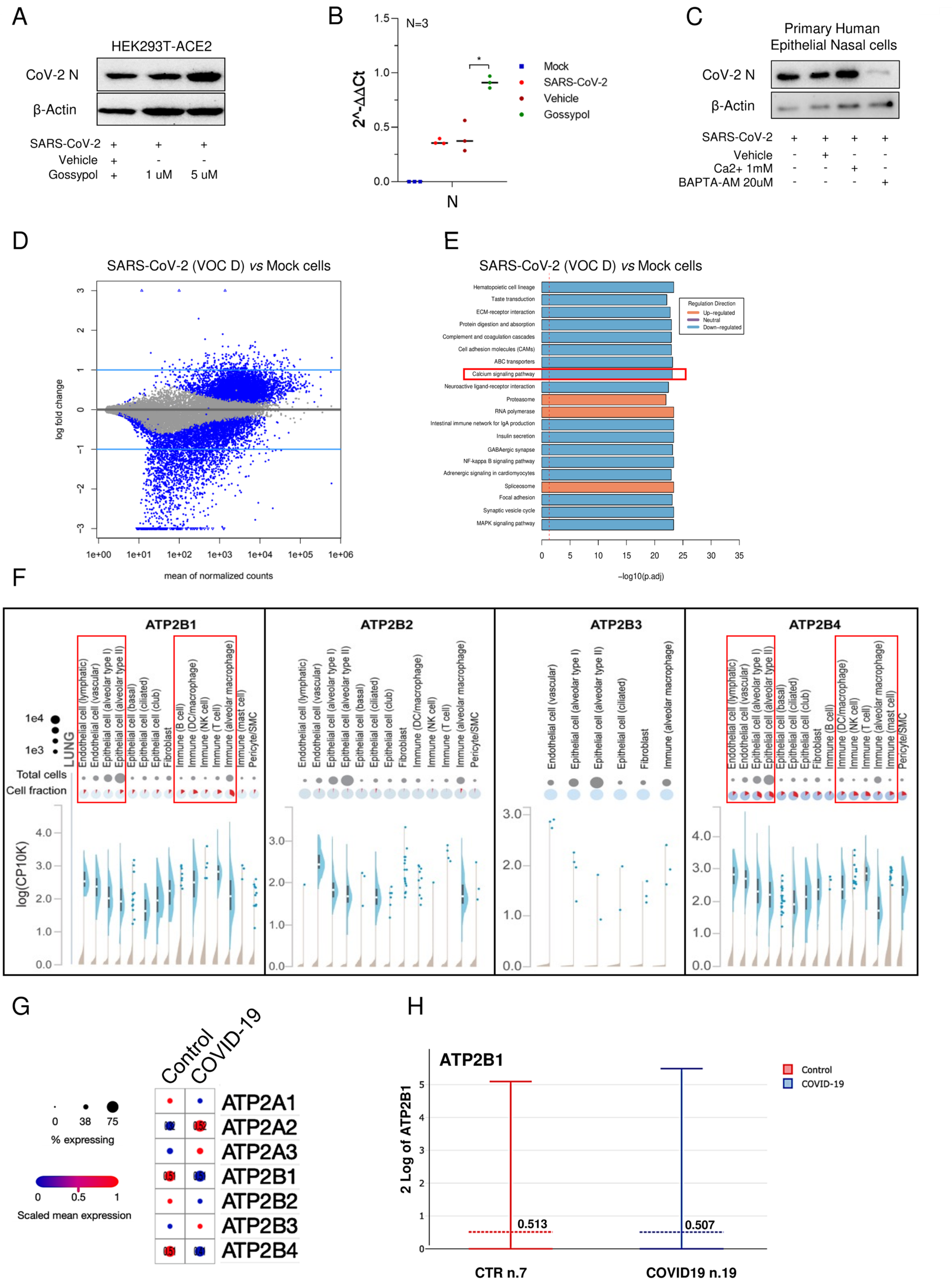
A
HEK293T-ACE2
CoV-2 N
β-Actin
SARS-CoV-2 + + +
Vehicle + - -
Gossypol + 1 uM 5 uM
B
N=3
2^-ΔΔCt
Mock
SARS-CoV-2
Vehicle
Gossypol
N
C
Primary Human Epithelial Nasal cells
CoV-2 N
β-Actin
SARS-CoV-2 + + + +
Vehicle - + - -
Ca2+ 1mM - - + -
BAPTA-AM 20uM - - - +
D
SARS-CoV-2 (VOC D) vs Mock cells
log fold change
mean of normalized counts
E
SARS-CoV-2 (VOC D) vs Mock cells
Hematopoietic cell lineage
Taste transduction
ECM-receptor interaction
Protein digestion and absorption
Complement and coagulation cascades
Cell adhesion molecules (CAMs)
ABC transporters
Calcium signaling pathway
Neuroactive ligand-receptor interaction
Proteasome
RNA polymerase
Intestinal immune network for IgA production
Insulin secretion
GABAergic synapse
NF-kappa B signaling pathway
Adrenergic signaling in cardiomyocytes
Spliceosome
Focal adhesion
Synaptic vesicle cycle
MAPK signaling pathway
Regulation Direction
Up-regulated
Neutral
Down-regulated
−log10(p.adj)
F
ATP2B1
ATP2B2
ATP2B3
ATP2B4
LUNG
Total cells
Cell fraction
log(CP10K)
G
Control
COVID-19
ATP2A1
ATP2A2
ATP2A3
ATP2B1
ATP2B2
ATP2B3
ATP2B4
% expressing
Scaled mean expression
H
ATP2B1
2 Log of ATP2B1
0.513
0.507
Control
COVID-19
CTR n.7
COVID19 n.19

 © The Author(s)

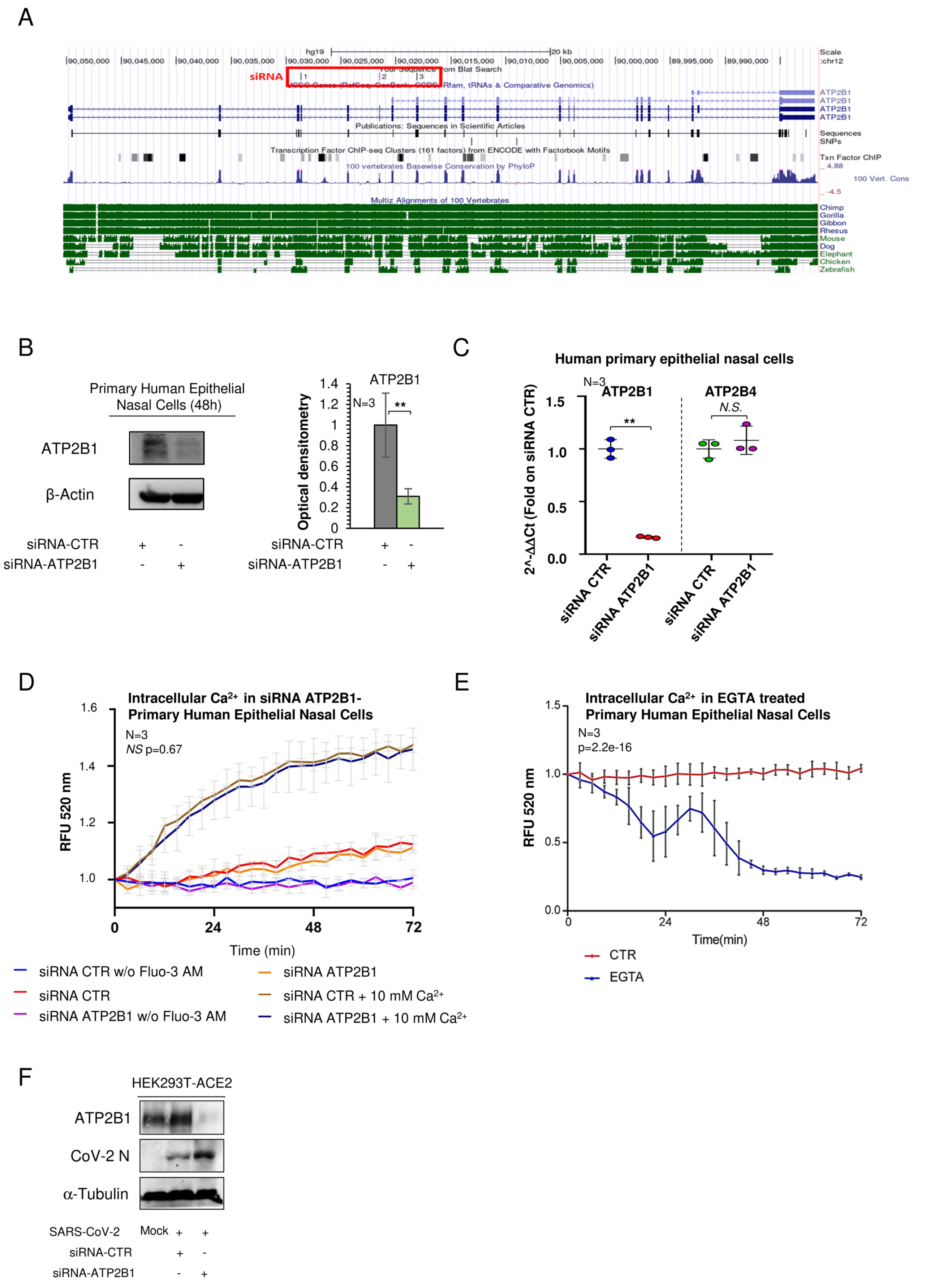
A
siRNA
B
Primary Human Epithelial Nasal Cells (48h)
ATP2B1
β-Actin
siRNA-CTR + -
siRNA-ATP2B1 - +
ATP2B1
N=3
**
Optical densitometry
C
Human primary epithelial nasal cells
N=3
ATP2B1
ATP2B4
N.S.
2^-ΔΔCt (Fold on siRNA CTR)
siRNA CTR
siRNA ATP2B1
D
Intracellular Ca2+ in siRNA ATP2B1-Primary Human Epithelial Nasal Cells
N=3
NS p=0.67
RFU 520 nm
Time (min)
siRNA CTR w/o Fluo-3 AM
siRNA CTR
siRNA ATP2B1 w/o Fluo-3 AM
siRNA ATP2B1
siRNA CTR + 10 mM Ca2+
siRNA ATP2B1 + 10 mM Ca2+
E
Intracellular Ca2+ in EGTA treated Primary Human Epithelial Nasal Cells
N=3
p=2.2e-16
RFU 520 nm
Time(min)
CTR
EGTA
F
HEK293T-ACE2
ATP2B1
CoV-2 N
α-Tubulin
SARS-CoV-2 Mock + +
siRNA-CTR + -
siRNA-ATP2B1 - +

Figure EV2. Reduced ATP2B1 protein levels promote SARS-CoV-2 replication. Related to Fig. 2.

(**A**) Representation of human ATP2B1 region recognized by siRNA as reported in the UCSC Genome Browser on Human Dec. 2013 (GRCh38/hg38) Assembly (https://genome.ucsc.edu/). At the bottom, the alignment of this genomic region among different species is shown. (**B**) Left: representative immunoblotting analysis using antibodies against the ATP2B1 protein on human primary epithelial nasal cells transiently treated with siRNA against ATP2B1 (siRNA-ATP2B1) for 48 h. Cells treated with a pool of three unrelated siRNAs (siRNA- CTR) were used as negative controls. β-Actin is used as the loading control. Right: Densitometric analysis of the ATP2B1 band intensities in blots. Data are means ± SD of $N = 3$ biological replicates. Unpaired two-tailed *T* Student tests, $^{**}P < 0.01$. (**C**) Quantification of mRNA abundance for ATP2B1 and ATP2B4 ($2^{-\Delta\Delta Ct}$) in cells as treated as in (**B**). Data are means ± SD of $N = 3$ biological replicates. Unpaired two-tailed *T* Student *t* test, $^{**}P < 0.01$; NS not significant. (**D**) Quantification of relative fluorescence changes of Fluo3-AM as a measure of intracellular Ca^{2+} levels in cells treated as in (**B**) for up to 72 min. Results are expressed as means ± SEM of $N = 3$ biological replicates. One-way ANOVA and KS test, $P = 0.6748$; NS = not significant between siRNA control (brown) and siRNA-ATP2B1 (dark blue) in presence of 10 mM Ca^{2+}. (**E**) Quantification of relative fluorescence changes of Fluo3-AM as a measure of intracellular Ca^{2+} levels for up to 72 min in human primary epithelial nasal cells treated with EGTA 1 mM. Results are expressed as means ± SEM of $N = 3$ biological replicates. One-way ANOVA and KS tests, $P = 2.2e-16$. (**F**) A representative immunoblotting analysis using antibodies against the ATP2B1 protein for total protein lysates obtained from cells treated as described in Fig. 2E. α-Tubulin is used as loading control. Mock-infected cells are used as control. Source data are available online for this figure

 © The Author(s)

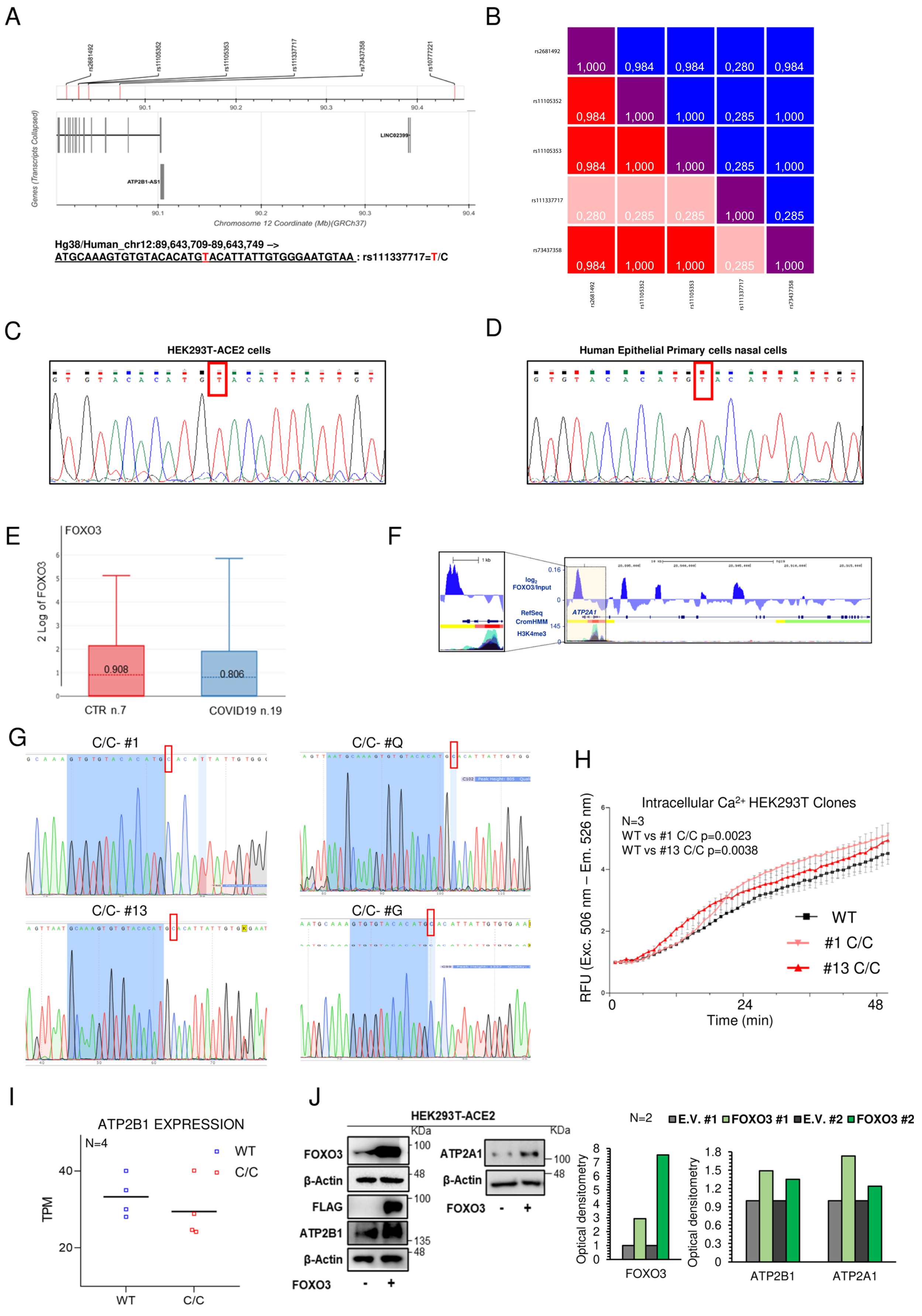
A
Chromosome 12 Coordinate (Mb)(GRCh37)
Genes (Transcripts Collapsed)
ATP2B1-AS1
LINC02399
Hg38/Human_chr12:89,643,709-89,643,749 –>
ATGCAAAGTGTGTACACATGTACATTATTGTGGGAATGTAA : rs111337717=T/C
B
rs2681492
rs11105352
rs11105353
rs111337717
rs73437358
C
HEK293T-ACE2 cells
D
Human Epithelial Primary cells nasal cells
E
FOXO3
2 Log of FOXO3
0.908
0.806
CTR n.7
COVID19 n.19
F
log2 FOXO3/Input
RefSeq
CromHMM
H3K4me3
ATP2A1
G
C/C- #1
C/C- #Q
C/C- #13
C/C- #G
H
Intracellular Ca2+ HEK293T Clones
N=3
WT vs #1 C/C p=0.0023
WT vs #13 C/C p=0.0038
WT
#1 C/C
#13 C/C
RFU (Exc. 506 nm – Em. 526 nm)
Time (min)
I
ATP2B1 EXPRESSION
N=4
WT
C/C
TPM
J
HEK293T-ACE2
KDa
FOXO3
β-Actin
FLAG
ATP2B1
ATP2A1
N=2
E.V. #1
FOXO3 #1
E.V. #2
FOXO3 #2
Optical densitometry

Figure EV3. The homozygous intronic *ATP2B1* variant rs11337717 is responsible for increased SARS-CoV-2 replication in COVID-19 patients by transcriptional regulation of FOXO3. Related to Fig. 3.

(**A, B**) Linkage disequilibrium (LD) analyses on the top n.5 SNPs (rs11105352; rs11105353; rs73437358; rs111337717; rs2681492) in order to select those which are independent. The SNP rs10777221 is excluded from these analyses because located at most 5′ region in extragenic ATP2B1 locus region (**A**). The graph in (**B**) shows the only SNP not in LD is rs111337717 (black boxes). (**C**) Sanger DNA sequencing of the genomic region of ATP2B1 locus (chr12:89,643,709-89,643,749) in HEK293T-ACE2 cells to exclude the presence of intronic variance potentially responsible for altered transcriptional levels of ATP2B1 gene. The red box indicates the nucleotide wild type allele "T" for the SNP here studied. (**D**) Sanger DNA sequencing of the genomic region of ATP2B1 locus (chr12:89,643,709-89,643,749) in human primary epithelial nasal cells to exclude the presence of intronic variants potentially responsible for altered transcriptional levels of ATP2B1 gene. (**E**) FOXO3 Expression in UMAP by disease ontology labels single-nuclei RNA-seq (snRNA-seq) analyses performed on >116,000 nuclei from n.19 COVID-19 autopsy lungs and n.7 pre-pandemic controls. Data measurements values of Log^2 FOX3 mRNA expression: in CTR donors n.7: max=5.856, center= 0.806, min=0; in COVID-19 n.19 affected patients: max=5.128, center=0.908, min=0. (**F**) Genome browser screenshots showing accumulation of normalized FOXO3 signal, together with CromHMM state segmentation and H3K4me3 signal (ENCODE), along the ATP2A1 gene in human cells. ForCromHMM state segmentation colors indicate: Bright Red—Promoter; Orange and yellow—enhancer; Green— Transcriptional transition. The expanded view of the highlighted region, on the left, shows FOXO3 peaks over ATP2A1 enhancer regions, as marked by yellow region of CromHMM. (**G**) Sanger DNA sequencing of the genomic region of ATP2B1 locus (chr12:89,643,709-89,643,749) in HEK293T-ACE2 relative to the CRISPR/Cas9 edited clones to show the presence of intronic homozygous variant (C/C) responsible for altered transcriptional levels of ATP2B1 gene. The red box indicates the nucleotide edited for the SNP here studied. (**H**) Quantification of relative fluorescence changes of Fluo3-AM as a measure of intracellular Ca^{2+} levels for up to 48 min in HEK293T isogenic clones. Results are expressed as means ± SEM of $N = 3$ biological replicates. One-way ANOVA and KS test. In details: WT vs. CC $P = 0.0023$; WT vs. CC $P = 0.0038$. (**I**) Quantification of ATP2B1 mRNA abundance in HEK293T ($N = 4$ homozygous C/C) isogenic clones compared to ($N = 4$ WT T/T) unedited clones. mRNA levels measured by RNA-seq were plotted using transcript per million (TPM). Scattered plots show individual value and mean as indicated by the horizonal black lines of $N = 4$ biological replicates. Fold change value $+/-2$. Statistical Mobin Wald test NS not significant. (**J**) Left: representative immunoblotting analysis using antibodies against FOXO3, ATP2B1, ATP2A1 as indicated proteins on total protein lysates obtained from HEK293T-ACE2 cells transiently transfected with the human FOXO3-encoding plasmid (FLAG antibody positive) for 48 h. Empty vector transfected cells were used as negative control. β-Actin is used as the loading control. On the right: Densitometric analysis of FOXO3, ATP2B1 and ATP2A1 from $N = 2$ technical replicates. Source data are available online for this figure

 © The Author(s)

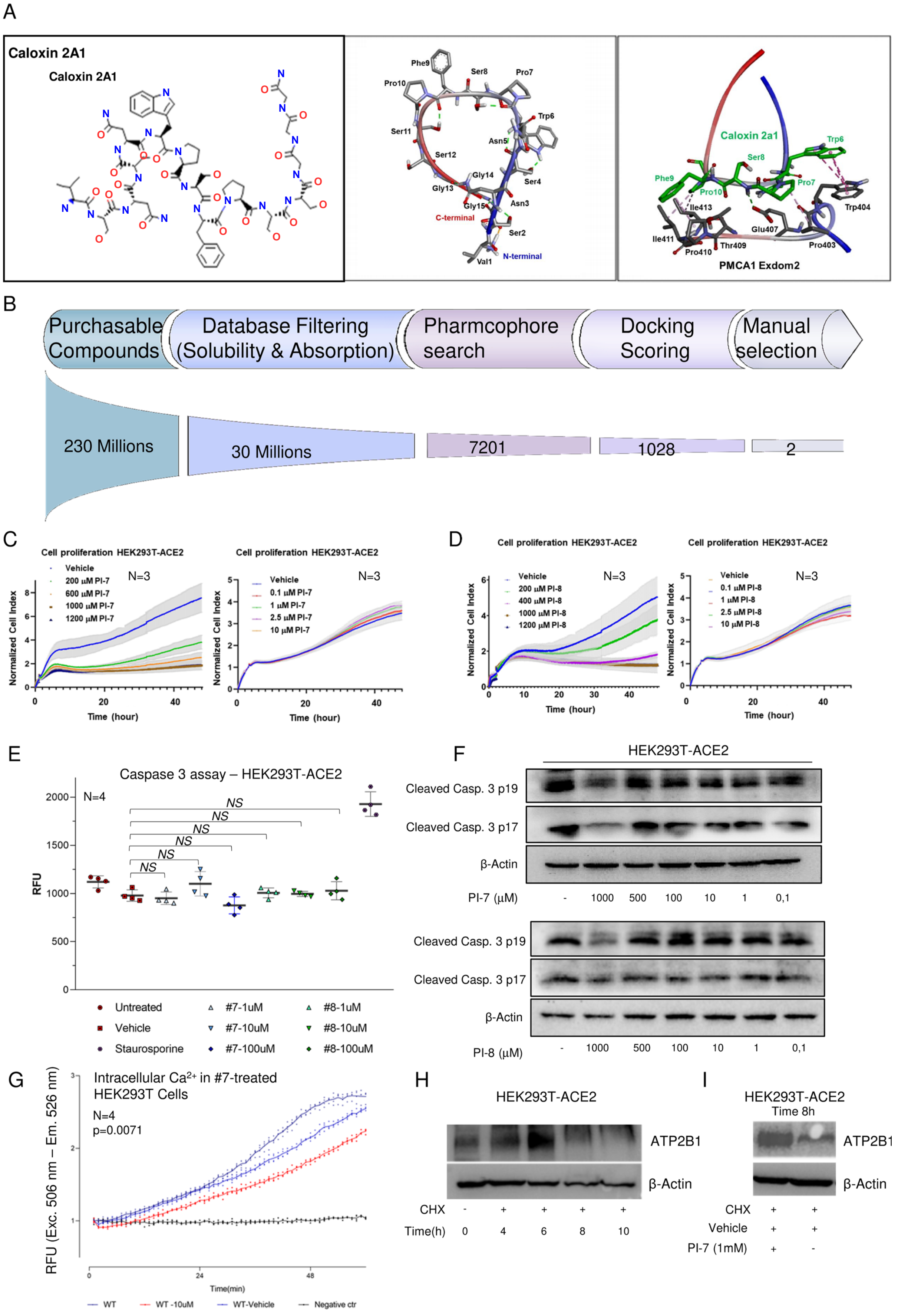
A
Caloxin 2A1
PMCA1 Exdom2
B
Purchasable Compounds
Database Filtering (Solubility & Absorption)
Pharmcophore search
Docking Scoring
Manual selection
230 Millions
30 Millions
7201
1028
2
C
Cell proliferation HEK293T-ACE2
D
Cell proliferation HEK293T-ACE2
E
Caspase 3 assay – HEK293T-ACE2
N=4
F
HEK293T-ACE2
Cleaved Casp. 3 p19
Cleaved Casp. 3 p17
β-Actin
PI-7 (μM)
PI-8 (μM)
G
Intracellular Ca2+ in #7-treated HEK293T Cells
N=4
p=0.0071
RFU (Exc. 506 nm – Em. 526 nm)
Time(min)
H
HEK293T-ACE2
ATP2B1
β-Actin
CHX
Time(h)
I
HEK293T-ACE2
Time 8h
ATP2B1
β-Actin
CHX
Vehicle
PI-7 (1mM)

Figure EV4. ATP2B1 impairment using a nontoxic "caloxin derivative" (compound PI-7) impairs intracellular Ca^{2+} levels. Related to Fig. 4.

(**A**) On the left: The sequence of caloxin 2a1 sequence, as peptide, is shown. On the right: The molecular modeling of ATP2B1-caloxin 2a1 structure by docking and energy minimization modeling via artificial intelligence as a drug design computational tool is shown. The pharmacophore model by using the structures ATP2B1–exodom-2 and caloxin 2a1 is also shown. Five pharmacophore features were produced. (**B**) Pipeline of the drug discovery is shown as described in the manuscript. (**C**, **D**) Real-time cell proliferation analyses for the Cell Index (i.e., the cell-sensor impedance was expressed every two minutes as a unit called "Cell Index"). Results are expressed as means ± SEM of $N = 3$ biological replicates. HEK293T-ACE2 treatment described in the Methods are treated with escalating doses of PI-7 (**C**) or PI-8 (**D**); with vehicle-treated cells were the negative control. Impedance was measured every 2 min over 48 h. The graphs showing "normalized cell index" were generated using Graph Pad Prism 9. (**E**) Caspase-3 activity measured in HEK293T-ACE2 cells with increasing concentrations of compound PI-7 and PI-8 for 18 h. Vehicle-treated cells and cells treated with 10 μM staurosporine are used as negative and positive controls, respectively. Data are presented as relative fluorescent units (RFUs; excitation: 380 nm; emission: 460 nm). Results are expressed as means ± SEM of $N = 3$ biological replicates. NS not significant. One-way ANOVA test among multiple groups, Untreated vs. Vehicle $P = 0.1596$, Vehicle vs. PI-7-1 μM $p = 0.9993$, Vehicle vs. PI-7-10 μM $P = 0.3039$, Vehicle vs. PI-7-100 μM $P = 0.5176$, Vehicle vs. PI-8-1 μM $P = 0.9992$, Vehicle *vs.* PI-8-10 μM $P > 0.9999$, Vehicle vs. PI-8-100 μM $P = 0.9732$), NS not significant. (**F**) A representative immunoblotting analyses on total protein lysates obtained from HEK293T-ACE2 treated with escalating doses of PI-7 (top) and PI-8 (bottom) molecules using antibodies against Cleaved Caspase-3 fragments (17–19 kDa). β-Actin is used as the loading control. Vehicle-treated cells are used as a negative control of the experiment. (**G**) Quantification of relative fluorescence changes of Fluo3-AM as a measure of intracellular Ca^{2+} levels for up to 48 min in HEK293T cells treated with 10 μM of PI-7, vehicle-treated cells were used as negative control. Results are expressed as means ± SEM of $N = 4$ biological replicates. Fd ANOVA global - CH test, $P = 0.007$. (**H**) A cycloheximide (CHX) chase assay, representative immunoblotting analyses on total protein lysates obtained from HEK293T-ACE2 treated with CHX at different time point (from T = 0 to T = 10 h) using antibodies against ATP2B1 and β-Actin used as the loading control. Vehicle-treated cells (i.e., 0.001% DMSO) are used as negative control of the experiment. (**I**) A Representative immunoblotting analyses on total protein lysates obtained from HEK293T-ACE2 treated with CHX and PI-7 for 8 h, using antibodies against ATP2B1. β-Actin is used as loading control. Vehicle-treated cells are used as negative control. Source data are available online for this figure

 © The Author(s)

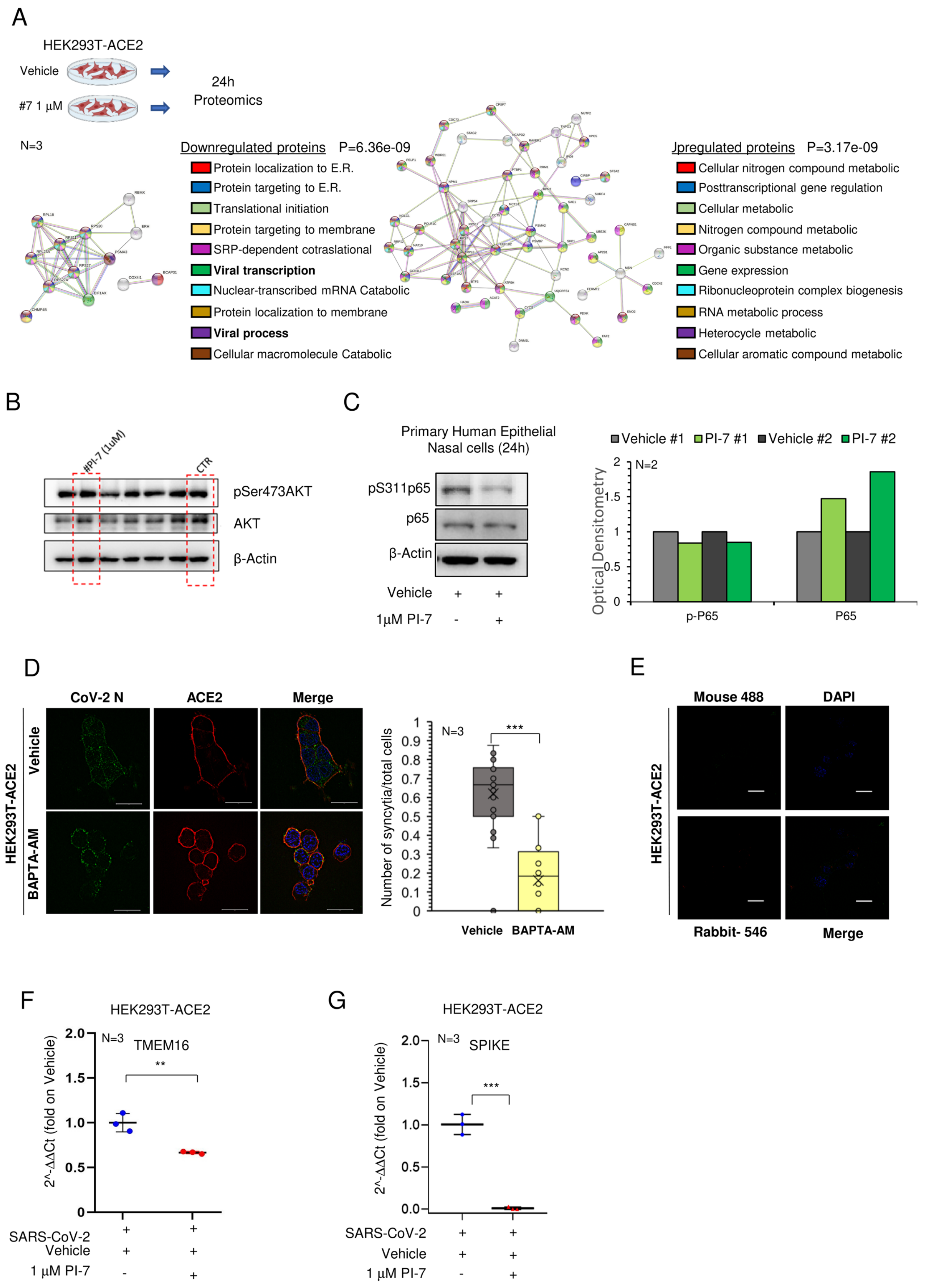
A
HEK293T-ACE2
Vehicle
#7 1 μM
24h
Proteomics
N=3
Downregulated proteins P=6.36e-09
Protein localization to E.R.
Protein targeting to E.R.
Translational initiation
Protein targeting to membrane
SRP-dependent cotraslational
Viral transcription
Nuclear-transcribed mRNA Catabolic
Protein localization to membrane
Viral process
Cellular macromolecule Catabolic
Jpregulated proteins P=3.17e-09
Cellular nitrogen compound metabolic
Posttranscriptional gene regulation
Cellular metabolic
Nitrogen compound metabolic
Organic substance metabolic
Gene expression
Ribonucleoprotein complex biogenesis
RNA metabolic process
Heterocycle metabolic
Cellular aromatic compound metabolic
B
#PI-7 (1uM)
CTR
pSer473AKT
AKT
β-Actin
C
Primary Human Epithelial Nasal cells (24h)
pS311p65
p65
β-Actin
Vehicle + +
1μM PI-7 - +
Vehicle #1 PI-7 #1 Vehicle #2 PI-7 #2
N=2
Optical Densitometry
p-P65
P65
D
CoV-2 N
ACE2
Merge
HEK293T-ACE2
Vehicle
BAPTA-AM
Number of syncytia/total cells
N=3
***
Vehicle BAPTA-AM
E
Mouse 488
DAPI
Rabbit- 546
Merge
HEK293T-ACE2
F
HEK293T-ACE2
N=3
TMEM16
**
2^-ΔΔCt (fold on Vehicle)
SARS-CoV-2 + +
Vehicle + +
1 μM PI-7 - +
G
HEK293T-ACE2
N=3
SPIKE
***
2^-ΔΔCt (fold on Vehicle)
SARS-CoV-2 + +
Vehicle + +
1 μM PI-7 - +

Figure EV5. Compound PI-7 diminishes SARS-CoV-2 replication by affecting viral processes, syncytia formation and inflammatory pathways. Data related to Fig. 5.

(**A**) Top panel: a proteomic assay based on LC-MS/MS approach performed on HEK293T-ACE2 cells treated with PI-7 molecule (1 μM) for 24 h, $N = 3$ biological replicates. Bottom left: A protein interaction network was generated using the Search Tool for the Retrieval of Interacting Genes/ Proteins (STRING) database (https://string-db.org), using only those proteins that were downregulated in PI-7- treated cells within "viral process" and "viral transcription" category functions, in bold (i.e., n.18 downregulated proteins, Appendix 7). One-way ANOVA, $P = 6.36$ e09. N = 3 biological replicates. Bottom right: A protein interaction network was generated using STRING database (https://string-db.org) by using only those proteins found upregulated in PI-7-treated cells (i.e., n.66 upregulated protein, with different category functions, Appendix 8). One-way ANOVA test, $P = 3.17$e09. (**B**) A representative immunoblotting analysis on uninfected cells using antibodies against the pSer-473AKT and AKT proteins on total protein lysates obtained from HEK293T-ACE2 treated with compound PI-7 (1 μM-dashed lines) or vehicle-treated (CTR dashed lines) for 24 h. β-Actin is used as the loading control. (**C**) A representative immunoblotting analysis using antibodies against the pS311–p65 and p65 proteins on total protein lysates obtained from human primary epithelial nasal cells treated with compound PI-7 (1 μM) for 24 h. β-Actin is used as the loading control. Densitometric analysis from $N = 2$ technical replicates. (**D**) IF with an antibody against viral CoV-2 N (green) and human ACE2 (red) proteins in HEK293T-ACE2 cells treated with BAPTA-AM (20 μM) and infected with SARS-CoV-2 for 72 h (i.e., treated as in Fig. 4G). Right: The graph showing the intensity of fluorescence is shown on the left. Data are means ± SD of $N = 3$ biological replicates. Unpaired two-tailed *T* Student test, ***$P < 0.001$. Data measurements values: vehicle-treated min=0.333, max=0.875, center=0.667, bounds of box= 0.5–0.757 and whiskers = 0, percentiles= 0.064 ($K = 0.01$) – 1.122 ($K = 0.99$); BAPTA-AM treated: min=0.0, max=0.5, center=0.183, bounds of box=0–0.312, whiskers=none, percentiles= 0 ($K = 0.01$) – 0.5 ($K = 0.99$). The SIM^2 image are acquired with Elyra 7 (Zeiss) and processed with Zeiss ZEN software (blue edition). Magnification, ×63. Scale bar, 20 μm. (**E**) A representative IF staining with secondary antibodies anti-rabbit Alexa Fluor 546 (1:200; #A10040, Thermo Fisher Scientific) or anti-mouse Alexa Fluor 488 (1:200, ab150113, Abcam) on HEK293T-ACE2 cells. DAPI is used for nuclear staining (blue). The image was acquired with Elyra 7 (Zeiss). Magnification: 40×; Scale bar, 20 μm. (**F**) QPCR of mRNA abundance relative to that in control (CTR) cells ($2^{-\Delta\Delta Ct}$) for human TMEM16 gene. RNA extracted from HEK293T-ACE2 cells treated as in Fig. 4G). Scattered plots show the individual values ad mean as indicated by the horizontal black lines of $N = 3$ biological replicates. Unpaired two-tailed *T* Student test and Bonferroni corrected **$P < 0.01$. (**G**) QPCR of mRNA abundance relative to that in control (CTR) cells ($2^{-\Delta\Delta Ct}$) for SARS-CoV-2 SPIKE gene from HEK293T-ACE2 cells treated as in Fig. 4G. Scattered plots show the individual values ad mean as indicated by the horizontal black lines of $N = 3$ biological replicates. Unpaired two-tailed *T* Student test ***$P < 0.001$. Source data are available online for this figure

 © The Author(s)
